# Evolution and diversification of PAL-mediated salicylic acid biosynthesis in Rosaceae

**DOI:** 10.1093/hr/uhag108

**Published:** 2026-04-02

**Authors:** Qiao-Ling Zhang, Tong-Jian Liu, Yi-Bing Wang, Tian Feng, Lin Chen, Bo-Zhi Xiao, Han-Xiang Li, Hai-Bo Tan, Hui-Run Huang, Xue-Jun Ge, Hai-Fei Yan, Xin-Feng Wang

**Affiliations:** Hangzhou Xixi National Wetland Park Service Center (Hangzhou Xixi National Wetland Park Ecology & Culture Research Center), Hangzhou 310013, China; Zhejiang Xixi Wetland Ecosystem Observation and Research Station, Hangzhou 310013, China; State Key Laboratory of Plant Diversity and Specialty Crops, South China Botanical Garden, Chinese Academy of Sciences, Guangzhou, Guangdong 510650, China; Key Laboratory of National Forestry and Grassland Administration on Plant Conservation and Utilization in Southern China, South China Botanical Garden, Chinese Academy of Sciences, Guangzhou 510650, China; South China National Botanical Garden, Guangzhou 510650, China; State Key Laboratory of Plant Diversity and Specialty Crops, South China Botanical Garden, Chinese Academy of Sciences, Guangzhou, Guangdong 510650, China; Key Laboratory of National Forestry and Grassland Administration on Plant Conservation and Utilization in Southern China, South China Botanical Garden, Chinese Academy of Sciences, Guangzhou 510650, China; South China National Botanical Garden, Guangzhou 510650, China; State Key Laboratory of Plant Diversity and Specialty Crops, South China Botanical Garden, Chinese Academy of Sciences, Guangzhou, Guangdong 510650, China; Key Laboratory of National Forestry and Grassland Administration on Plant Conservation and Utilization in Southern China, South China Botanical Garden, Chinese Academy of Sciences, Guangzhou 510650, China; South China National Botanical Garden, Guangzhou 510650, China; Hangzhou Xixi National Wetland Park Service Center (Hangzhou Xixi National Wetland Park Ecology & Culture Research Center), Hangzhou 310013, China; Zhejiang Xixi Wetland Ecosystem Observation and Research Station, Hangzhou 310013, China; Guangzhou Dublin International College of Life Sciences and Technology, South China Agricultural University, Guangzhou 510642, China; State Key Laboratory of Plant Diversity and Specialty Crops, South China Botanical Garden, Chinese Academy of Sciences, Guangzhou, Guangdong 510650, China; Key Laboratory of National Forestry and Grassland Administration on Plant Conservation and Utilization in Southern China, South China Botanical Garden, Chinese Academy of Sciences, Guangzhou 510650, China; South China National Botanical Garden, Guangzhou 510650, China; State Key Laboratory of Plant Diversity and Specialty Crops, South China Botanical Garden, Chinese Academy of Sciences, Guangzhou, Guangdong 510650, China; Key Laboratory of National Forestry and Grassland Administration on Plant Conservation and Utilization in Southern China, South China Botanical Garden, Chinese Academy of Sciences, Guangzhou 510650, China; South China National Botanical Garden, Guangzhou 510650, China; State Key Laboratory of Plant Diversity and Specialty Crops, South China Botanical Garden, Chinese Academy of Sciences, Guangzhou, Guangdong 510650, China; Key Laboratory of National Forestry and Grassland Administration on Plant Conservation and Utilization in Southern China, South China Botanical Garden, Chinese Academy of Sciences, Guangzhou 510650, China; South China National Botanical Garden, Guangzhou 510650, China; State Key Laboratory of Plant Diversity and Specialty Crops, South China Botanical Garden, Chinese Academy of Sciences, Guangzhou, Guangdong 510650, China; Key Laboratory of National Forestry and Grassland Administration on Plant Conservation and Utilization in Southern China, South China Botanical Garden, Chinese Academy of Sciences, Guangzhou 510650, China; South China National Botanical Garden, Guangzhou 510650, China; State Key Laboratory of Plant Diversity and Specialty Crops, South China Botanical Garden, Chinese Academy of Sciences, Guangzhou, Guangdong 510650, China; Key Laboratory of National Forestry and Grassland Administration on Plant Conservation and Utilization in Southern China, South China Botanical Garden, Chinese Academy of Sciences, Guangzhou 510650, China; South China National Botanical Garden, Guangzhou 510650, China; State Key Laboratory of Plant Diversity and Specialty Crops, South China Botanical Garden, Chinese Academy of Sciences, Guangzhou, Guangdong 510650, China; Key Laboratory of National Forestry and Grassland Administration on Plant Conservation and Utilization in Southern China, South China Botanical Garden, Chinese Academy of Sciences, Guangzhou 510650, China; South China National Botanical Garden, Guangzhou 510650, China

## Abstract

Salicylic acid (SA) is a central phytohormone in plant immunity and stress responses, yet the evolutionary dynamics of its phenylalanine ammonia-lyase (PAL)-mediated biosynthetic route remain poorly understood despite recent biochemical advances. As the original source of SA, *Spiraea* (Rosaceae) holds historical and evolutionary significance for studying SA biosynthesis. Here, we generated a chromosome-level genome assembly of *Spiraea chinensis* and integrated comparative genomics, transcriptomic, and targeted metabolite profiling to investigate the evolutionary diversification of SA biosynthesis across Rosaceae. Phylogenomics places *S. chinensis* in the subfamily Amygdaloideae, diverging from other genera ~57.8 Mya. Extensive chromosome fission–fusion events and lineage-specific whole-genome duplication (WGD) have driven karyotype diversification across Rosaceae. Comparative analyses revealed the PAL-mediated route as the dominant SA biosynthetic pathway across Rosaceae, with WGD-driven expansion in Amygdaloideae and combined WGD- and small-scale duplication (SSD)-derived origins in Rosoideae. WGD-derived PAL-route genes largely retained synteny and stable high expression, whereas lineage-specific SSD-derived paralogs exhibited reduced synteny and variable expression, consistent with post-duplication regulatory divergence and subfunctionalization. Transcriptome analyses revealed pronounced tissue-specific expression of PAL-route genes across Rosaceae, and UPLC–MS/MS profiling further demonstrated differential SA accumulation in *S. chinensis*, with the highest levels in branches (606–1038 ng/g FW), followed by leaves (183–432 ng/g FW) and flowers (42–56 ng/g FW), supporting active SA biosynthesis in both vegetative and reproductive tissues. Collectively, our results establish the PAL-mediated pathway as the primary and evolutionarily conserved route of SA biosynthesis in Rosaceae and demonstrate how genome dynamics and regulatory diversification jointly drive the evolutionary innovation within this pathway.

## Introduction

Salicylic acid (SA) is a central signaling molecule in plants, playing indispensable roles in defense against biotic stresses—particularly through the activation of systemic acquired resistance—as well as in modulating responses to diverse abiotic stressors [1–5]. In response to pathogen infection, SA orchestrates immune activation by promoting defense gene expression and hypersensitive responses, whereas under abiotic stresses such as heat, drought, and salinity, SA contributes to stress acclimation by modulating redox balance and cellular homeostasis [1, 3, 6, 7]. Beyond its canonical defensive role, accumulating evidence indicates that SA functions as a dose- and context-dependent integrator of plant growth, development, and environmental adaptation, coordinating extensive transcriptional reprogramming, redox homeostasis, and hormonal crosstalk [6, 8]. Through interactions with other phytohormones, such as jasmonic acid, ethylene, auxin, and abscisic acid, SA reprioritizes resource allocation between growth and defense, providing a mechanistic basis for growth–defense tradeoffs and shaping plant fitness under fluctuating environments [8–11]. Recent advances using genetically encoded SA sensors further revealed that pathogen invasion triggers rapid, wave-like propagation of SA signals across tissues, highlighting SA as a dynamic, systemically transmitted hormone rather than a purely local defense metabolite [12]. Collectively, these findings position SA as a pivotal hub within plant signaling networks, underscoring the importance of elucidating its biosynthetic regulation and evolutionary diversification to understand plant immunity and environmental adaptation.

The dynamic and context-dependent functions of SA rely on tight, spatiotemporally coordinated regulation of its biosynthesis and downstream signaling machinery, enabling precise control of SA-mediated immune responses [13–18]. In higher plants, SA is synthesized predominantly via two routes originating from chorismate: the isochorismate synthase (ICS) route and the phenylalanine ammonia-lyase (PAL) route. The ICS route has been well characterized in *Arabidopsis thaliana* (L.) Heynh., in which plastid-localized *ICS1* (isochorismate synthase) converts chorismate to isochorismate, followed by cytosolic export via *EDS5* (isochorismate exporter), and subsequent conversion of isochorismate-derived intermediates to SA by *PBS3* (isochorismoyl-glutamate synthase) and *EPS1* (isochorismoyl-glutamate pyruvoyl-glutamate lyase) [14–20].

In contrast, although PAL-mediated SA biosynthesis has long been proposed, its enzymatic framework remained elusive until recently [16, 20]. Breakthrough studies have now delineated a multistep PAL route that integrates reactions across the cytosol, peroxisome, and endoplasmic reticulum (ER) [18, 19, 21–26]. This pathway is initiated by *PAL*-catalyzed deamination of phenylalanine (Phe) to trans-cinnamic acid (*t*-CA) in the cytosol [18, 19, 26], followed by peroxisomal β-oxidation that converts *t*-CA to benzoyl-coenzyme A (benzoyl-CoA) via the coordinated actions of cinnamoyl-CoA ligase (*CNL*), cinnamoyl-CoA hydratase/dehydrogenase (*CHD*), and ketoacyl-CoA thiolase (*KAT*) [18, 19, 21–23]. Benzoyl-CoA is subsequently converted to SA through a series of ester-mediated reactions involving benzyl alcohol benzoyltransferase (*BEBT*) in the peroxisome, oxidation by ER-associated benzyl benzoate oxidase (*BBO*), and final hydrolysis by cytosolic benzyl salicylate hydrolase (*BSH*), with benzyl benzoate and benzyl salicylate acting as key intermediates [18, 19]. These findings reveal that the PAL-mediated route is far more biochemically complex than previously appreciated; however, its evolutionary conservation, diversification, and relative contribution across plant lineages remain largely unexplored.

Historically, *Spiraea*, together with willow bark, was among the earliest plant sources of natural SA and directly inspired the naming of aspirin [27, 28], underscoring a long-standing association between this genus and SA metabolism. *Spiraea*, a genus of woody shrubs in the subfamily Amygdaloideae of Rosaceae, comprises approximately 90 species widely distributed in temperate regions and is valued for both floricultural traits and strong ecological adaptability [29–34]. This unique historical connection, coupled with its woody growth habit, highlights *Spiraea* as an ideal lineage for investigating the evolutionary dynamics of SA biosynthesis in perennial plants, especially ornamentals exposed to diverse and often fluctuating environments. Despite its biological and historical significance, genomic resources for this genus remain extremely limited. To date, only a draft genome of *Spiraea crenata* L. has been reported [35], and no high-quality, chromosome-level reference genome is available. This scarcity of genomic data has constrained functional studies of metabolic pathways, including SA biosynthesis, and has hindered robust phylogenetic and evolutionary analyses within the genus.

At a broader phylogenetic scale, *Spiraea* belongs to Rosaceae, a family that encompasses numerous economically and ecologically important fruit crops (*Malus*, *Prunus*, *Pyrus*, *Fragaria*) as well as ornamentals (*Rosa*, *Photinia*, *Spiraea*) [20, 30, 31, 34, 36]. Extensive genome sequencing efforts across Rosaceae have enabled comparative analyses of diverse metabolic pathways, including vitamin C biosynthesis, sorbitol metabolism, and anthocyanin production [37–45]. In contrast, genes involved in SA biosynthesis remain inconsistently annotated, and their evolutionary trajectories are poorly resolved across many lineages, despite the central role of SA in defense and stress adaptation. Leveraging the rich genomic resources available for Rosaceae, while expanding taxonomic representation to include environmentally resilient ornamentals such as *Spiraea*, offers both the biological relevance and technical feasibility for elucidating how SA-associated gene families have diversified and contributed to lineage-specific adaptive strategies.

**Figure 1:**
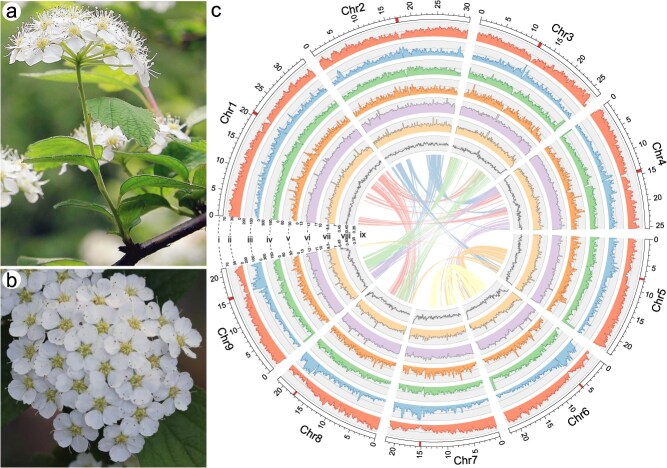
Floral morphology and genomic features of *S. chinensis*. (a and b) Representative flower morphology. (c) Circos plot of the chromosome-level genome assembly. Tracks (from outer to inner) show: (i) nine pseudo-chromosomes with lengths in megabases (Mb), centromeres highlighted in red; (ii) gene density; (iii–v) densities of LTRs, TIRs, and Helitrons; (vi–vii) Illumina and HiFi read coverage; (viii) GC content; and (ix) intra-genomic synteny. All statistics were calculated in 250-Kb sliding windows with a 100-Kb step.

In this study, we generated a chromosome-level genome assembly of the ecologically resilient ornamental shrub *Spiraea chinensis* Maxim. (Chinese spiraea), a woody species native to East Asia and widely distributed across China [31, 46], occupying habitats from lowlands to 500–2400 m [33, 36, 47–49], with profuse white flowers and an extended flowering period (Fig. 1a). This assembly, together with high-quality genomes of representative Rosaceae species, multi-tissue transcriptomes, and targeted metabolite measurements was used to investigate the evolutionary diversification of SA biosynthetic pathways across the family. By integrating comparative genomics with expression and metabolite profiling, we examine how gene duplication, lineage-specific retention, and regulatory divergence have shaped the structural organization and tissue-specific deployment of SA-pathway gene families. These analyses provide evolutionary insights into the diversification of SA metabolism in woody plants and establish *S. chinensis* as a genomic resource for molecular evolutionary studies of hormone biosynthesis.

## Results

### Genome size and ploidy level of *S. chinensis*

To facilitate high-quality genome assembly and downstream evolutionary and functional analyses, we first assessed the ploidy status of the *S. chinensis* accession used in this study. Genome size was estimated using flow cytometry, with *Glycine max* (1C = 1115 Mb) as an internal reference standard. The estimated nuclear DNA content was approximately 513 ± 10.2 Mb per 1C, more than twice the size of most previously reported *Spiraea* genomes (~200 Mb), suggesting tetraploidy in this individual (Fig. S1; Table S1). We further characterized genome properties using a whole-genome survey with Illumina short reads. A total of 29.65 Gb of clean data (~120× coverage relative to the estimated monoploid genome size) was generated for k-mer analysis (Fig. S2; Table S2). GenomeScope2 estimated a monoploid genome size of ~188.24–189.80 Mb, with 38.44%–39.97% repetitive content. Both the 19-mer and 21-mer frequency distributions showed a markedly better fit to the tetraploid model than the diploid model (96.89% vs. 89.06%, and 96.37% vs. 81.77%, respectively) (Tables S3 and S4), supporting a tetraploid genome structure for this accession.

Additionally, ploidy inference using nQuire, which applies a probabilistic model based on base frequency distributions, strongly supported a tetraploid model. The tetraploid model provided the best fit, with delta log-likelihood (∆logL) values of 200522.08 vs. 3044043.70, and 433925.44 vs. 4432860.97 (after denoising) compared to diploid models, respectively, where smaller ∆logL indicates a better fit (Table S5). Collectively, these results are consistent with previous findings identifying tetraploid individuals (2*n* = 4*x* = 36) based on karyotypic analysis [50]. Our data confirm that the *S. chinensis* accession analyzed here is most likely tetraploid and provide a reliable genomic foundation for subsequent assembly and evolutionary inference, offering critical context for interpreting gene copy number variation in pathways of interest.

### Chromosome-level genome assembly and annotation of *S. chinensis*

To generate a high-quality reference genome for *S. chinensis*, we performed a *de novo* assembly using a combination of Illumina short reads (29.65 Gb), PacBio HiFi long reads (20.8 Gb), and Hi-C sequencing data (53.6 Gb) (Table S2), providing sufficient coverage to support chromosome-level genome reconstruction. Using the PacBio HiFi reads as the primary data source, the initial assembly yielded a total of 325 contigs, with a contig N50 of 20.04 Mb (Tables 1 and S6). By incorporating Hi-C data, we anchored the assembly onto nine pseudo-chromosomes ranging from 21.57 to 34.41 Mb in length. Given the tetraploid status of the species (2*n* = 4*x* = 36), the final assembly represents a single haploid chromosome set consisting of nine pseudo-chromosomes, with a total span of 228.11 Mb (Fig. 1; Table 1; Table S7). Read-mapping assessments indicated that 98.47% of the HiFi reads and 95.97% of the Illumina reads aligned to the final reference genome, with uniform coverage across all chromosomes (mean depth = 59×). BUSCO analysis demonstrated high genome completeness, with 97.88% (416/425), 98.14% (1584/1614), and 97.33% (2264/2326) complete orthologs identified in the Embryophyta, Viridiplantae, and Eudicot odb10 datasets, respectively (Table 1; Table S7). The long terminal repeat (LTR) Assembly Index (LAI) reached 32.53, well above the 20 thresholds for a gold-standard plant genome [51], and Merqury high-quality values of 39.28, further supporting the assembly’s high contiguity and completeness. Telomeric repeat sequences (AAACCCT) were detected on 6 of the 9 pseudo-chromosomes, with three (Chr1, Chr3, Chr6) harboring repeats at both ends and three (Chr2, Chr4, Chr5) containing the motif at one terminus (Table S8). In addition, centromeric regions were identified on all nine chromosomes (Fig. 1; Fig. S3; Table S9). Collectively, these metrics establish a high-quality, chromosome-level reference assembly of *S. chinensis*.

**Table 1:** Summary of assembly, assessment, and annotation information for the *S. chinensis* genome.

Feature		*S. chinensis*
	Assembled contigs	325
	Contigs (≥50 000 bp)	89
	Largest contig	26 870 316
	Contig N50 (bp)	20 043 130
	Contig L50	11
	Contig GC content (%)	39.74
	Scaffolds N50 (bp)	25 399 841
	Number of contigs used in pseudo-chromosomes	13
	Cumulative length of pseudo-chromosomes (bp)	228 113 825
	Number of pseudo-chromosomes	9
	Longest pseudo-chromosome (bp)	34 406 275
	Shortest pseudo-chromosome (bp)	21 572 556
Assembly	Genome GC content (%)	39.70
	Completeness BUSCOs (%)	97.33
	LAI	32.53
	Merqury consensus QV	39.28
	Mapping with Illumina reads (%)	95.97
Assessment	Mapping with HiFi reads (%)	98.47
	Repeat contents (%)	36.98
	Number of genes	29 265
	Number of transcripts	33 097
Annotation	Average gene length (bp)	1129

Following the assembly, we performed comprehensive structural and functional annotation of the *S. chinensis* genome. Using an integrated strategy combining transcriptomic evidence from three tissues (branch, leaf, and flower), *ab initio* prediction, and homology-based alignment to Rosaceae and plant protein databases, we identified 29 265 high-confidence protein-coding genes, generating 33 097 transcript isoforms (Table 1; Fig. S3; Tables S7 and S10–S12). The average gene length was 1129 bp, accounting for 15.83% of the genome. Functional annotation assigned putative functions to the majority of genes based on matches in eggNOG-mapper, PANNZER2, Mercator4, and InterProScan (Table S11). Repeat annotation revealed 173 991 transposable elements, occupying 36.95% of the genome, with long terminal repeat retrotransposons (LTRs) being the most abundant class, and tandem repeats totaling 136 181 elements (6.63% of the genome) distributed across all chromosomes (Tables S13 and S14). LTR insertion analysis indicated a recent transpositional burst, suggesting ongoing activity of LTR retrotransposons in the *S. chinensis* genome (Fig. S4). Together, this high-contiguity, well-annotated chromosome-level assembly provides a robust and comprehensive reference for exploring gene and repeat evolution in *S. chinensis*, enabling reliable identification of gene families and comparative analyses, including those involved in specialized pathways.

### Phylogenetic position and genomic evolution of *S. chinensis*

To investigate the phylogenetic placement and evolutionary history of *S. chinensis*, we performed a comparative genomic analysis using genome assemblies from seven species within the subfamily Amygdaloideae and four species from the subfamily Rosoideae of the Rosaceae, as well as ten additional angiosperm species as outgroups (Table S15), along with the genome of *S. chinensis* assembled in this study. Orthologous clustering using OrthoFinder2 identified 41 399 orthogroups across the 22 species, including 4776 core, 6828–13 604 dispensable, and 67–3015 unique gene families (Tables S16 and S17). In *S. chinensis*, we detected 17 605 orthogroups, of which 733 were species-specific, encompassing 2342 unique genes potentially related to lineage-specific functions.

**Figure 2:**
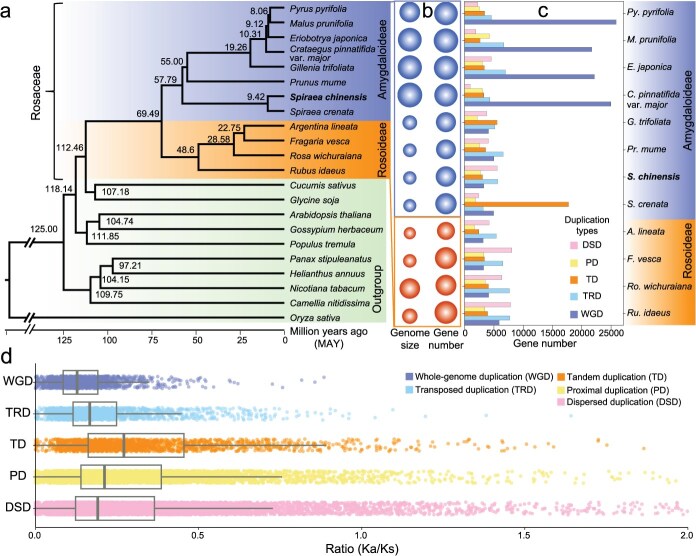
Phylogeny, genome characteristics, and gene duplication modes in Rosaceae. (a) Phylogeny and divergence times of Rosaceae and outgroup species inferred from universal single-copy genes using maximum likelihood method (RAxML-NG), with divergence time estimation by TreePL. Blue, orange, and green shaded areas indicate Amygdaloideae, Rosoideae, and outgroup species, respectively. (b) Genome sizes (187.9–779.2 Mb) and gene numbers (24261–44 995) of Rosaceae species. Amygdaloideae species are marked in blue, and Rosoideae species in orange. (c) Gene numbers derived from five duplication modes: DSD, PD, TD, TRD, and WGD. Species identity is shown on the left, with Amygdaloideae and Rosoideae indicated by blue and orange shading. (d) Boxplot comparison of genome-wide Ka/Ks ratios among different duplication types in *S. chinensis*. Horizontal lines within boxes indicate median values, and boxes represent the interquartile range.

Phylogenetic relationships were inferred using 2026 universal single-copy orthologous genes from BUSCO, employing both the maximum likelihood method (RAxML-NG) and TreePL (Figs 2a and S5). The phylogenetic reconstruction robustly placed *S. chinensis* and *S. crenata* as a basal lineage within the subfamily Amygdaloideae, forming a sister clade to the core genera such as *Prunus*, *Pyrus*, and *Malus*, with an estimated divergence time of approximately 57.79 million years ago (Mya) (Fig. 2a; Table S18). The divergence between the two *Spiraea* species was estimated to have occurred around 9.42 Mya (Fig. 2a). This phylogenetic placement is consistent with the traditional taxonomic classification of *Spiraea* within Amygdaloideae and supports previous phylogenomic findings on the evolutionary relationships within the Rosaceae family [36, 42, 43].

Across the 12 Rosaceae species analyzed, genome sizes ranged from 187.9 to 779.2 Mb, and gene numbers varied markedly from 24 261 to 44 995 (Fig. 2b; Table S15). A significant positive correlation was observed between genome size and gene number (Pearson’s *P* value <.01) (Fig. 2b; Fig. S6; Table S15). Further analyses revealed that gene number was also significantly positively correlated with the number of genes derived from proximal duplications (PD) and whole-genome duplications (WGD) (*P* value <.05) (Fig. S6). Notably, the composition of gene duplication modes differed between the Amygdaloideae and Rosoideae subfamilies. Within Amygdaloideae, four recently diverged species—*Pyrus pyrifolia* (Burm. F.) Nakai, *Malus prunifolia* (Willd.) Borkh., *Eriobotrya japonica* (Thunb.) Lindl., and *Crataegus pinnatifida* var. *major* N. E. Br.—specifically retained a large number of WGD-derived genes (21728–25 874), accounting for 48.29% to 61.92% of their total genes (Fig. 2c; Table S19). By contrast, *S. crenata* exhibited a distinct pattern characterized by extensive expansion of tandemly duplicated (TD) genes, with 17 755 copies representing 52.01% of its gene content. In *S. chinensis*, however, the predominant sources of duplicated genes were dispersed duplication (DSD) and transposed duplication (TRD), which accounted for 18.87% and 19.14% of the total, respectively (Fig. 2c; Table S19). Meanwhile, species within Rosoideae exhibited a relatively conserved pattern, in which DSD and TRD represented the predominant duplication modes. Genes derived from DSD and TRD accounted for 17.23%–22.35% and 18.09%–23.48% of the total gene content, respectively (Fig. 2c; Table S19).

Functional enrichment analysis across the 12 Rosaceae species revealed that WGD- and TRD-derived genes generally retain broad functional diversity (Fig. S7a and b), whereas PD- and TD-derived genes are disproportionately enriched in stress-related functions (Fig. S7c–f; Table S20). We further evaluated evolutionary rates—nonsynonymous (Ka), and synonymous (Ks) substitution rates, as well as Ka/Ks ratios—of genes derived from WGD and small-scale duplications (SSDs, including DSD, PD, TD, and TRD) across Rosaceae species. The analysis showed that WGD-derived genes exhibited lower Ka/Ks ratios, with the majority displaying Ka/Ks < 1, indicative of strong purifying selection and functional constraint (Figs S8–S19). This pattern was particularly pronounced in *S. chinensis*, where nearly all WGD-derived gene pairs had Ka/Ks < 1, with only a single pair exceeding 1 (Figs 2d and S14). In contrast, SSD-derived genes, particularly PD- and TD-derived copies, displayed relatively elevated Ka/Ks ratios, reflecting relaxed selective constraints and an increased potential for lineage-specific functional divergence (Figs 2d and S8–S19). Collectively, these results indicate that distinct duplication mechanisms, from WGD to SSDs, are subject to differential selective constraints, thereby contributing to lineage-specific genome evolution and shaping functional diversification across Rosaceae species.

### Chromosome evolution of Rosaceae species

**Figure 3:**
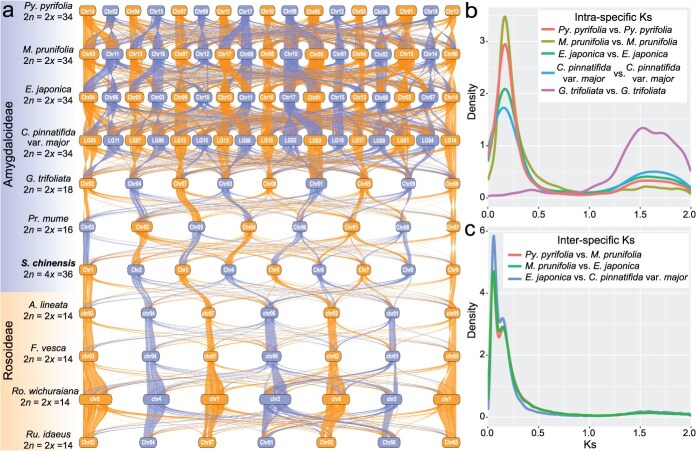
Genomic synteny and divergence patterns in Rosaceae. (a) Genomic synteny relationships among Rosaceae species. Species names and karyotypes are shown on the right, with Amygdaloideae and Rosoideae indicated by blue and orange shading. (b) Ks distributions of paralogous genes within each species, based on collinear gene pairs identified from intragenomic synteny. (c) Ks distributions of homologous genes between species, based on collinear gene pairs identified from interspecific synteny.

While phylogenomic reconstruction clarified the placement of *S. chinensis* within the Rosaceae, genome-scale phylogenies alone are insufficient to resolve the structural mechanisms underlying lineage diversification. In Rosaceae, considerable variation in basic chromosome numbers (*x*) and genome sizes across lineages suggests that chromosomal rearrangements have played a central role in genome evolution (Fig. 2; Table S15). To investigate the structural basis of karyotype evolution in Rosaceae, we conducted comparative chromosomal synteny analysis using high-quality genome assemblies from 11 Rosaceae species, excluding the draft genome of *S. crenata* (Fig. 3a; Table S15). These species display considerable variation in their basic chromosome numbers (*x* = 7–17), providing a valuable phylogenetic framework for exploring chromosomal rearrangements and genome evolution across the Rosaceae family. Syntenic blocks were identified by comparing the positions, orientations, and order of homologous gene pairs. This analysis allowed the reconstruction of putative chromosomal rearrangements—including inversions, translocations, fusions, and fissions—and facilitated the inference of chromosome-specific evolutionary trajectories.

Overall, interchromosomal synteny between *S. chinensis* and the ten other Rosaceae species was limited, indicating extensive genome-wide structural divergence (Fig. 3a; Table S21). Within the subfamily Rosoideae, however, the chromosome base number (*x* = 7) remains conserved, with chromosomes exhibiting largely one-to-one collinearity. An exception is *Rosa wichuraiana* Crép., which experienced a reciprocal translocation between chromosomes chr2 and chr3, resulting in the exchange of chromosome segments (Fig. 3a). By contrast, members of the Amygdaloideae display more variable chromosome base numbers (*x* = 8–17). As a basal lineage of Amygdaloideae, *S. chinensis* carries a base number of nine, which is relatively close to that of Rosoideae (*x* = 7). The additional two chromosomes can be attributed to differences in chromosomal correspondence: two chromosomes of *S. chinensis* (Chr4 and Chr8) align with a single chromosome in Rosoideae species, whereas four chromosomes (Chr5, Chr6, Chr7, and Chr9) align with three in Rosoideae (Fig. 3a). This pattern points to complex chromosome fission–fusion events during the evolutionary history of Rosaceae species.

Within the Amygdaloideae, *Prunus mume* Siebold & Zucc. and *Gillenia trifoliata* (L.) Moench have chromosome base numbers of 8 and 9, respectively, which are close to that of *S. chinensis* (*x* = 9) (Fig. 3a). The difference of one chromosome between *P. mume* and the other two species can be explained by chromosomal correspondence: three chromosomes of *P. mume* (Chr01, Chr06, and Chr08) align with four chromosomes of *G. trifoliata* (Chr01, Chr05, Chr08, and Chr09) and *S. chinensis* (Chr5, Chr6, Chr7, and Chr8) (Fig. 3a). In contrast, four other Amygdaloideae species with higher chromosome base numbers (*x* = 17)—*Py. pyrifolia*, *M. prunifolia*, *E. japonica*, and *C. pinnatifida* var. *major*—differ from the three low-base number species (*G. trifoliata*, *P. mume*, and *S. chinensis*), which generally exhibit a one-to-two collinearity between chromosomes. This pattern is consistent with the hypothesis that the high-base-number species underwent a WGD (Fig. 3a). To test this, we calculated synonymous substitution rates (Ks) within and between species and detected a recent Ks peak (~0.17) shared by the four high-base-number species, whereas their interspecific divergence peaks are much lower (Fig. 3b and c). These results strongly support the occurrence of a WGD event in their ancestral lineage. Moreover, *G. trifoliata*, from the sister clade most closely related to these four species, lacks a recent Ks peak, indicating that the WGD occurred after its divergence from the common ancestor of the four species (Fig. 3b and c). Collectively, these chromosomal fission–fusion events and lineage-specific WGD have profoundly reshaped genome architecture in Rosaceae, providing a structural basis for the diversification of gene content and regulatory landscapes underlying key metabolic pathways.

### Evolutionary diversification of the SA pathway in Rosaceae

**Figure 4:**
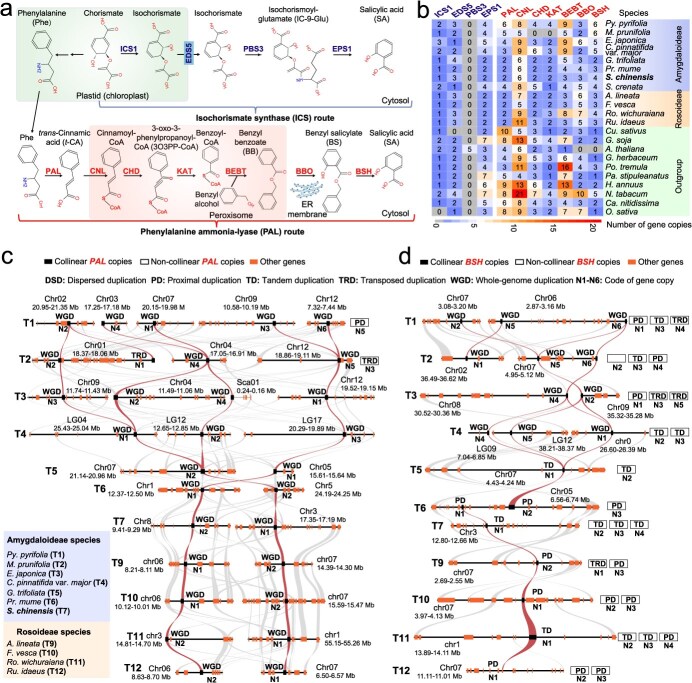
Evolution of SA biosynthetic pathways in Rosaceae. (a) Model of the PAL and ICS routes for SA biosynthesis in plants. (b) Copy numbers of PAL- and ICS-route genes across Rosaceae and outgroup species. Species identity is shown on the left, with Amygdaloideae, Rosoideae, and outgroup species shaded in blue, orange, and green, respectively. (c and d) Local synteny of *PAL* and *BSH* gene copies in Rosaceae, illustrating their evolutionary trajectories. Each row represents the corresponding genomic regions from one species, with species abbreviations shown on the left and full species information in the lower-left corner. Syntenic *PAL* and *BSH* genes are shown as black blocks, non-syntenic *PAL* and *BSH* genes as open black boxes on the right, and other neighboring genes in orange. Red curves connect syntenic *PAL* and *BSH* genes, while gray curves connect other collinear pairs. Gene copy IDs (N1–N6) and duplication modes (WGD, TRD, DSD, TD, PD) are annotated near each gene.

Building on the gene duplication and chromosomal rearrangement patterns described above, chromosomal fission–fusion events, WGD, and small-scale duplications (SSDs) have generated substantial genetic novelty in Rosaceae, shaping not only global genome architecture but also the genetic repertoire of specific functional pathways. To explore how these genomic processes have influenced metabolic diversification, we focused on the SA biosynthesis pathway as a representative stress- and defense-related metabolic system in Rosaceae. Using *A. thaliana* and *Oryza sativa L.*—two model species with well-characterized SA regulatory networks—as references, we identified conserved and divergent pathway components across Rosaceae (Table S22) [17, 18]. Candidate enzyme-coding genes were identified through an integrative framework combining gene family classification, phylogenetic analysis, and subcellular localization prediction across Rosaceae and representative outgroup genomes (see Materials and Methods). Within the biosynthetic module, we systematically investigated both the PAL and ICS routes (Fig. 4a; Figs S20–S30; and Tables S22–S26). Interestingly, no orthologs of the *A. thaliana PBS3* gene were detected in Rosaceae or other outgroups, whereas the key PAL route genes *BBO* and *BSH* were absent from *A. thaliana* (Fig. 4b; Table S25). These findings align with previous reports indicating that the ICS route for SA biosynthesis is largely restricted to Brassicaceae, while most other plants, including Rosaceae, predominantly rely on the PAL route [18, 19].

Comparative analysis revealed that the PAL route and its key enzyme-coding genes are broadly conserved across Rosaceae. However, both gene copy number and duplication origin varied considerably among species (Figs 4b and S31–S37; Tables S26–S29). Most PAL route genes originated from WGD and tandem duplication (TD) events, which together accounted for 33.82% and 23.99% of the copies, respectively. For example, 86.11% (31 of 36) of the identified *PAL* gene copies were WGD-derived (Fig. 4c; Fig. S31; Table S27). Nevertheless, WGD was not the predominant source for all enzyme-coding genes in this route. Distinct duplication mechanisms contributed to their expansion, reflecting evolutionary diversity. For instance, most *BEBT* copies arose from TD, whereas *BSH* copies mainly originated from TD and PD (Fig. 4d; Figs S35 and S37; Table S27). These findings suggest that although WGD has been the major force shaping the repertoire of PAL route genes, other duplication modes, including PD, TD, TRD, and DSD, have also made important contributions to the diversification of enzyme families in this route. Further analysis revealed that WGD-derived gene copies largely retained syntenic relationships across species, whereas most copies arising from lineage-specific SSDs—including PD, TD, TRD, and DSD—displayed little or no conserved synteny (Figs 4c–d and S31–S37).

We further examined SA pathway genes across the two Rosaceae subfamilies and identified distinct duplication patterns, with considerable variation in the origin of SA biosynthetic genes (Figs S31–S37; Tables S28–S29). In the subfamily Amygdaloideae, WGD was the predominant source for all key enzyme-coding genes, except for *BEBT*, which primarily originated from TD (Table S28). Specifically, species that have experienced recent WGD event (i.e. *Py. pyrifolia*, *M. prunifolia*, *E. japonica*, and *C. pinnatifida* var. *major*) retained a large proportion (43.48%–62.50%) of SA pathway genes through WGD, consistent with their genome-wide enrichment of WGD-derived genes (Fig. 2; Table S29). In contrast, the subfamily Rosoideae exhibited more heterogeneous duplication patterns across enzyme genes: *PAL* genes were exclusively WGD-derived, *CHD* and *KAT* copies mainly originated from TRD; *BEBT* primarily from TRD and TD; and *BBO* and *BSH* largely from PD (Fig. 4c and d; S31–S37; Table S28). These contrasting patterns indicate that the SA biosynthetic pathway has undergone subfamily-specific evolutionary trajectories within Rosaceae.

Notably, although *S. chinensis* is taxonomically classified within Amygdaloideae, it exhibited a distinct duplication profile of SA pathway genes compared with other members of this subfamily. In *S. chinensis*, *BSH* copies were solely TD-derived, whereas *PAL* and *KAT* copies originated from WGD, and *CNL* and *BEBT* were mainly derived from PD (Fig. 4c and d; Table S29). By contrast, the closely related congeneric diploid *S. crenata* retained a substantially large proportion of SA pathway genes through TD (67.74%), with copies of *CNL*, *CHD*, *BEBT*, *BBO*, and *BSH* predominantly originating from TD events, reflecting its genome-wide enrichment of TD-derived genes (Fig. 2; Table S29). Consistent with these contrasting duplication compositions, GeneRax analysis identified markedly fewer lineage-specific duplication events in *S. chinensis* than in *S. crenata* (7 vs. 26 duplications; Figs S38–S47; Table S30). Among Amygdaloideae species, *S. crenata* exhibited the highest number of lineage-specific duplications, the majority of which were associated with TD events. Together, these results reveal pronounced lineage-specific differences in the duplication composition and recent expansion dynamics of SA pathway genes between *S. chinensis* and *S. crenata*, mirroring their divergent genome duplication histories.

### Tissue-specific regulation of SA biosynthesis in *S. chinensis*

The evolutionary analyses outlined above revealed that, despite overall conservation of the PAL route in Rosaceae, *S. chinensis* harbors a distinct duplication pattern of key enzyme-coding genes, suggestive of lineage-specific innovation. To investigate whether these genomic features correspond to transcriptional and regulatory divergence, we characterized tissue-specific expression patterns of SA biosynthetic genes by integrating transcriptomic data from branches, leaves, and flowers.

**Figure 5:**
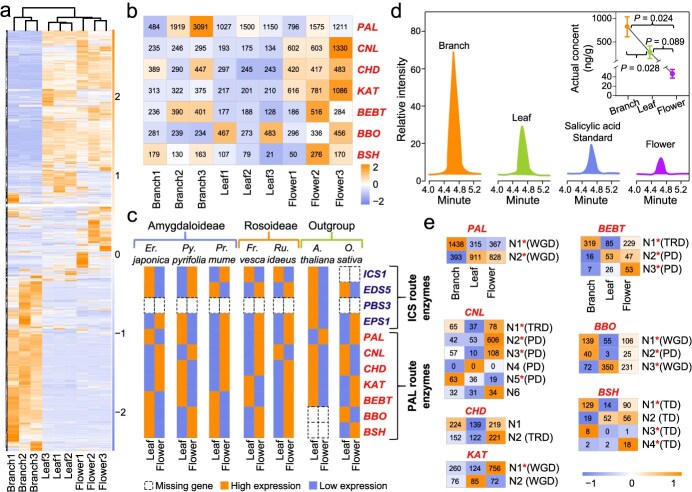
Genome-wide expression patterns and SA biosynthetic enzyme profiles across Rosaceae species. (a) Tissue-specific genome-wide expression heatmap for *S. chinensis*. Expression values are normalized by gene (row-wise Z-score), with orange and blue indicating relatively high and low expression levels, respectively. (b) Cumulative expression levels of enzymes in the PAL-derived SA biosynthetic pathway in *S. chinensis*. Values shown in each cell represent the summed CPM of all gene copies corresponding to each enzyme across tissues. (c) Cumulative expression profiles of ICS- and PAL-route SA biosynthetic enzymes across representative species of Amygdaloideae, Rosoideae, and outgroup taxa. Dashed boxes indicate missing genes. (d) UPLC–MS/MS multiple reaction monitoring chromatograms of SA (transition m/z 137.1 → 65.3) detected in branch, leaf, and flower tissues of *S. chinensis*. The inset shows SA levels across the three tissues (ng/g fresh weight; mean ± SD, *n* = 3), with differences assessed using a two-tailed Student’s *t*-test. (e) Expression patterns of individual gene copies involved in the PAL-derived pathway in *S. chinensis*. Gene names are shown above each panel, gene copy IDs on the right, and DEGs among tissues are indicated by asterisks. The inferred duplication origin of each copy is indicated in parentheses (PD, proximal duplication; TD, tandem duplication; TRD, transposed duplication; WGD, whole-genome duplication).

Global transcriptome profiling uncovered pronounced tissue-specific expression signatures (Fig. 5a; Table S31). Hierarchical clustering showed that biological replicates clustered tightly by tissue, while branches, leaves, and flowers were clearly separated, underscoring strong transcriptional specialization. Comparative analyses further identified 4915 differentially expressed genes (DEGs) between leaf and branch, representing 16.79% of the 29 265 annotated genes, including 2452 up-regulated (upDEGs) and 2463 down-regulated (downDEGs) in leaf (Fig. S48; Tables S32–S35). In flower versus branch, 2950 DEGs were detected (1141 upDEGs and 1809 downDEGs), whereas flower–leaf comparisons yielded 4709 DEGs (1898 upDEGs and 2811 downDEGs) (Fig. S48). Notably, all seven enzyme-coding genes involved in the PAL route of SA biosynthesis were represented among these DEGs (Fig. 5b and c; Table S36).

The seven core enzymes (*PAL*, *CNL*, *CHD*, *KAT*, *BEBT*, *BBO*, and *BSH*) of the PAL-derived SA biosynthetic pathway exhibited pronounced tissue-specific yet partially overlapping transcriptional patterns (Fig. 5a and b; Fig. S49; Table S36). At the upstream entry point of the pathway, *PAL* showed consistently high expression across all examined tissues, with particularly elevated transcript levels in branches, consistent with its central role in channeling metabolic flux into the SA biosynthesis. Several mid-pathway enzymes, including *CNL*, *CHD*, and *KAT*, displayed preferential expression in flowers. By contrast, downstream enzymes exhibited more divergent tissue specificity. *BEBT* maintained relatively high expression across all three tissues, with moderately higher transcript levels in branches and flowers, suggesting a broadly active role in downstream SA metabolism. In comparison, *BBO* expression was enriched in leaves, whereas *BSH* showed the lowest transcript levels in leaves, indicating functional divergence among downstream branches across different tissues.

Notably, tissue-specific regulation of SA biosynthetic genes was also observed in other species from Amygdaloideae and Rosoideae (Fig. 5c; Tables S37–S44), suggesting that organ-level partitioning of SA biosynthesis represents a recurrent regulatory feature across Rosaceae, although the specific genes exhibiting elevated expression differ among species. For comparison, we further examined SA pathway gene expression in the model species *A. thaliana*, which predominantly relies on the ICS route, and *O. sativa*, which primarily utilizes the PAL-derived route (Fig. 5c). In *A. thaliana*, all ICS-route genes exhibited higher expression in leaves than in flowers. In contrast, PAL-route enzymes in *O. sativa* displayed heterogeneous tissue-specific expression patterns, reflecting distinct regulatory strategies associated with PAL-dependent SA biosynthesis.

Because transcript abundance does not necessarily correlate with metabolite accumulation, we next quantified free SA levels in different tissues of *S. chinensis* using UPLC–MS/MS analysis (see Materials and Methods). SA levels were highest in branches (606–1038 ng/g fresh weight [FW]), followed by leaves (183–432 ng/g FW), whereas flowers contained relatively lower levels (42–56 ng/g FW) (Fig. 5d; Table S45). This pattern is consistent with the elevated expression of downstream SA biosynthetic genes in branches, supporting enhanced SA production in this tissue. These metabolite data provide independent support for the observed tissue-specific regulation of SA biosynthesis. Notably, SA concentrations in branches and leaves of *S. chinensis* exceeded those previously reported in leaves of *A. thaliana*, tomato (*Solanum lycopersicum* L.), tobacco (*Nicotiana tabacum* L.), and corn (*Zea mays* L.) (10–100 ng/g FW), but were lower than those detected in leaves of *O. sativa,* willow (*Salix babylonica* L.), soybean (*Glycine max* (L.) Merr.), and tea (*Camellia sinensis* (L.) Kuntze) (1–11 μg/g FW) [23, 52–54].

At the gene level, paralogs exhibited pronounced expression divergence across Rosaceae species. Some gene copies showed strong tissue-specific expression while remaining nearly silent in other tissues, whereas other paralogs were consistently lowly expressed across all tissues examined (Fig. 5e; Fig. S49; Tables S36 and S38–S42). For example, in *S. chinensis*, two *PAL* paralogs displayed contrasting transcriptional patterns, with one predominantly expressed in branches and the other exhibiting minimal expression (Fig. 5e). Similarly, subsets of *CNL* and *BSH* paralogs were enriched in flowers, whereas others were branch-preferential or transcriptionally undetectable (Fig. 5e). Notably, all six WGD-derived gene copies maintained relatively high expression levels, whereas PD-, TD-, and TRD-derived copies showed markedly heterogeneous expression profiles, ranging from strong to nearly undetectable expression (Fig. 5e). Comparable patterns of paralog expression divergence were observed across other Rosaceae species (Tables S38–S42), although the specific tissue preferences and expression patterns varied among lineages, suggesting that duplication mechanism is generally associated with transcriptional divergence, while the resulting expression outcomes are lineage- and gene-specific.

## Discussion

### A chromosome-level genome of *S. chinensis* provides a foundation for dissecting SA biosynthesis in Rosaceae

We report the first chromosome-level genome assembly of *S. chinensis*, a woody ornamental lineage with unique evolutionary and historical relevance in SA biology [27, 28]. As one of the earliest documented botanical sources of natural SA, *Spiraea* has long been associated with the biochemical origin of this pivotal defense hormone. However, the lack of a high-quality reference genome has previously constrained evolutionary and mechanistic analyses of SA biosynthesis in this genus.

Compared with the previously available draft genome of *S. crenata* [35], the *S. chinensis* assembly presented here shows substantially improved contiguity (scaffold N50: 7.69 vs. 25.40 Mb), completeness (BUSCO: 96.00% vs. 97.33%; LAI: 32.53 vs. NA; QV: 39.28 vs. NA), and annotation accuracy for both gene families and repetitive elements. This chromosome-level resolution enables robust reconstruction of chromosomal architecture, duplication history, and gene family evolution—features that are essential for dissecting complex, multistep metabolic pathways such as the PAL-mediated route of SA biosynthesis.

High-quality reference genomes have proven instrumental in linking genome dynamics to metabolic innovation across plants, including ascorbate accumulation, sorbitol metabolism, and anthocyanin production in Rosaceae, as well as vitamin C biosynthesis in kiwifruit, starch metabolism in rye, disease resistance and pericarp dehiscence in banana, and trait dissection enabled by telomere-to-telomere grape genomes [37–40, 55–59]. In this context, the *S. chinensis* genome not only expands genomic resources for Rosaceae ornamentals but also provides a critical evolutionary anchor for tracing the origin, retention, and diversification of SA biosynthetic genes across the family. Beyond SA biology, this resource establishes a foundation for broader investigations into genome evolution, gene duplication, and metabolic pathway diversification in woody ornamentals.

### Evolutionary diversification of SA biosynthesis in Rosaceae

Comparative analyses across representative Rosaceae species indicate that the PAL-mediated route, rather than the ICS route, represents the predominant pathway for SA biosynthesis in this family. This pattern is consistent with broader angiosperm trends, in which the ICS route predominates in Brassicaceae, whereas most other lineages rely primarily on PAL-mediated SA production [18, 19].

The conservation of core PAL-route enzymes suggests strong evolutionary constraint on overall pathway architecture across Rosaceae. Consistent with this interpretation, Ka/Ks analyses revealed that all SA-associated genes exhibit Ka/Ks ratios <1, indicating pervasive purifying selection and functional conservation (Figs S50–S55; Tables S36 and S38–S42). Domain analyses further demonstrate that most Rosaceae SA gene copies retain canonical domains conserved with *O. sativa* (Fig. 5e; Figs S20–S30), underscoring structural stability.

Despite this conserved framework, evolutionary trajectories differ markedly among pathway components, revealing a hierarchical organization within the SA biosynthetic network. Upstream enzymes, particularly *PAL*, have predominantly expanded through WGD, especially within Amygdaloideae. As the gateway enzyme linking primary metabolism to the phenylpropanoid pathway, *PAL* occupies a central and dosage-sensitive position in plant metabolism [60]. Its preferential retention following WGD is consistent with the dosage-balance hypothesis, which predicts the selective maintenance of highly connected or stoichiometrically constrained genes in order to preserve network integrity and metabolic balance [60, 61].

In contrast, downstream or peripheral enzymes such as *BEBT* and *BSH* have expanded primarily through small-scale duplications (SSDs), including tandem (TD), proximal (PD), and TRD. These components likely operate in more modular contexts, allowing SSD-derived paralogs to diverge with minimal disruption to core pathway flux. Supporting this, a subset of SA-related genes—particularly those derived from SSDs (e.g. PD and TD)—exhibits relatively elevated (yet <1) Ka/Ks values (Figs S50–S55), suggesting relaxed selective constraints that may facilitate lineage- or tissue-specific specialization and diversified metabolic outputs.

Subfamily comparisons further illustrate differential remodeling of this pathway, with WGD shaping SA gene architecture predominantly in Amygdaloideae, whereas SSDs contribute more extensively in Rosoideae. Together with the pronounced transcriptional divergence observed among paralogs, these lineage-specific evolutionary patterns demonstrate how a deeply conserved biosynthetic pathway can be differentially remodeled through alternative duplication strategies. Collectively, these findings support a structured evolutionary stratification of the SA biosynthetic network: WGD-driven retention stabilizes central metabolic hubs to preserve basal flux and pathway robustness, while SSD-mediated expansion promotes diversification of peripheral modules, enhancing regulatory flexibility and adaptive potential [60, 61].

Given the central role of SA in plant immunity, recurrent biotic and abiotic stresses likely shaped the hierarchical organization of the PAL-mediated pathway. We acknowledge that a major limitation of the present study is the absence of targeted stress treatments. Without stress-induced experiments, it is not possible to determine which paralogs exhibit rapid inducibility under specific selective pressures, nor to directly link stress-responsive transcriptional dynamics with SA accumulation. The current study was therefore designed to establish a necessary baseline of evolutionary history and tissue-specific expression patterns under non-induced conditions.

Future studies should incorporate stress treatments with correlation analyses between gene expression dynamics and metabolic profile changes to refine comparative analyses and deepen evolutionary inferences. Such experiments will be particularly valuable for evaluating whether SSD-expanded paralogs, such as *BEBT* and *BSH*, exhibit enhanced inducibility relative to WGD-retained core components like *PAL*.

### Lineage-specific duplication modes and transcriptional divergence of the PAL route in *S. chinensis*

The lineage-specific duplication of PAL-route genes in *S. chinensis* exemplifies how genome dynamics can remodel a conserved metabolic pathway. Core enzymes are predominantly retained following WGD and evolve under strong purifying selection, reflecting their essential, dosage-sensitive roles within the phenylpropanoid network. In contrast, downstream components have undergone extensive SSDs, indicating that central and peripheral pathway enzymes are subject to distinct selective pressures (Fig. S50) [60, 61].

This asymmetric duplication pattern is accompanied by pronounced transcriptional divergence among paralogs, providing a flexible regulatory framework for partitioning SA biosynthesis across tissues. Tissue-preferential expression appears to result from subfunctionalization and, in some cases, nonfunctionalization, allowing individual gene copies to specialize while maintaining overall pathway integrity. Such regulatory plasticity may enable context-dependent deployment of SA metabolism in response to developmental and environmental cues.

By maintaining strong functional constraint of core enzymes while diversifying downstream regulators, *S. chinensis* achieves a balance between robust basal SA production and flexibility in modulating pathway output. These findings support a general evolutionary principle in plant metabolism, whereby differential duplication and paralog specialization enable adaptive diversification without compromising core biochemical functions.

### Regulatory plasticity and ecological significance of the PAL route genes

Widespread expression of PAL-route genes across branches, leaves, and flowers indicates that Rosaceae maintains a constitutively active capacity for SA biosynthesis across multiple tissues. In contrast, the tissue-preferential expression of PAL-route genes reveals pronounced regulatory plasticity, suggesting that the PAL-mediated SA biosynthetic pathway is subject to organ-specific modulation to accommodate distinct physiological and ecological demands, rather than producing uniform metabolic outputs.

Notably, in *S. chinensis*, preferential expression of several mid- and downstream PAL-route genes (e.g. *CNL*, *CHD*, *KAT*) in flowers does not consistently coincide with elevated local SA accumulation, indicating an apparent uncoupling between transcriptional activity and metabolite abundance. Importantly, lower SA levels in reproductive tissues do not imply limited biological significance. Accumulating evidence across diverse plant species demonstrates that SA participates in multiple aspects of reproductive development, including stamen development, pollen germination, pollen-tube growth, and ovule development [62–65]. In perennial Rosaceae, exogenous SA application has been shown to promote flower bud differentiation and flowering in *Malus* × *domestica*, further implicating SA in floral developmental regulation [66, 67].

However, because PAL-route enzymes occupy central positions within the phenylpropanoid network and supply precursors to multiple downstream branches—including lignin, flavonoids, anthocyanins, coumarins, stilbenes, and volatile benzenoids—transcript abundance alone cannot fully resolve metabolic flux partitioning [16, 21, 23, 68]. Core enzymes such as *PAL* function as metabolic hubs, channeling phenylalanine-derived carbon into nearly all major phenylpropanoid classes [68]. By contrast, downstream enzymes exhibit varying degrees of pathway specificity. For instance, *CNL* preferentially directs cinnamate-derived intermediates toward benzoic acid and SA formation [23], whereas *CHD* and *KAT* contribute more broadly to benzenoid metabolism. *CHD* catalyzes the hydration–dehydrogenation steps of the β-oxidative route [16, 21], while *KAT* supplies benzoyl-CoA for both benzoic acid biosynthesis and volatile benzenoid production [16, 22].

Consistent with this multifunctional framework, recent studies in chrysanthemum demonstrated that SA signaling can modulate floral fragrance production via an NPR3–WRKY1 regulatory module, linking SA perception to secondary metabolite output [13]. Together, these observations indicate that the flower-biased expression of PAL-route genes in *S. chinensis* is more plausibly associated with the coordinated activation of multiple phenylpropanoid-derived functions during reproductive development, rather than representing a strict specialization toward SA biosynthesis. In this context, the apparent decoupling between gene expression patterns and SA accumulation is better explained by differential metabolic partitioning among competing phenylpropanoid branches, rather than by intrinsic limitations in SA biosynthetic capacity.

## Conclusions and future directions

*Spiraea chinensis* provides a valuable genomic reference for investigating the evolution of SA-associated metabolic pathways in woody plants. Within Rosaceae, PAL-mediated SA biosynthesis exhibits a combination of strong structural conservation and evolutionary flexibility, driven by lineage-specific gene duplication, transcriptional divergence, and tissue-specific regulatory modulation. By integrating high-quality genome assemblies from representative Rosaceae species with transcriptomic analyses and targeted metabolite quantification, this study demonstrates how alternative duplication strategies and regulatory diversification can differentially remodel core and peripheral components of this conserved metabolic network, thereby contributing to metabolic innovation in woody plants.

Nevertheless, transcript abundance alone cannot fully resolve metabolic flux or explain how divergence among PAL-route paralog translates into functional outcomes. In particular, the regulation and biological significance of elevated PAL-route expression in reproductive tissues remain unclear.

A comprehensive understanding of PAL-mediated SA biosynthesis will require expanded metabolite profiling, spatially resolved transcriptomics, and functional assays tracing phenylalanine-derived carbon allocation among competing phenylpropanoid branches. Integration of targeted stress-induction experiments with multi-omic analyses will be essential to clarify how duplication mode, regulatory divergence, and network topology jointly shape functional specialization of individual paralogs. Together, these approaches will provide a robust framework for exploring the evolutionary and functional diversification of SA biosynthesis and related phenylpropanoid traits in woody plants.

## Materials and methods

### Genome survey and profiling

The genomic characteristics of the *S. chinensis* were investigated using flow cytometry and k-mer analysis. For flow cytometry, we followed previously described protocols [69], using soybean (*Glycine max* var. Williams 82; 1115 Mb) as the internal standard. Fresh leaves were chopped and incubated in suspension buffer for 5 min, followed by staining with propidium iodide (PI) for 30 min. After RNA digestion with RNase A, the nuclei suspensions were analyzed using a Sysmex CyFlow Cube 6 flow cytometer. The genome size of each sample was estimated based on the mean fluorescence intensity, which is positively correlated with DNA content.

For genome survey analysis, total genomic DNA was isolated from silica gel-dried leaves. A whole-genome shotgun (WGS) library was constructed with an insert size of 300–500 bp and sequenced on the Illumina HiSeq 4000 system. A k-mer frequency spectrum was then generated using Jellyfish v2.3.0 [70] with 150 bp × 2 paired-end reads. Given the conflicting reports from previous karyotype analyses of *S. chinensis* (2*n* = 2*x* = 18 or 2*n* = 4*x* = 36) [47, 50], we assessed the ploidy level using nQuire [71]. nQuire uses a Gaussian Mixture Model to characterize the distribution of base frequencies at biallelic sites. Evaluation metrics, including ∆*ln*L, normalized *SSR*, *y–y* slope, and *r* [2], were calculated for three fixed models (diploid, triploid, and tetraploid) alongside a free model. In addition, genome profiling was performed using GenomeScope2 [72] with a tetraploid mixture model to estimate key genomic features, such as size, heterozygosity, and repeat content.

### Genome sequencing and assembly

Young, fresh leaves of *S. chinensis* were collected from the Hangzhou Botanical Garden and frozen in liquid nitrogen for both next-generation (NGS) and third-generation long-read sequencing (TGS) library construction. For long-read sequencing, total genomic DNA was extracted using a modified megabase-sized nuclear DNA extraction method [73]. DNA integrity and quality were assessed using agarose gel electrophoresis. A PacBio HiFi library was prepared and then sequenced on the PacBio Sequel II system (Annoroad Gene Technology, Beijing), producing circular consensus sequencing (CCS) reads. To achieve chromosome-level scaffolding, fresh leaf tissue was fixed with 1% formaldehyde to generate a Hi-C library. Chromatin was digested using the HindIII restriction enzyme, followed by the ligation of spatially proximate fragments labeled with biotin. The Hi-C library was sequenced on an Illumina NovaSeq 6000 system, producing 2 × 150 bp paired-end reads.

To assemble a high-quality reference genome, CCS reads with QV < 20 were filtered out, and the remaining high-quality reads were designated as HiFi reads. The initial assembly was performed using hifiasm v0.16.1 [74] in both standalone mode and Hi-C integrated mode to resolve haploid contigs. The primary assembly graph was further examined using Bandage [75], to identify and remove potential redundant or heterozygous regions. For scaffolding, Hi-C reads were mapped to filtered contigs using BWA-MEM [76]. Contigs with strong linkage signals were clustered and ordered using Juice and the 3D-DNA pipeline [77]. The final assembly was visualized as a Hi-C heatmap in Juicebox, resulting in the successful construction of nine pseudo-chromosomes. Remaining intra-scaffold gaps were filled using Gapfiller v1.11 [78]. Telomeric regions were identified by detecting Rosales-specific motifs at both ends of each pseudo-chromosome using TIDK v0.2.31 (https://github.com/tolkit/telomeric-identifier).

Putative centromeric regions were identified by searching for the characteristic tandem repeats. The Phobos output was manually screened for the most abundant tandem repeats (unit length 80–350 bp) in each pseudomolecule. These repeats were used to mask the pseudo-chromosomes in 100-Kb intervals using RepeatMasker. Regions with high repeat density, further characterized by the presence of LTR Chromovirus elements, were defined as putative centromeres. Pericentromeric boundaries and repeat compositions were further recognized using Centromics v0.3 (https://github.com/zhangrengang/Centromics).

Assembly quality was rigorously validated using multiple metrics. Minimap2 v2.26 [79] was used to map both PacBio HiFi reads and Illumina paired-end reads back to the assembly to assess read coverage and consistency. Genome completeness was evaluated using BUSCO v5.4.3 [80] against the Viridiplantae, Embryophyta, and Eudicot orthology datasets (OrthoDB v10), with comparison to other high-quality Rosaceae genomes. Additionally, the LTR Assembly Index (LAI) was calculated using EDTA v2.1.0 ^81^ to assess the structural integrity of *S. chinensis* assembly (with LAI > 20 indicating gold-standard quality [51]). Finally, the consensus accuracy was estimated using the Merqury [82] consensus quality value (QV).

### Characterization of repetitive elements

To identify and annotate repetitive elements, two distinct databases were generated: one for genomic masking prior to gene prediction, and another for high-quality structural annotation. For the masking database, *de novo* repeat prediction was performed using RepeatModeler v2.0.3 [83] with default parameters. The resulting library was merged with the Viridiplantae-specific repeats extracted from the RepBase library v20181026 [84]. This comprehensive library was then subsequently utilized by RepeatMasker v4.1.2-p1 [85] to identify repetitive regions, yielding a soft-masked genome (where repeats are denoted in lowercase) for downstream analyses.

For a more rigorous annotation of transposable elements (TEs), a specialized library was constructed using the Extensive de-novo TE Annotator v2.1.0 [81]. EDTA integrates multiple *ab initio* and homology-based discovery tools, including: LTRharvest [86] and LTR_retriever [87] for long terminal repeat retrotransposons (LTR-RTs), TIR-Learner [88] for terminal inverted repeat (TIR) transposons, and Helitronscanner [89] for Helitrons. After consolidating these outputs, a high-quality, non-redundant TE library was generated by systematically filtering out false positives and redundant elements.

### Gene prediction and functional annotation

Protein-coding gene was annotated using an integrated pipeline combining *ab initio* searching, homology-based prediction, and transcriptomic evidence. For transcriptomic evidence, three RNA-seq libraries (branch, leaf, and flower) were pooled and aligned to the soft-masked *S. chinensis* genome using Hisat2 [90]. We executed two independent runs of BRAKER2 [91] in both RNA-seq and protein pipeline mode. In the RNA-seq mode, spliced alignments were used to train the gene prediction tools GeneMark-EP+ [92] and AUGUSTUS [93]. In the protein mode, gene structures were predicted using the extended OrthoDB library as extrinsic evidence. Finally, the transcript- and protein-derived gene models (GTF files and hint files) were integrated and filtered by TSEBRA [91] which weighed different evidences to produce the final consensus gene set. Non-coding RNAs (ncRNAs), including tRNAs, miRNAs, snRNAs, and snoRNAs, were identified using the Infernal v1.1.4 [94] against the Rfam 14 database [95]. Additionally, ribosomal RNAs (5S, 5.8S, 18S, and 28S) were predicted using Barrnap v0.9 (https://github.com/tseemann/barrnap).

Functional annotation for protein-coding genes were performed via three online services: eggNOG-mapper v2.1.9 [96] with the eggNOG v5.0 database [97], PANNZER2 [98], and Mercator4 (v5.0) [99]. Complementarily, InterProScan v5.62–94.0 [100] was employed to InterPro IDs and protein domains. These results were consolidated to assign Gene Ontology (GO) [101] terms and Kyoto Encyclopedia of Genes and Genomes (KEGG) pathways [102]. Furthermore, transcription factors (TFs) were identified and classified using iTAK [103], followed by a comparative analysis across multiple Rosaceae species.

### Comparative genomics and evolutionary analysis

Genomic protein sequences of Rosaceae and representative outgroup species were retrieved from the Genome Database for Rosaceae (GDR), SoyBase, and TIAR database (Table S15) [104, 105]. To identify gene families across all nine species, we performed all-to-all BLAST searches using Orthofinder v2.5.2 ^106^ with inflation parameter of 1.5 to infer orthologous groups (OGs). Individual gene trees for all OGs and a reconciled species tree were constructed using the STAG algorithm [107]. Gene duplication events, inferred using the DLC (Duplication-Loss-Coalescence) method, were mapped onto each node of the species tree. Species-specific genes were defined as those with no identifiable homologs in other sampled genomes. GO and KEGG enrichment analyses were conducted using ShinyGO v0.77 [108], and a *p*-value <0.05 was used as the significance threshold.

To determine the phylogenetic relationships, we used BUSCO to identify universal single-copy orthologs across all 22 species, filtering out genes with <80% coverage. Coding sequences from 2026 single-copy genes were extracted and concatenated for phylogenomic reconstruction using RAxML-NG v1.0.3 ^109^ under the GTR + GAMMA model. Based on the inferred topology, TreePL [110] was used to estimate divergence times, utilizing six secondary calibration points (Table S18). TreePL analysis was conducted using a treepl_wrapper.sh script (https://github.com/tongjial/treepl_wrapper) following a three-step workflow: (i) The primary analysis was iterated 100 times to identify the most frequent optimal cross-validation (CV) parameters; (ii) cross-validation was performed to determine the smoothing value that yielded the lowest CV score; and (iii) the final chronogram was generated using the optimized smoothing value.

### Synteny analysis and duplication detection

WGD events were investigated by searching collinearity between and within Rosaceae species using MCScanX [111]. BLAST hits with an e-value <1 × 10^−10^ and alignment coverage >50% were retained for syntenic block identification. The resulting syntenic relationships were visualized using the online tool ChiPlot (https://www.chiplot.online/) and JCVI (Java Comparative Genomics Toolkit) [112].

To estimate the timing of duplication events, synonymous substitution rates (*K*s) were calculated for duplicate gene pairs within collinear blocks. Genomic sequences were aligned for each pair, and *K*s values was calculated using the YN method implemented in KaKs_Calculator v2.0 [113]. Gene duplication modes were identified using DupGen_finder [61], which classifies genes into five duplication types: DSD, PD, TD, TRD, and WGD.

### Phylogenetic reconstruction and gene tree reconciliation of SA-pathway gene families

A total of 416 gene copies belonging to 10 SA-pathway gene families were identified across eight Amygdaloideae genomes. Protein sequences were aligned using MAFFT [114], and poorly aligned regions were trimmed with trimAl [115]. Maximum-likelihood phylogenetic trees were then reconstructed using RAxML-NG v1.0.3 [109]. The best-fitting amino acid substitution model for each family was determined according to the Bayesian Information Criterion (BIC). Topological support was assessed with 500 bootstrap replicates.

To investigate the evolutionary history of these families, gene tree reconciliation analyses were performed for each SA-pathway gene family using GeneRax under the UndatedDTL model [116], with the species phylogeny provided as a topological constraint. The UndatedDTL model treats gene duplication, loss, and horizontal transfer (DTL) as Poisson processes occurring along the branches of the species tree. Model parameters were optimized by maximum likelihood, allowing simultaneous refinement of gene tree topologies and the precise inference of evolutionary events, including gene duplications, losses, and transfers.

### Identification of SA-related genes

Genes involved in SA biosynthesis and signaling pathway are curated based on previous studies (Table S22) [14, 17, 18]. Protein sequences of these SA related gene were retrieved as references to identify homologs in *S. chinensis* and other plant genomes using MMSeqs2 with the default easy-search mode. To refine the identification and reduce potential overestimation caused by reliance on sequence homology alone, orthology relationships were inferred using Orthofinder2 [106], and candidate SA-related genes were subsequently validated within the corresponding orthogroups (OGs).

To further enhance functional annotation confidence, candidate SA biosynthetic genes were subjected to subcellular localization prediction using DeepLoc-2.1 [117]. Only gene copies with predicted localizations consistent with known or proposed cellular sites of the ICS- and PAL-mediated SA biosynthesis pathways were retained (Fig. 4a and b; Table S24). Conversely, genes localized to compartments inconsistent with SA biosynthesis were considered likely paralogs involved in other phenylpropanoid branches and were excluded from downstream analyses.

In addition, gene family–specific phylogenetic trees were reconstructed using RAxML-NG v1.0.3 ^109^ with the GTR + GAMMA model, incorporating functionally characterized reference genes. Within the Rosaceae clade, only gene copies that clustered within well-supported orthologous clades were retained. Divergent paralogs, potentially reflecting neofunctionalization toward other metabolic processes, were excluded. Phylogenetic relationships were interpreted together with subcellular localization to distinguish SA pathway orthologs from divergent paralogs. Finally, conserved domain architectures were annotated using the NCBI Conserved Domain Database (CDD; https://www.ncbi.nlm.nih.gov/Structure/bwrpsb/bwrpsb.cgi) and visualized as bar plots. All phylogenetic trees and domain architecture plots were visualized using TBtools-II [118].

### Differential expression and comparative transcriptomic analyses

Clean reads from three tissues (branch, leaf, and flower) of *S. chinensis*, generated from cDNA libraries were mapped to the reference genome using the STAR splicing aligner [119]. For each tissue type, three biological replicates were analyzed. Gene-level counts were quantified using FeatureCounts [120], and expression levels were normalized as CPM (counts per million) values. Differential gene expression (DGE) analysis was performed using DESeq2 [121]. Genes were considered as significantly differentially expressed based on false discovery rate (FDR) < 0.05 and |log_2_FoldChange| > 1. Expression heatmaps were generated in R using the pheatmap package.

To facilitate cross-species comparative analyses, additional transcriptome sequencing data from flower and leaf tissues of representative Amygdaloideae and Rosoideae species were systematically retrieved from public databases, with *O. sativa* and *A. thaliana* serving as outgroups (Table S37). All transcriptomic datasets were processed using a standardized analytical pipeline to generate normalized CPM expression matrices.

To mitigate batch effects and biological noise, transcriptomic datasets incorporated in this study were subjected to comprehensive literature curation. The original publications were carefully examined to verify sample origin, growth conditions, and experimental treatments. Samples exposed to abiotic stresses (e.g. drought, high salinity, or extreme temperatures) or biotic stresses (e.g. pathogen inoculation) were excluded. Only transcriptomes derived from plant tissues grown under normal conditions were retained, thereby minimizing potential environmental influences on gene expression patterns.

### Extraction and quantification of SA

Samples from three tissues (branch, leaf, and flower) of *S. chinensis* were collected, with three biological replicates prepared for each tissue. Each replicate consisted of approximately 500 mg of fresh tissue. Samples were lyophilized using an FDU-2100 freeze dryer and subsequently ground into a fine powder. The powdered material was subjected to ultrasonic extraction with methanol using a KQ5200DE ultrasonic cleaner at 25°C for 30 min. Specifically, 1.0 ml of methanol was used for flower samples, whereas 1.5 ml of methanol was used for branch and leaf samples. The resulting extracts were filtered through a 0.22 μm microporous membrane prior to UPLC–MS/MS analysis.

SA was quantified using a Thermo Scientific TSQ Endura UPLC–MS/MS system equipped with a UPLC Hypersil Gold column (100 × 2.1 mm, 1.9 μm; Thermo Scientific). The column temperature was maintained at 40°C, with a flow rate of 0.2 ml/min and an injection volume of 1 μl. The gradient elution program was as follows: 0–8 min, 25% to 30% B; 8.1–11 min, hold at 90% B; and 11.1–15 min, hold at 25% B. Mass spectrometry was performed in negative ion mode with the following parameters: ion source temperature, 250°C; sheath gas flow, 35 arb; auxiliary gas flow, 10 arb; ion transfer tube temperature, 275°C; and spray voltage, 2.5 kV. Quantification was conducted in multiple reaction monitoring mode using the transition m/z 137.1 → 65.3, with a collision energy of 27.0 eV and an RF lens voltage of 75 V.

## Supplementary Material

Web_Material_uhag108

## Data Availability

The raw sequencing data, including Illumina short reads, HiFi reads, Hi-C reads, and RNA short reads, have been deposited in the Genome Sequence Archive (GSA) at the National Genomics Data Center (NGDC), China National Center for Bioinformation (CNCB) under BioProject PRJCA046475 with the accession number CRA030184 [122, 123]. The final genome assembly and annotation files are available in the Genome Warehouse (GWH) at NGDC under the same BioProject (accession SAMC5926829) [124], and have also been deposited in the Figshare (https://doi.org/10.6084/m9.figshare.30143092). In addition, the genome assembly has been deposited in the National Center for Biotechnology Information (NCBI) under the BioProject PRJNA1330000 with accession number JBRBAJ000000000.
