# Supplementary material for: Evolution and diversification of PAL-mediated salicylic acid biosynthesis in Rosaceae
Source: Hortic Res. 2026 Apr 2;13(7):uhag108. doi: 10.1093/hr/uhag108 (PMC13293624; doi:10.1093/hr/uhag108)
Supplement: Web_Material_uhag108 [file web_material_uhag108.zip › 002Revised supplemental figures (clean version)_final.docx]

**
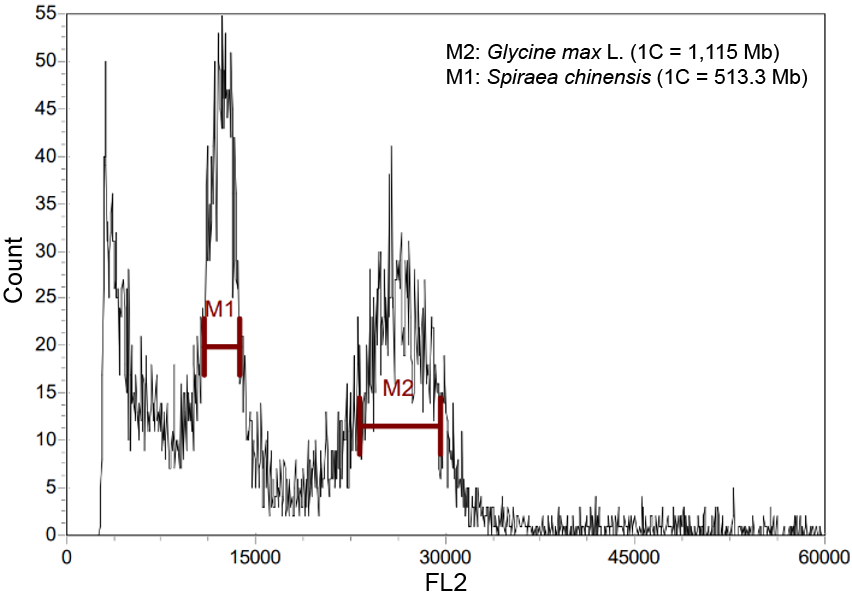
**

**Figure S1. Flow cytometry histogram for the genome size estimation of *S. chinensis*, relative to the internal standard *Glycine max* L..**

**
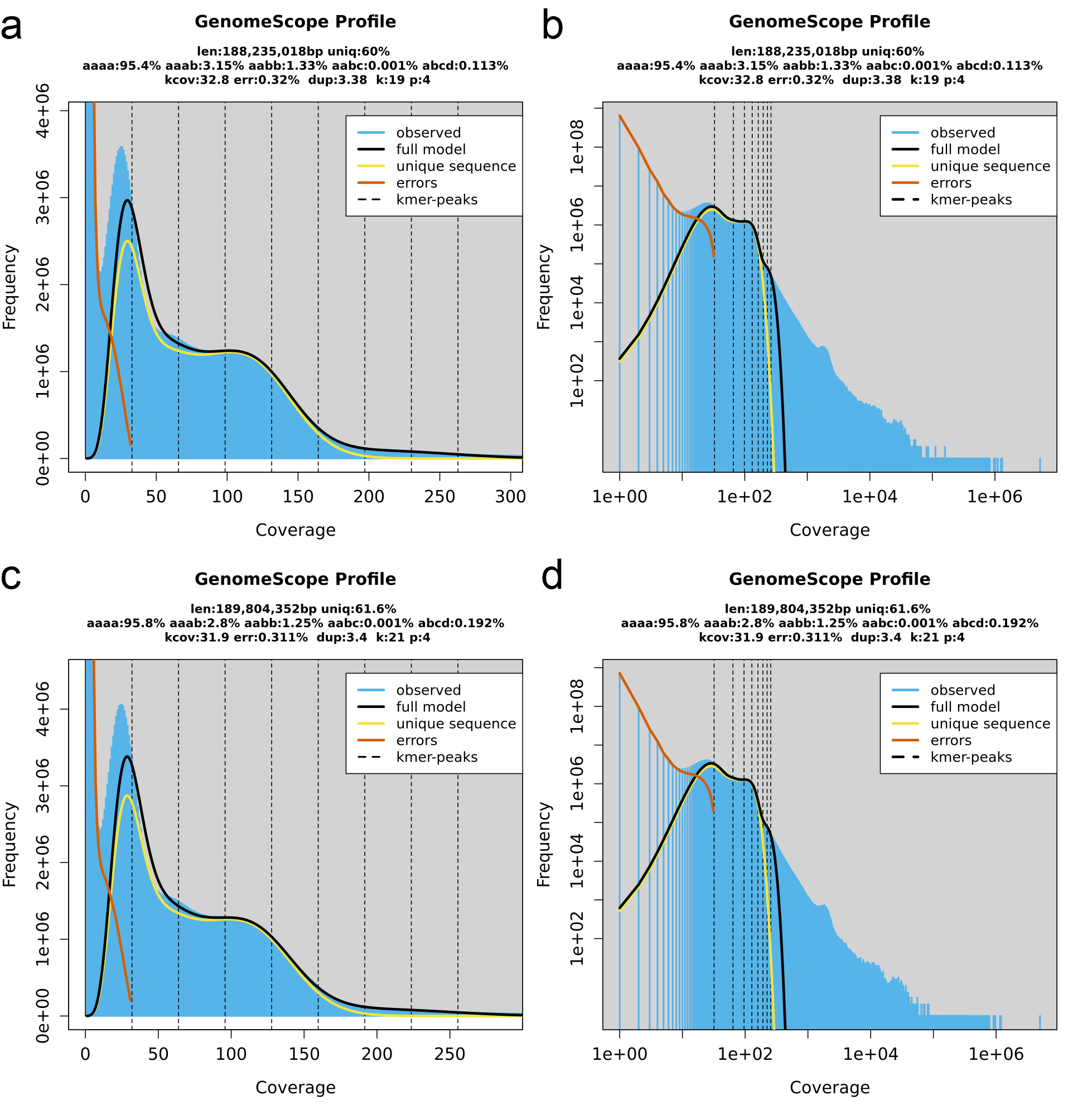
**

**Figure S2. K-mer frequency spectrum (k=19 and k=21) for the *S. chinensis* genome survey.** Both spectra exhibit peaks that align well with the predicted tetraploid model (p = 4).

**
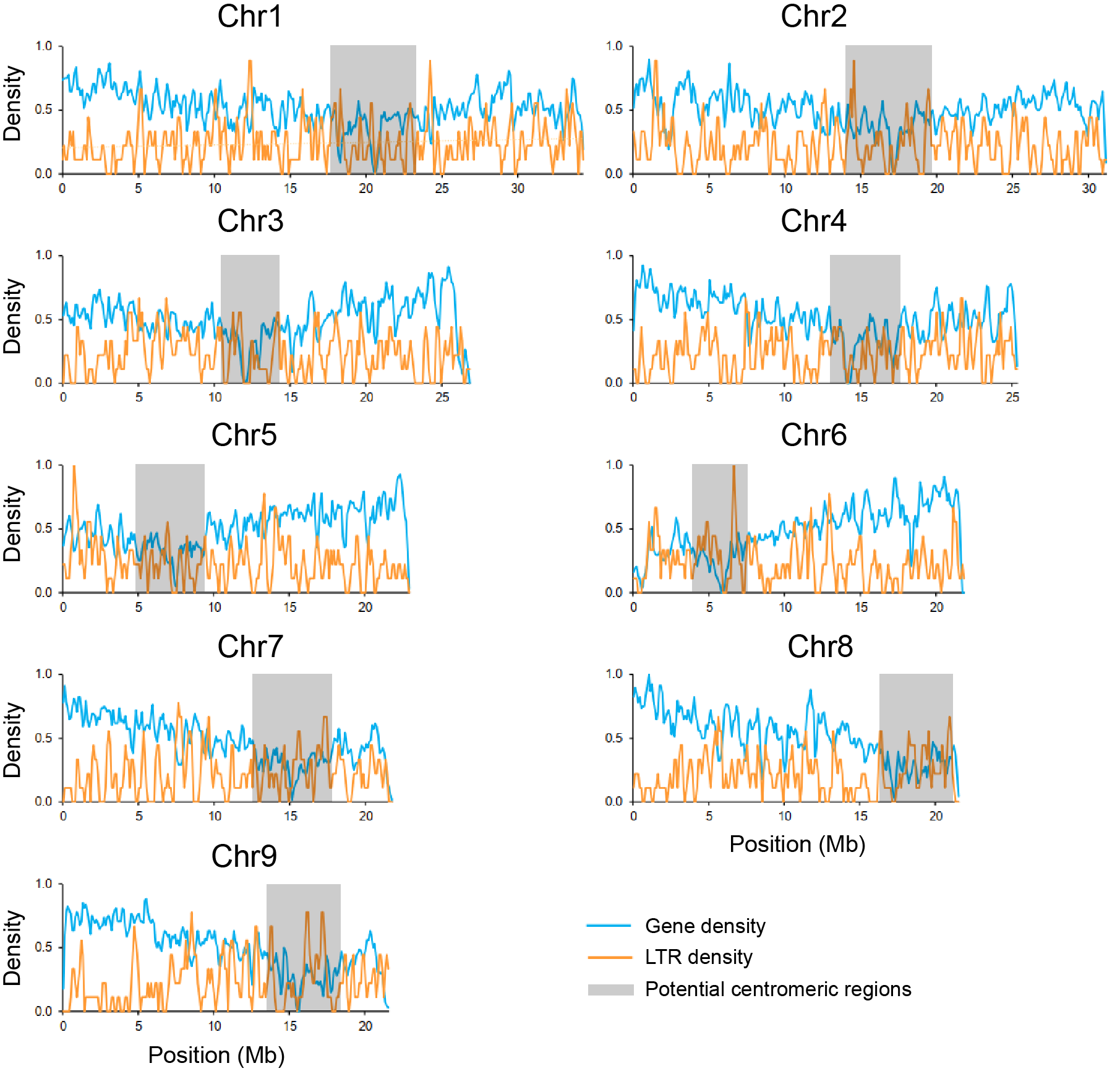
**

**Figure S3. Genomic landscape of gene and long terminal repeat retrotransposon (LTR) density across the nine pseudo-chromosomes of *S. chinensis*.** Shaded gray regions indicate the putative centromeric regions for each pseudo-chromosome.

**
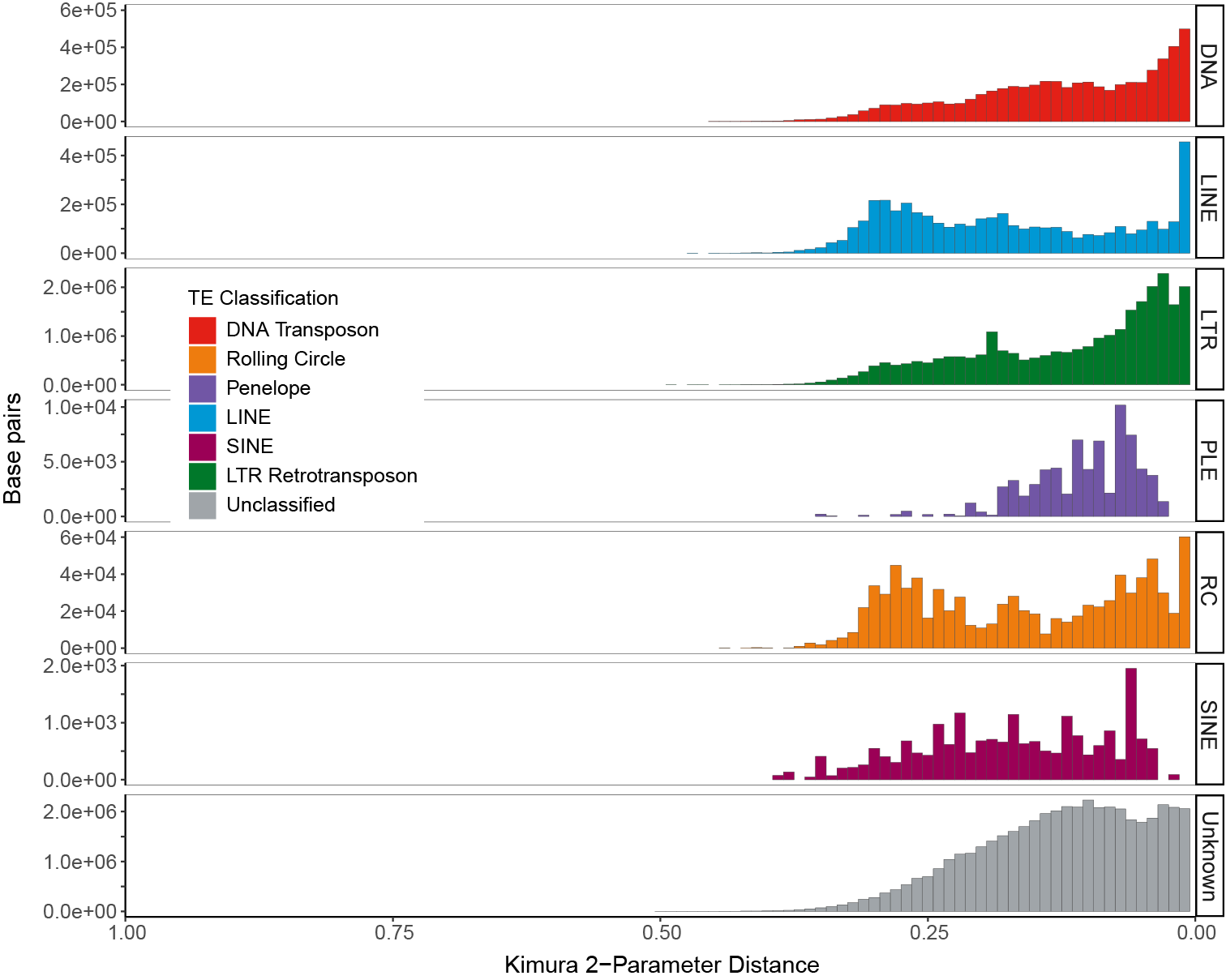
**

**Figure S4. Kimura 2-Parameter (K2P) divergence distribution of transposable elements (TEs) in the *S. chinensis* genome. Distances represent the sequence divergence of individual TE copies from their respective consensus sequences.**


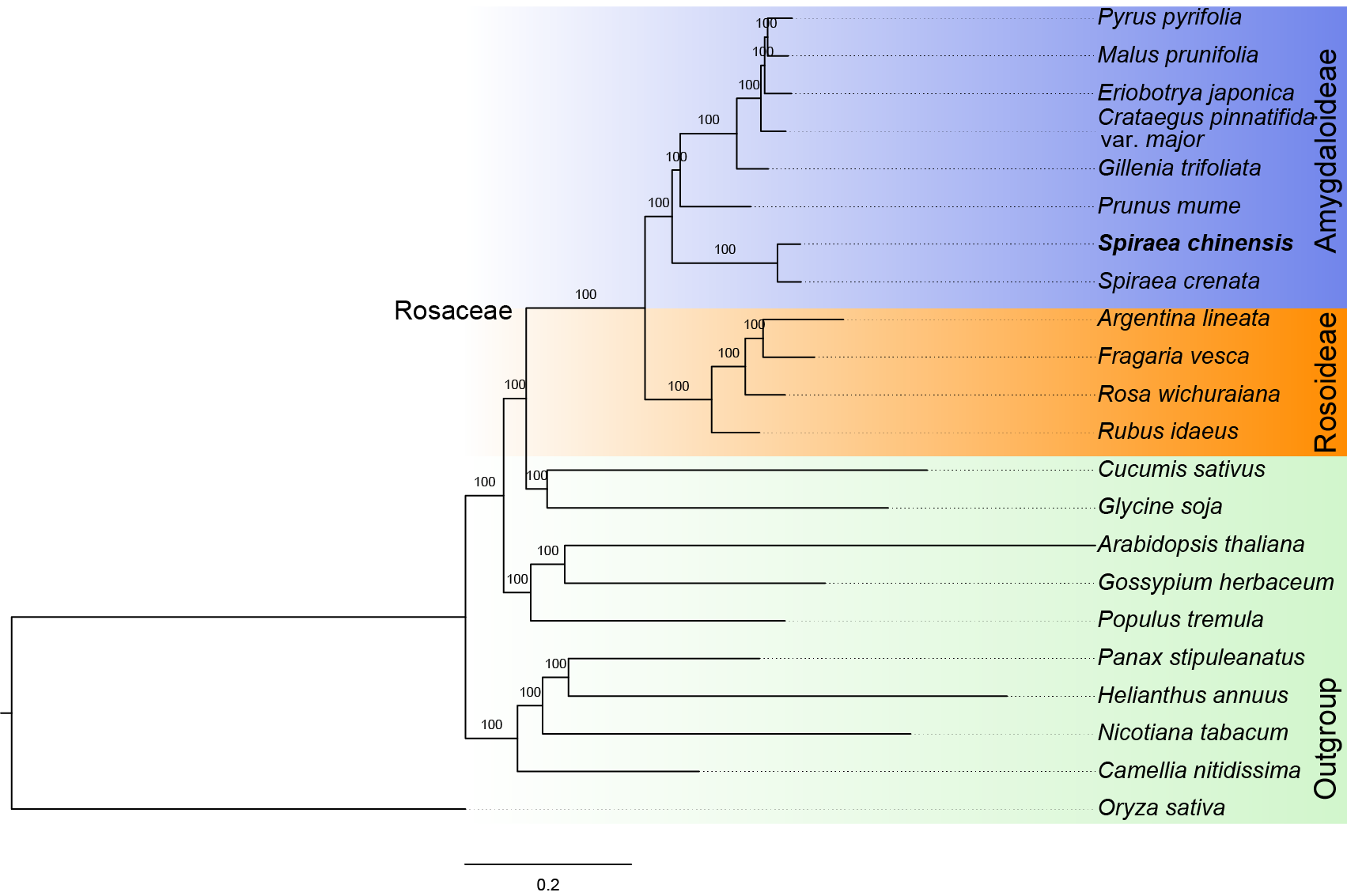


**Figure S5. Phylogenetic relationships of Rosaceae and outgroup species inferred using RAxML-NG.** The maximum likelihood tree was constructed based on 2,026 single-copy orthologous genes. Bootstrap support values are shown at each node.


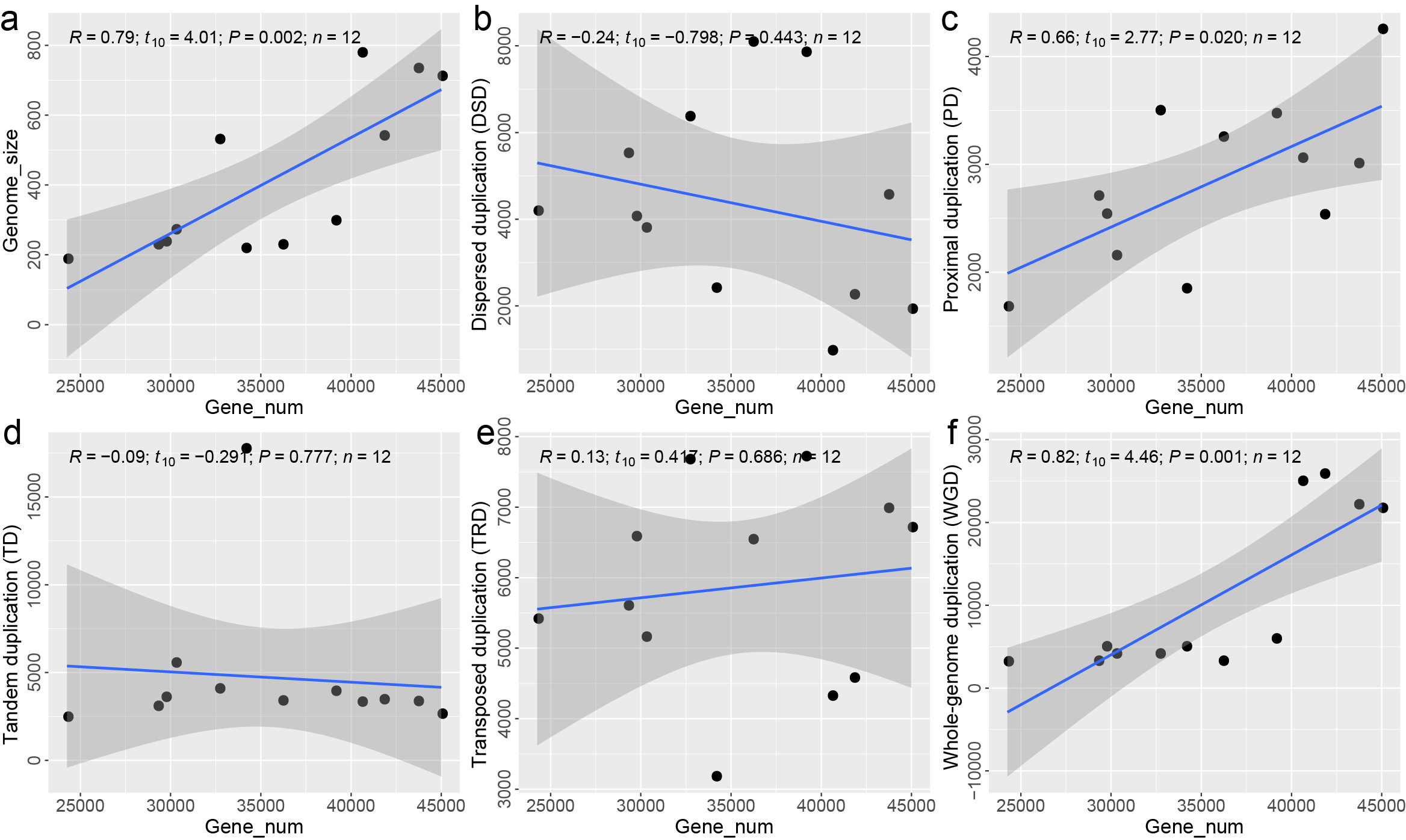


**Figure S6. Correlation between gene number and genome size, as well as the number of genes derived from different duplication modes across genomes.** Scatter plots depict the correlations between total gene number and (a) genome size, and the number of genes identified as arising from (b) dispersed duplication (DSD), (c) proximal duplication (PD), (d) tandem duplication (TD), (e) transposed duplication (TRD), and (f) whole-genome duplication (WGD). Each dot represents one species. Blue lines indicate linear regression fits, and shaded areas represent 95% confidence intervals. Pearson correlation coefficients (R), t-statistics, P-values, and sample sizes (n) are shown in each panel. Significant positive correlations were observed for genome size, PD and WGD, suggesting their potential contributions to gene number expansion.


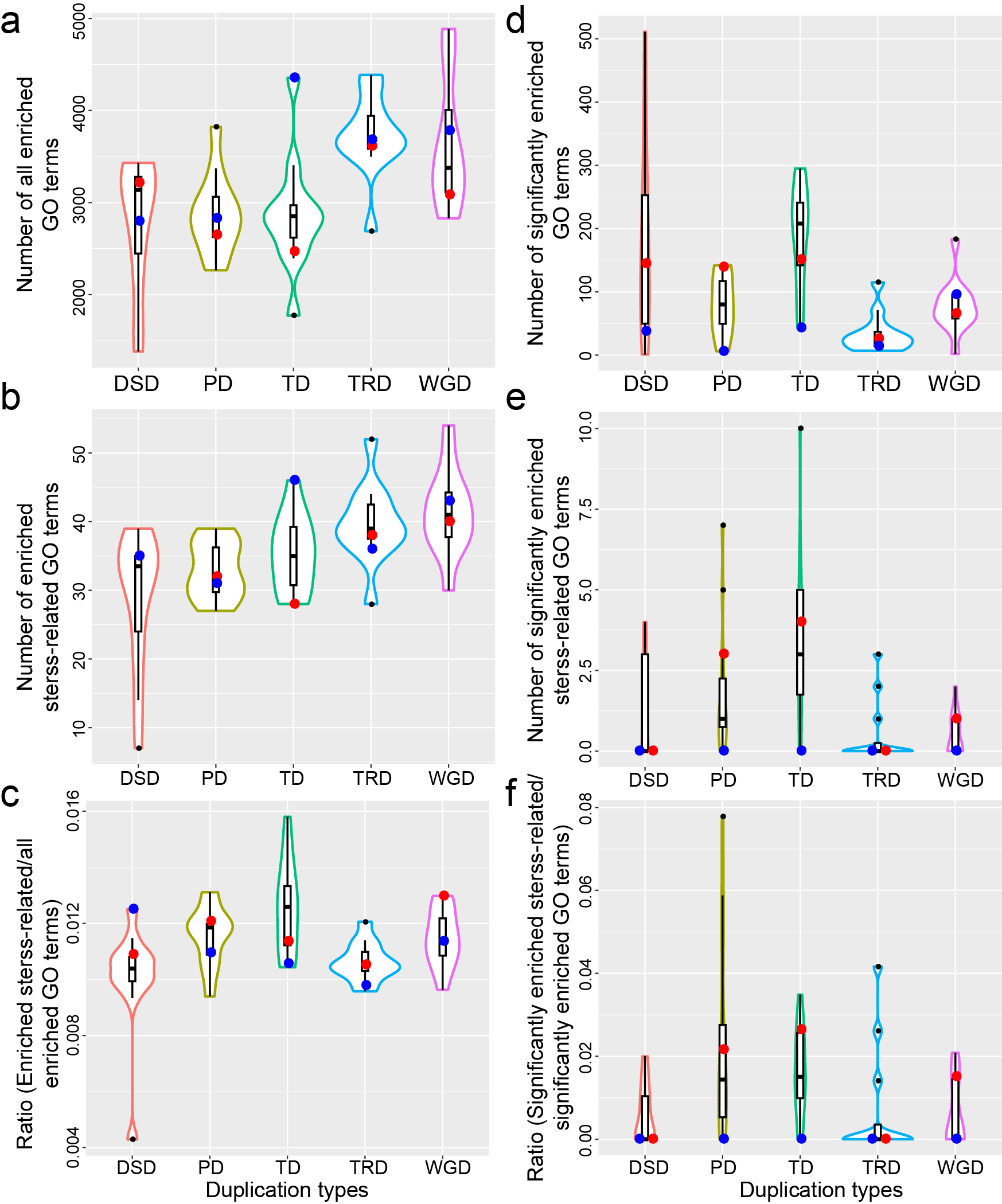


**Figure S7. Duplication-mode–dependent functional enrichment patterns of genes derived from whole-genome duplication (WGD) and small-scale duplications (SSDs) across Rosaceae species. (a)** Boxplots showing the total number of enriched Gene Ontology (GO) terms associated with genes derived from different duplication modes, including WGD and SSDs (dispersed duplication [DSD], proximal duplication [PD], tandem duplication [TD], and transposed duplication [TRD]), across Rosaceae species. **(b)** Boxplots showing the number of enriched stress-related GO terms for genes derived from different duplication modes across Rosaceae species. **(c)** Ratios of enriched stress-related GO terms to the total number of enriched GO terms for each duplication mode, calculated based on the data shown in panels (b) and (a), respectively. **(d)** Boxplots showing the number of significantly enriched GO terms associated with genes derived from different duplication modes across Rosaceae species. **(e)** Boxplots showing the number of significantly enriched stress-related GO terms for genes derived from different duplication modes across Rosaceae species. **(f)** Ratios of significantly enriched stress-related GO terms to the total number of significantly enriched GO terms for each duplication mode, calculated based on the data shown in panels (e) and (d), respectively. Each box represents the interquartile range, with the central line indicating the median value. Red and blue dots denote *S. chinensis* and *S. crenata*, respectively, shown in the context of the overall Rosaceae distribution.

**
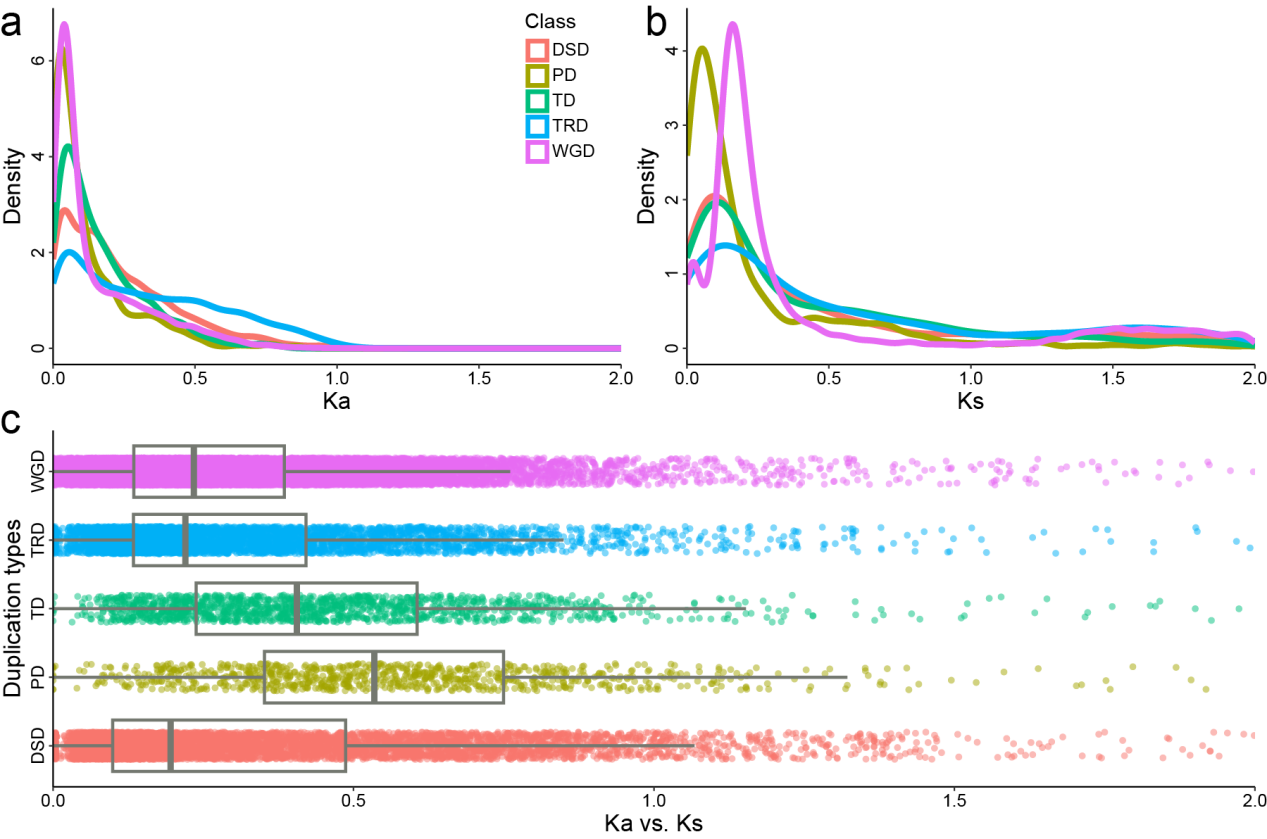
**

**Figure S8. Genome-wide evolutionary rate patterns of duplicated genes in *Pyrus pyrifolia*. (a)** Kernel density distributions of nonsynonymous substitution rates (Ka) and **(b)** synonymous substitution rates (Ks) for duplicated genes across the *S. chinensis* genome, stratified by duplication type, including dispersed duplication (DSD), proximal duplication (PD), tandem duplication (TD), transposed duplication (TRD), and whole-genome duplication (WGD). **(c)** Boxplot comparison of Ka/Ks ratios among different duplication types at the genome-wide scale. Horizontal lines within boxes indicate median values, and boxes represent the interquartile range.

**
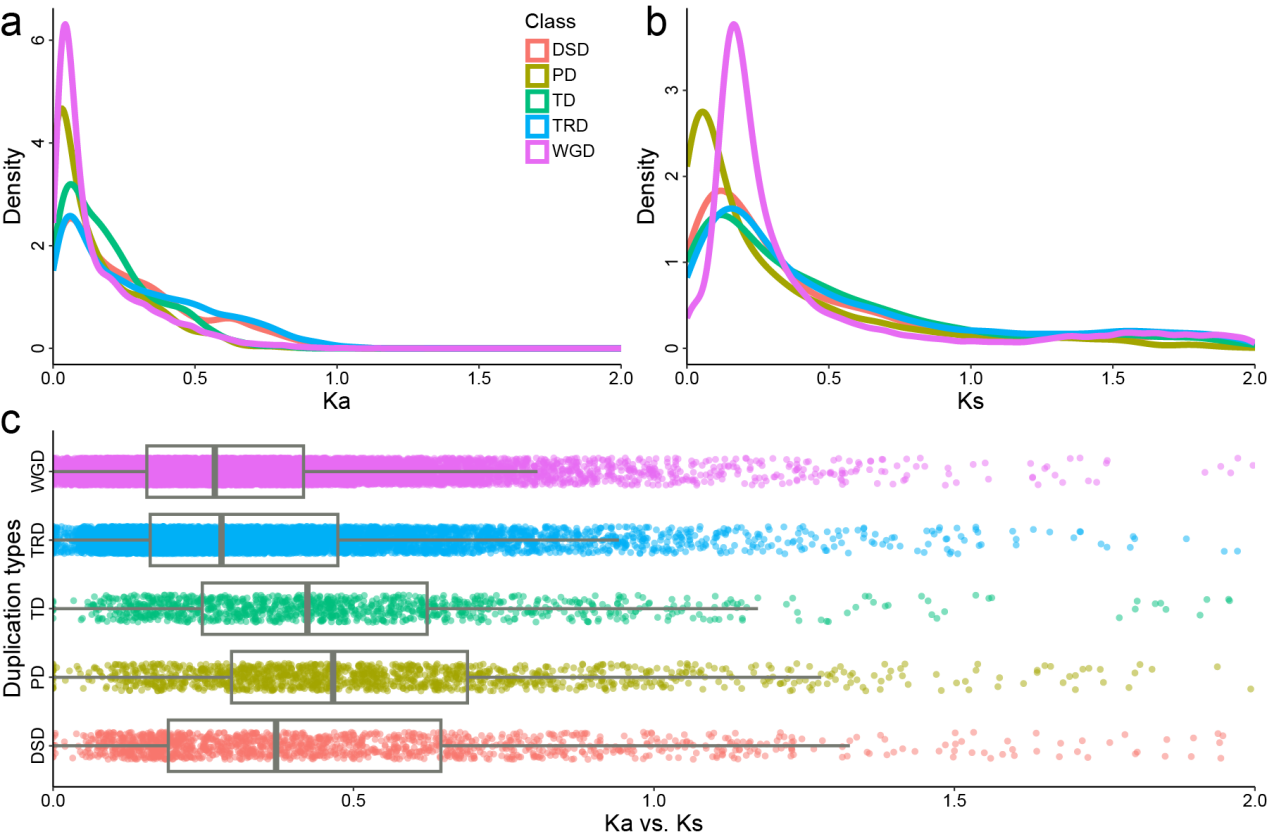
**

**Figure S9. Genome-wide evolutionary rate patterns of duplicated genes in *Malus prunifolia*. (a)** Kernel density distributions of nonsynonymous substitution rates (Ka) and **(b)** synonymous substitution rates (Ks) for duplicated genes across the *S. chinensis* genome, stratified by duplication type, including dispersed duplication (DSD), proximal duplication (PD), tandem duplication (TD), transposed duplication (TRD), and whole-genome duplication (WGD). **(c)** Boxplot comparison of Ka/Ks ratios among different duplication types at the genome-wide scale. Horizontal lines within boxes indicate median values, and boxes represent the interquartile range.


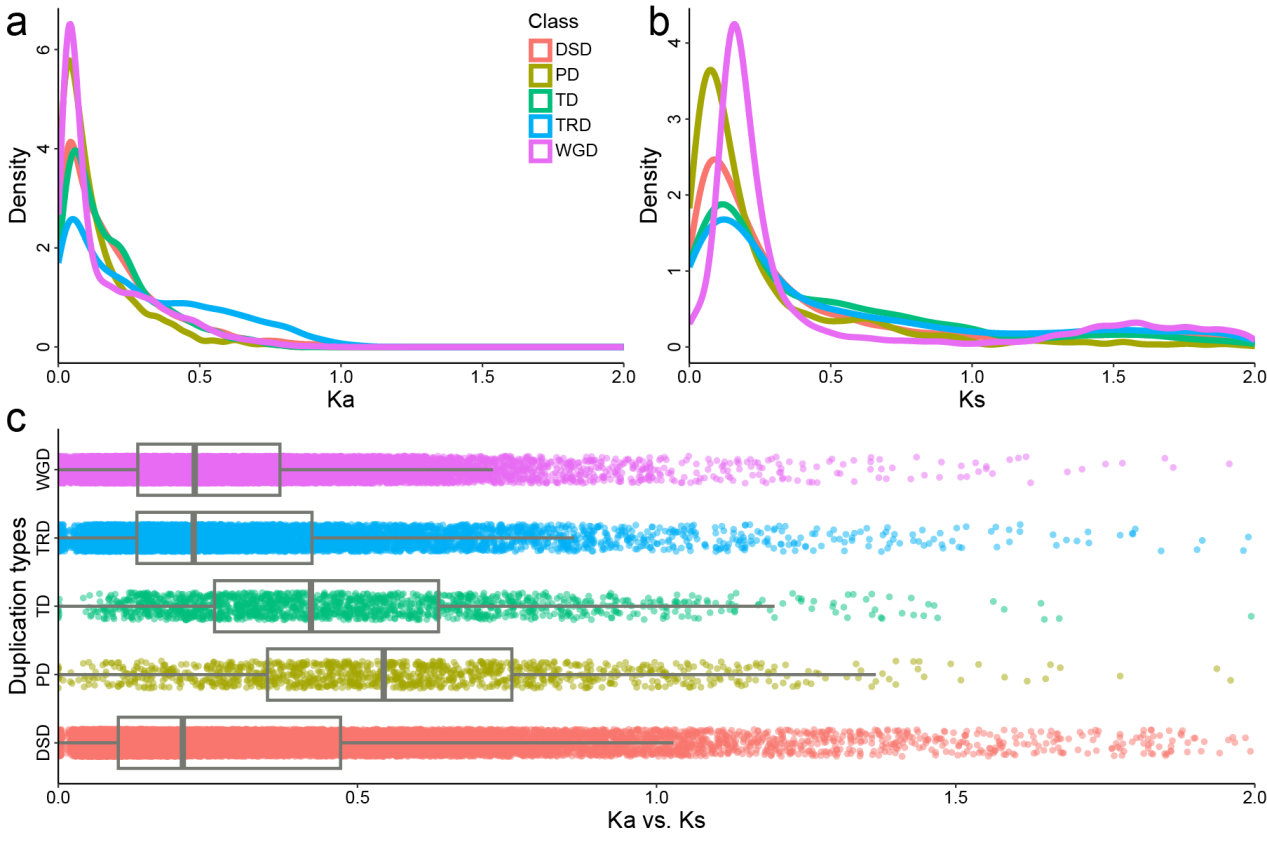


**Figure S10. Genome-wide evolutionary rate patterns of duplicated genes in *Eriobotrya japonica*. (a)** Kernel density distributions of nonsynonymous substitution rates (Ka) and **(b)** synonymous substitution rates (Ks) for duplicated genes across the *S. chinensis* genome, stratified by duplication type, including dispersed duplication (DSD), proximal duplication (PD), tandem duplication (TD), transposed duplication (TRD), and whole-genome duplication (WGD). **(c)** Boxplot comparison of Ka/Ks ratios among different duplication types at the genome-wide scale. Horizontal lines within boxes indicate median values, and boxes represent the interquartile range.


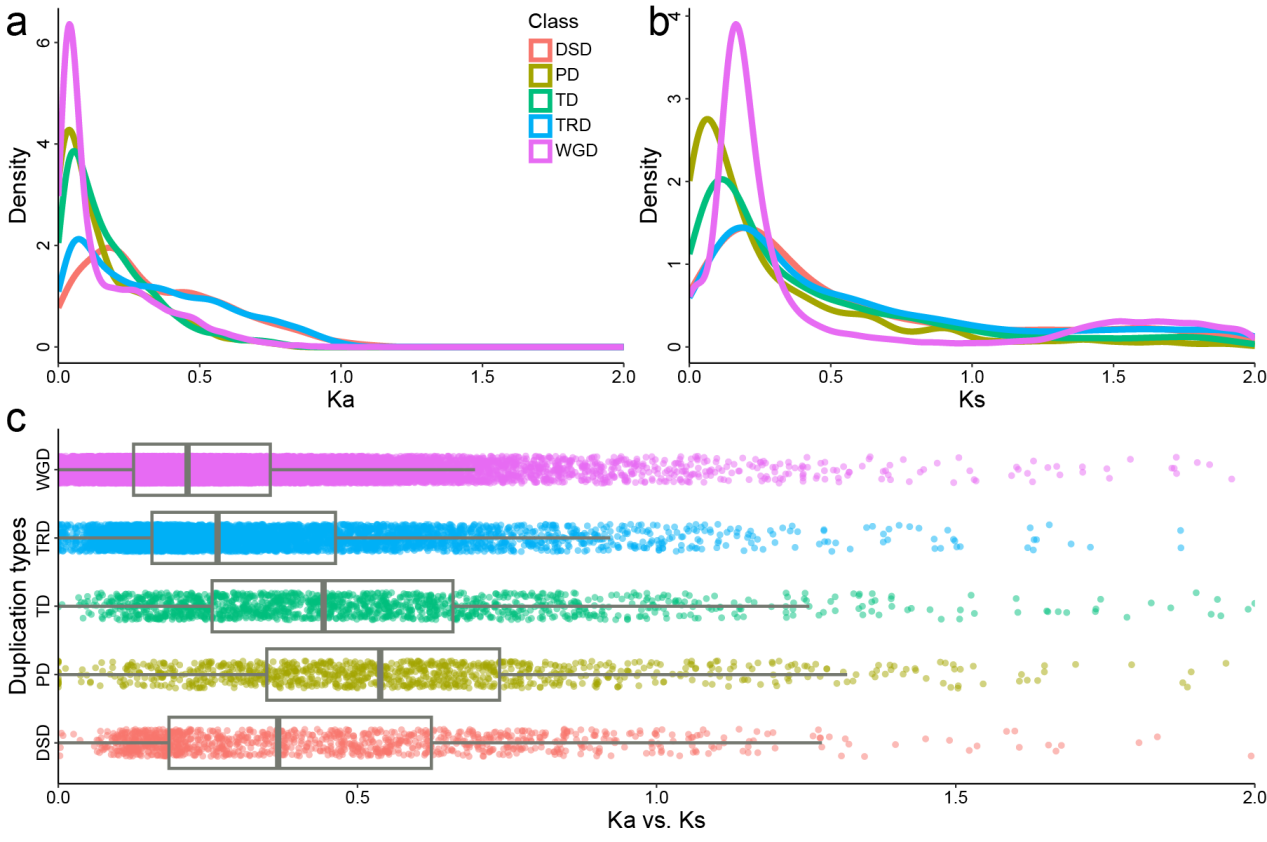


**Figure S11. Genome-wide evolutionary rate patterns of duplicated genes in *Crataegus pinnatifida* var. *major*. (a)** Kernel density distributions of nonsynonymous substitution rates (Ka) and **(b)** synonymous substitution rates (Ks) for duplicated genes across the *S. chinensis* genome, stratified by duplication type, including dispersed duplication (DSD), proximal duplication (PD), tandem duplication (TD), transposed duplication (TRD), and whole-genome duplication (WGD). **(c)** Boxplot comparison of Ka/Ks ratios among different duplication types at the genome-wide scale. Horizontal lines within boxes indicate median values, and boxes represent the interquartile range.


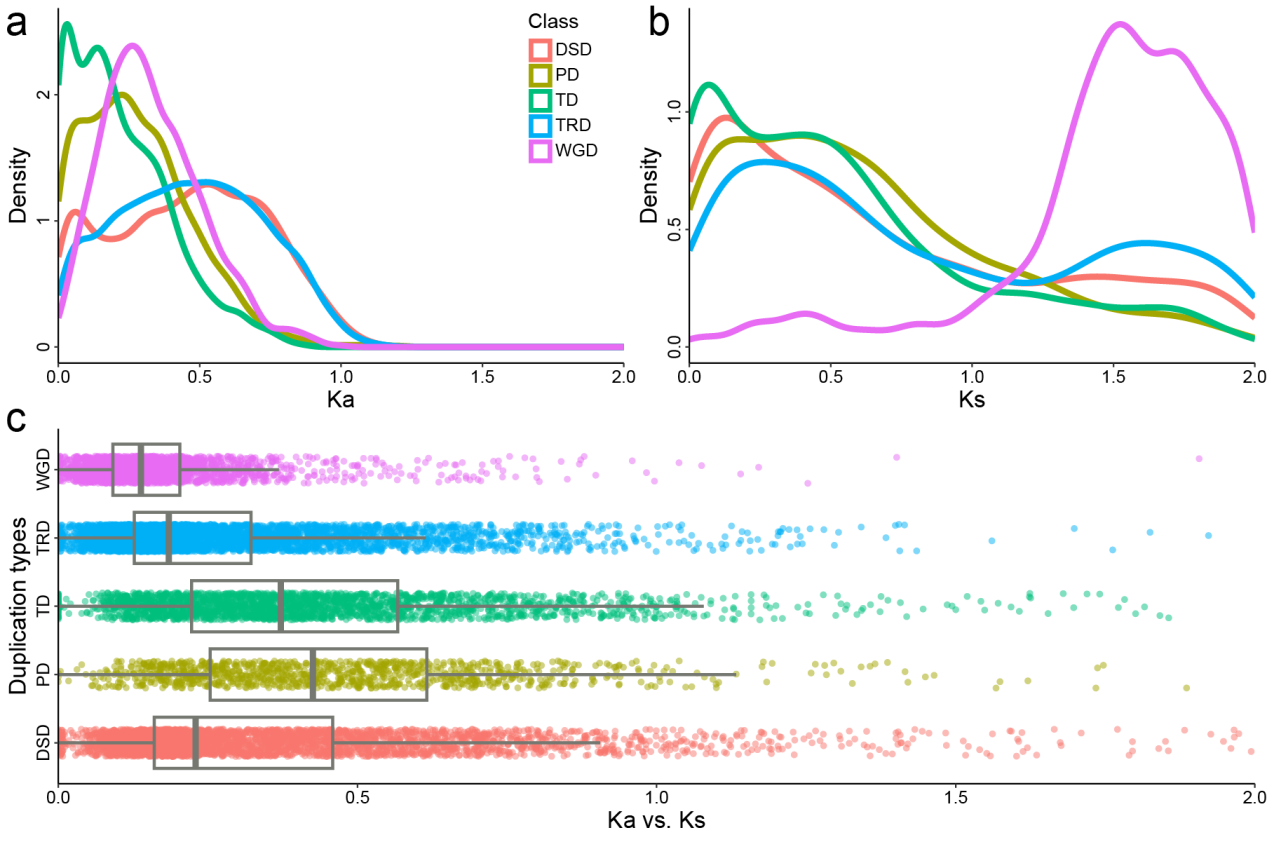


**Figure S12. Genome-wide evolutionary rate patterns of duplicated genes in *Gillenia trifoliata*. (a)** Kernel density distributions of nonsynonymous substitution rates (Ka) and **(b)** synonymous substitution rates (Ks) for duplicated genes across the *S. chinensis* genome, stratified by duplication type, including dispersed duplication (DSD), proximal duplication (PD), tandem duplication (TD), transposed duplication (TRD), and whole-genome duplication (WGD). **(c)** Boxplot comparison of Ka/Ks ratios among different duplication types at the genome-wide scale. Horizontal lines within boxes indicate median values, and boxes represent the interquartile range.


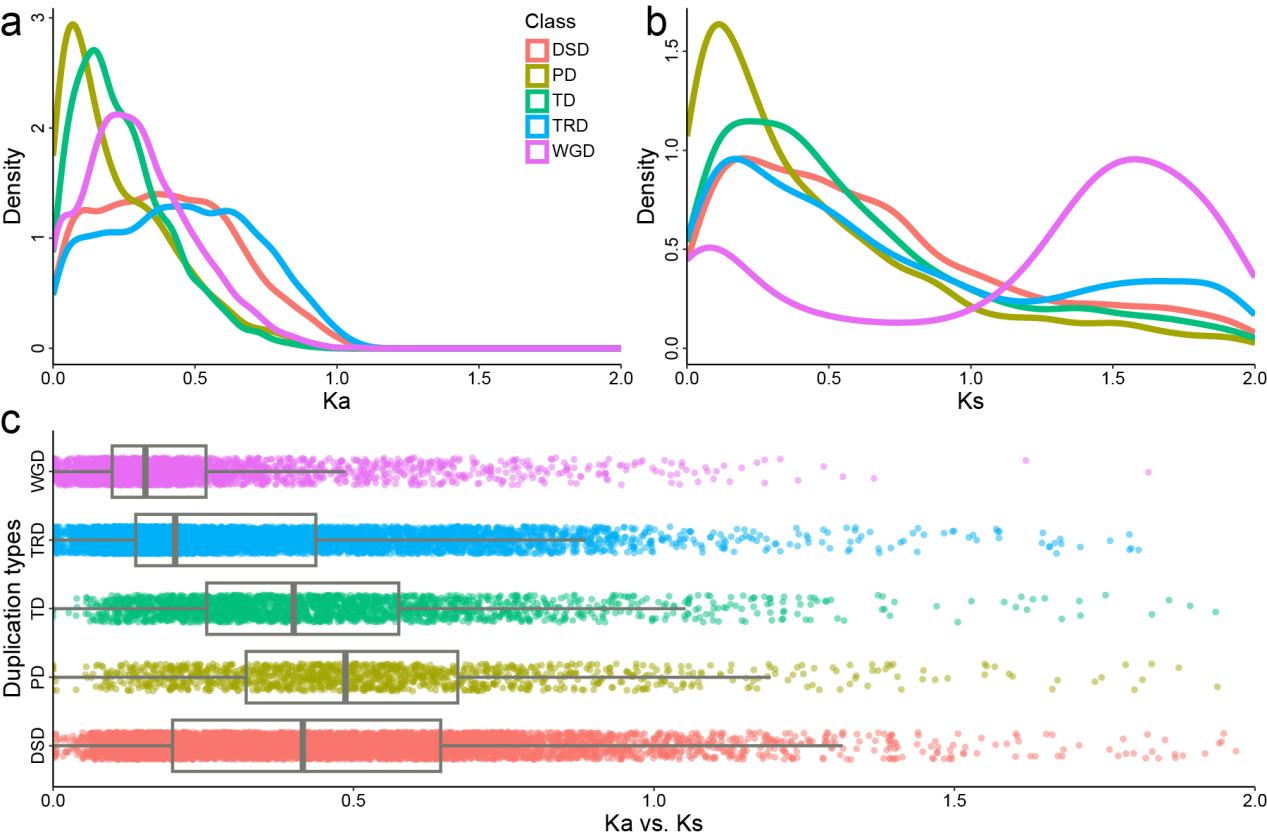


**Figure S13. Genome-wide evolutionary rate patterns of duplicated genes in *Prunus mume*. (a)** Kernel density distributions of nonsynonymous substitution rates (Ka) and **(b)** synonymous substitution rates (Ks) for duplicated genes across the *S. chinensis* genome, stratified by duplication type, including dispersed duplication (DSD), proximal duplication (PD), tandem duplication (TD), transposed duplication (TRD), and whole-genome duplication (WGD). **(c)** Boxplot comparison of Ka/Ks ratios among different duplication types at the genome-wide scale. Horizontal lines within boxes indicate median values, and boxes represent the interquartile range.


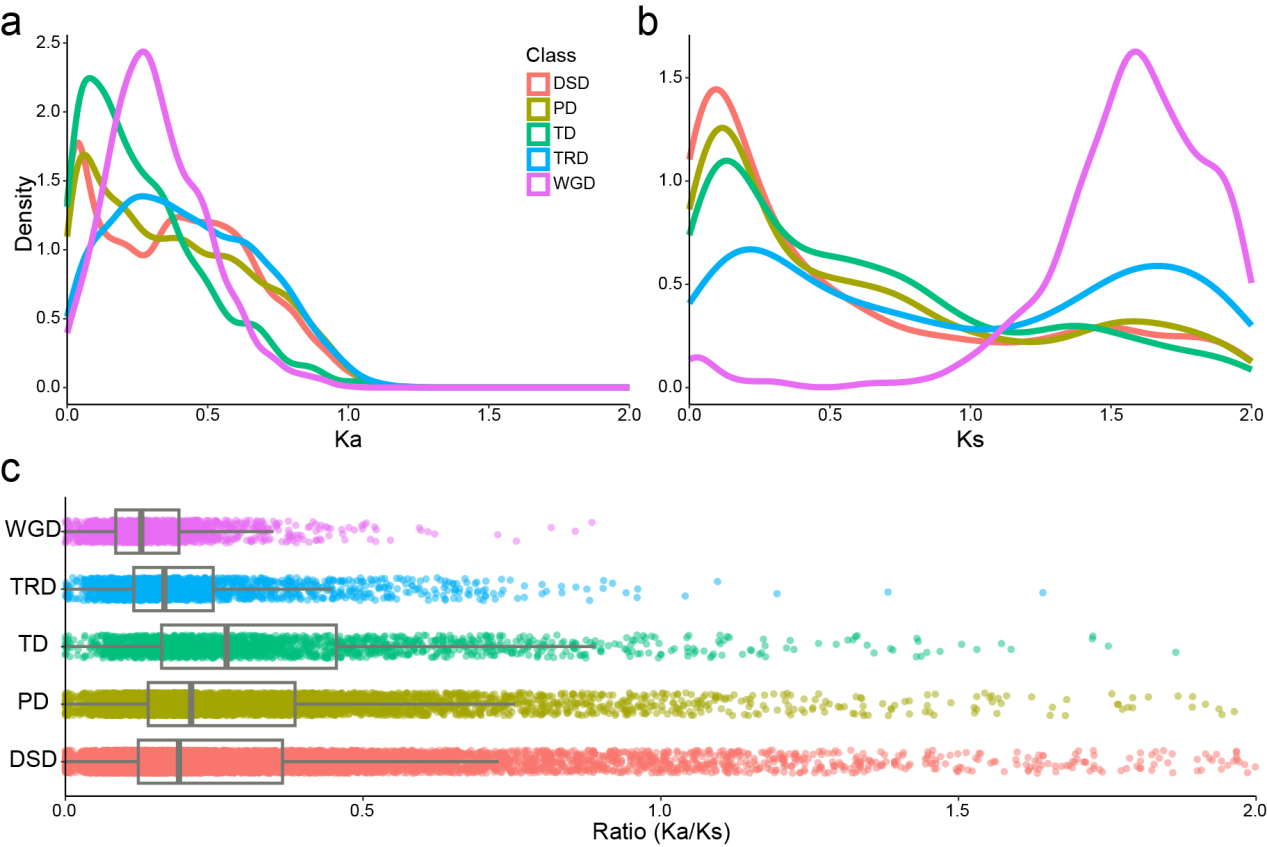


**Figure S14. Genome-wide evolutionary rate patterns of duplicated genes in *S. chinensis*. (a)** Kernel density distributions of nonsynonymous substitution rates (Ka) and **(b)** synonymous substitution rates (Ks) for duplicated genes across the *S. chinensis* genome, stratified by duplication type, including dispersed duplication (DSD), proximal duplication (PD), tandem duplication (TD), transposed duplication (TRD), and whole-genome duplication (WGD). **(c)** Boxplot comparison of Ka/Ks ratios among different duplication types at the genome-wide scale. Horizontal lines within boxes indicate median values, and boxes represent the interquartile range.

**
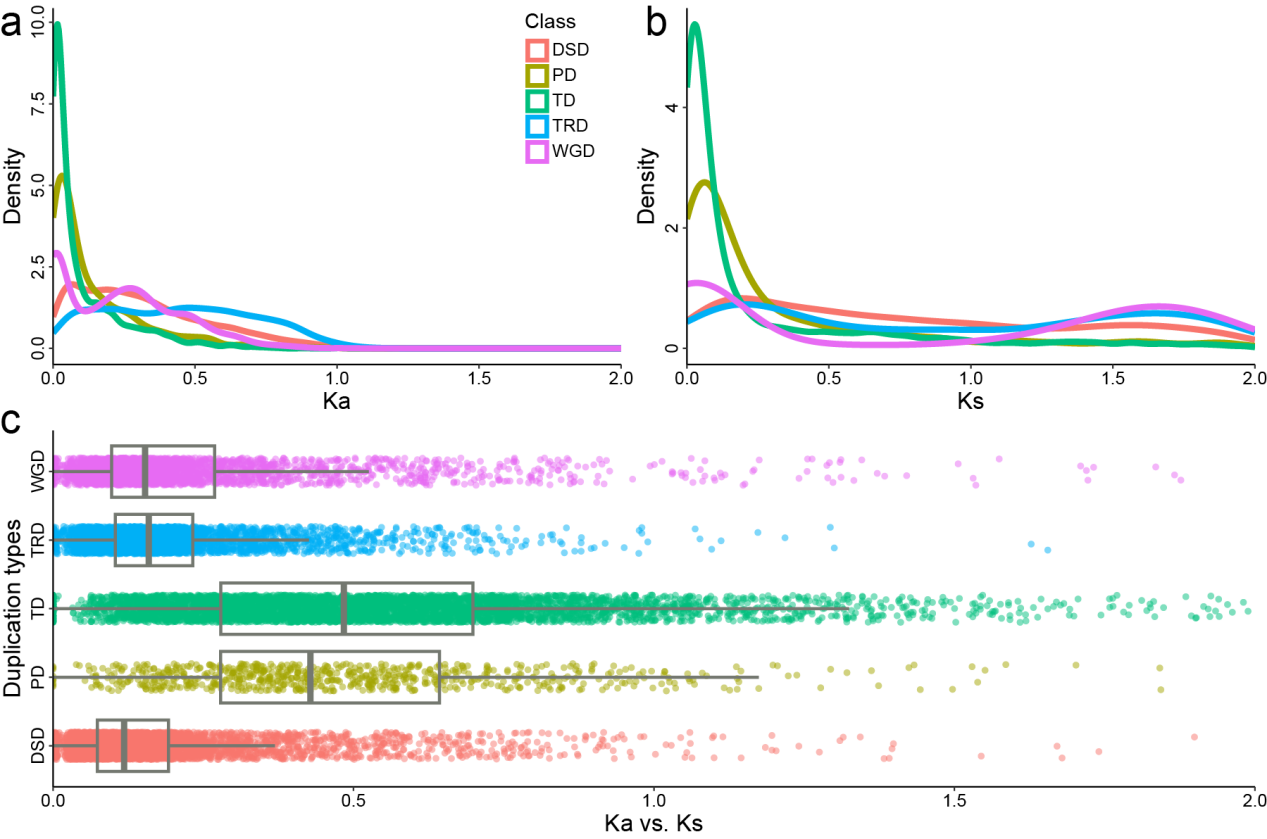
**

**Figure S15. Genome-wide evolutionary rate patterns of duplicated genes in *Spiraea crenata*. (a)** Kernel density distributions of nonsynonymous substitution rates (Ka) and **(b)** synonymous substitution rates (Ks) for duplicated genes across the *S. chinensis* genome, stratified by duplication type, including dispersed duplication (DSD), proximal duplication (PD), tandem duplication (TD), transposed duplication (TRD), and whole-genome duplication (WGD). **(c)** Boxplot comparison of Ka/Ks ratios among different duplication types at the genome-wide scale. Horizontal lines within boxes indicate median values, and boxes represent the interquartile range.

**
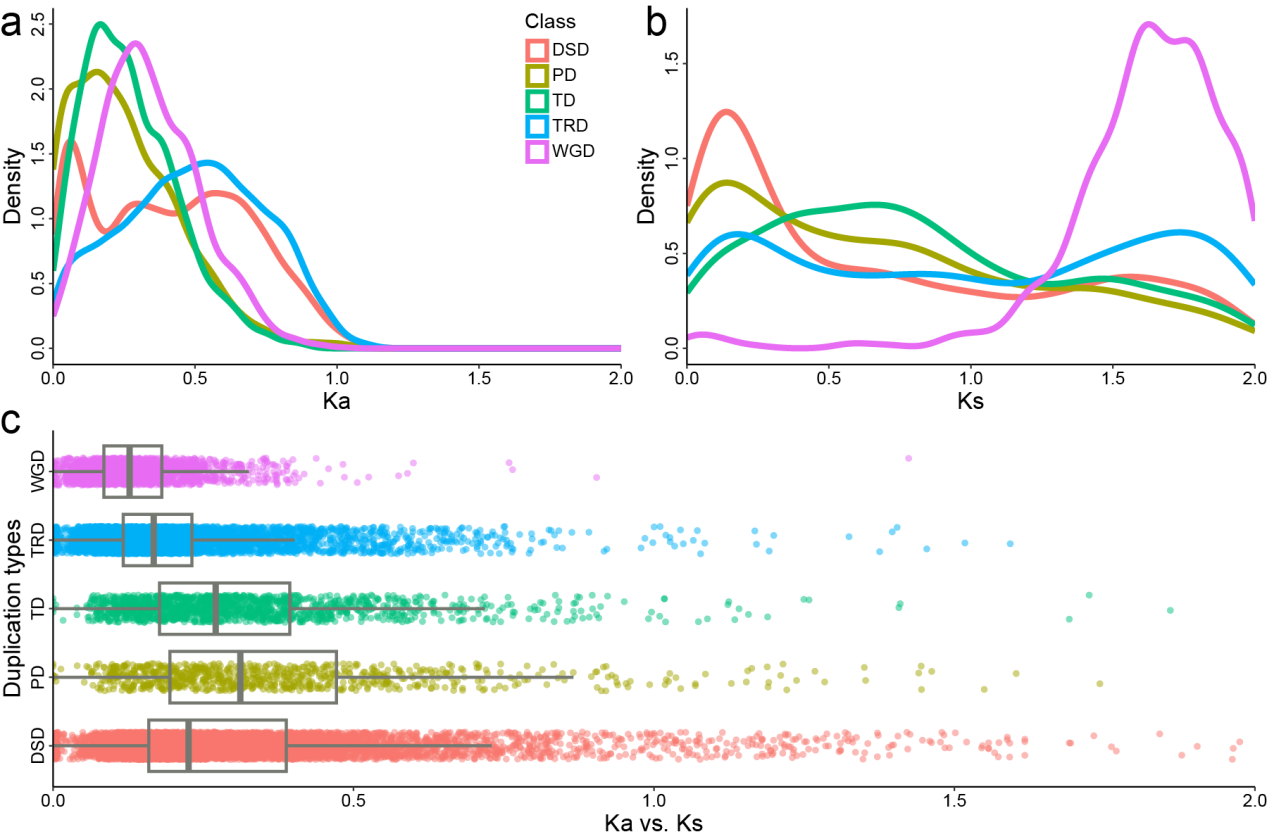
**

**Figure S16. Genome-wide evolutionary rate patterns of duplicated genes in *Argentina lineata*. (a)** Kernel density distributions of nonsynonymous substitution rates (Ka) and **(b)** synonymous substitution rates (Ks) for duplicated genes across the *S. chinensis* genome, stratified by duplication type, including dispersed duplication (DSD), proximal duplication (PD), tandem duplication (TD), transposed duplication (TRD), and whole-genome duplication (WGD). **(c)** Boxplot comparison of Ka/Ks ratios among different duplication types at the genome-wide scale. Horizontal lines within boxes indicate median values, and boxes represent the interquartile range.

**
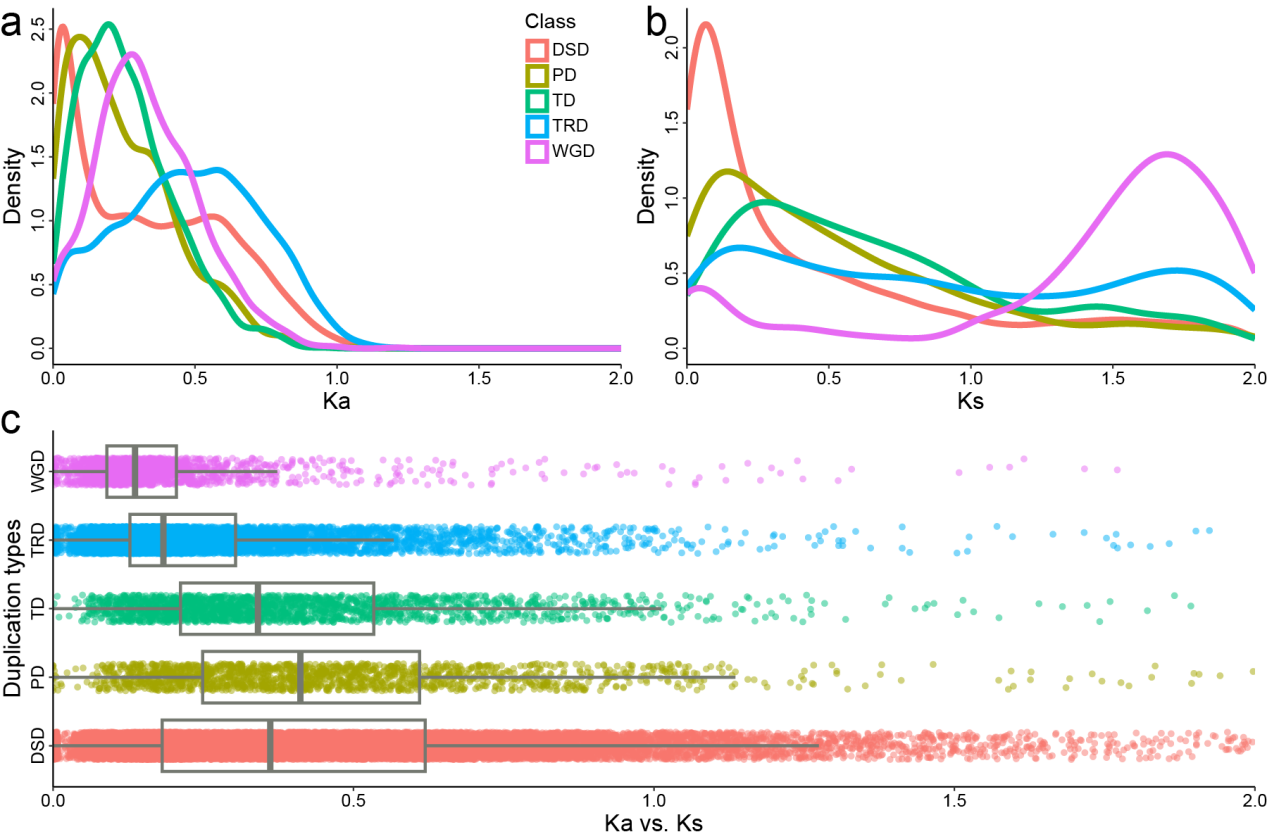
**

**Figure S17. Genome-wide evolutionary rate patterns of duplicated genes in *Fragaria vesca*. (a)** Kernel density distributions of nonsynonymous substitution rates (Ka) and **(b)** synonymous substitution rates (Ks) for duplicated genes across the *S. chinensis* genome, stratified by duplication type, including dispersed duplication (DSD), proximal duplication (PD), tandem duplication (TD), transposed duplication (TRD), and whole-genome duplication (WGD). **(c)** Boxplot comparison of Ka/Ks ratios among different duplication types at the genome-wide scale. Horizontal lines within boxes indicate median values, and boxes represent the interquartile range.

**
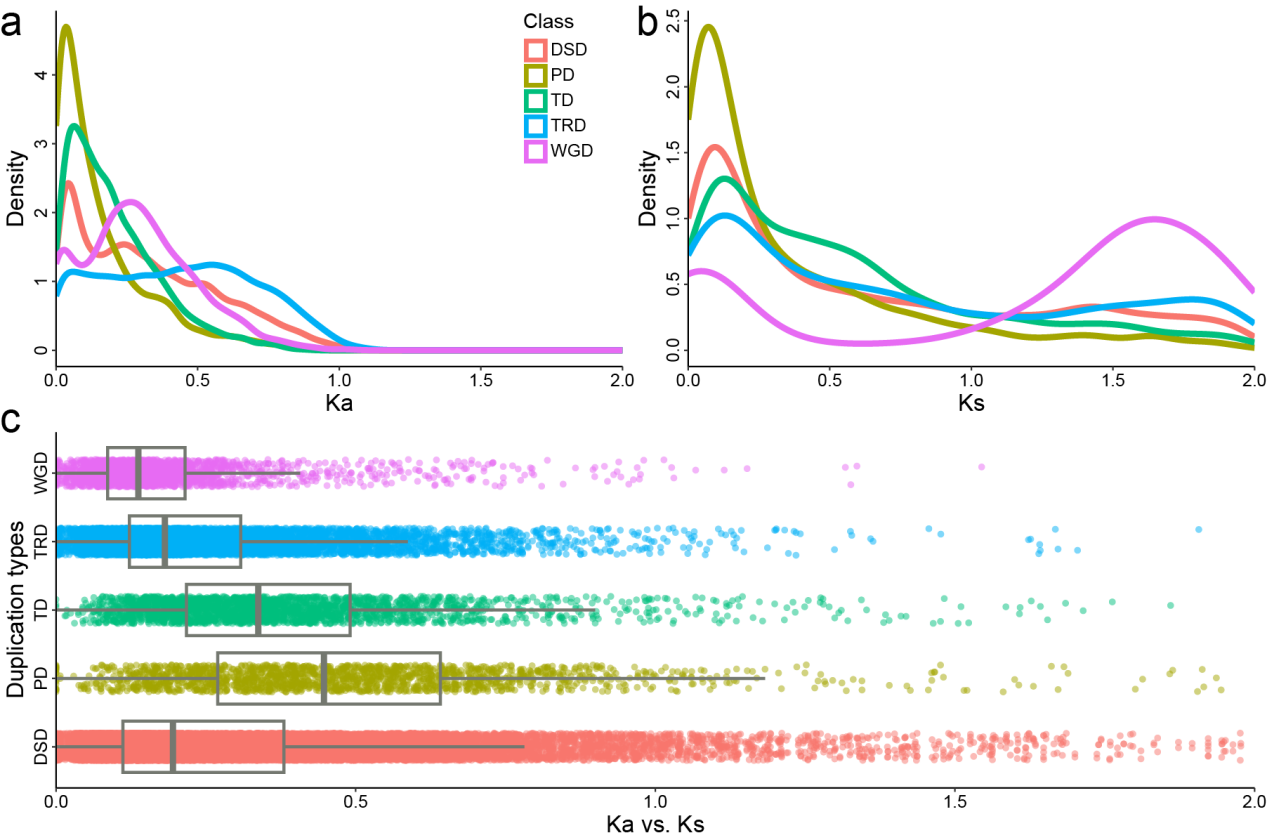
**

**Figure S18. Genome-wide evolutionary rate patterns of duplicated genes in *Rosa wichuraiana*. (a)** Kernel density distributions of nonsynonymous substitution rates (Ka) and **(b)** synonymous substitution rates (Ks) for duplicated genes across the *S. chinensis* genome, stratified by duplication type, including dispersed duplication (DSD), proximal duplication (PD), tandem duplication (TD), transposed duplication (TRD), and whole-genome duplication (WGD). **(c)** Boxplot comparison of Ka/Ks ratios among different duplication types at the genome-wide scale. Horizontal lines within boxes indicate median values, and boxes represent the interquartile range.

**
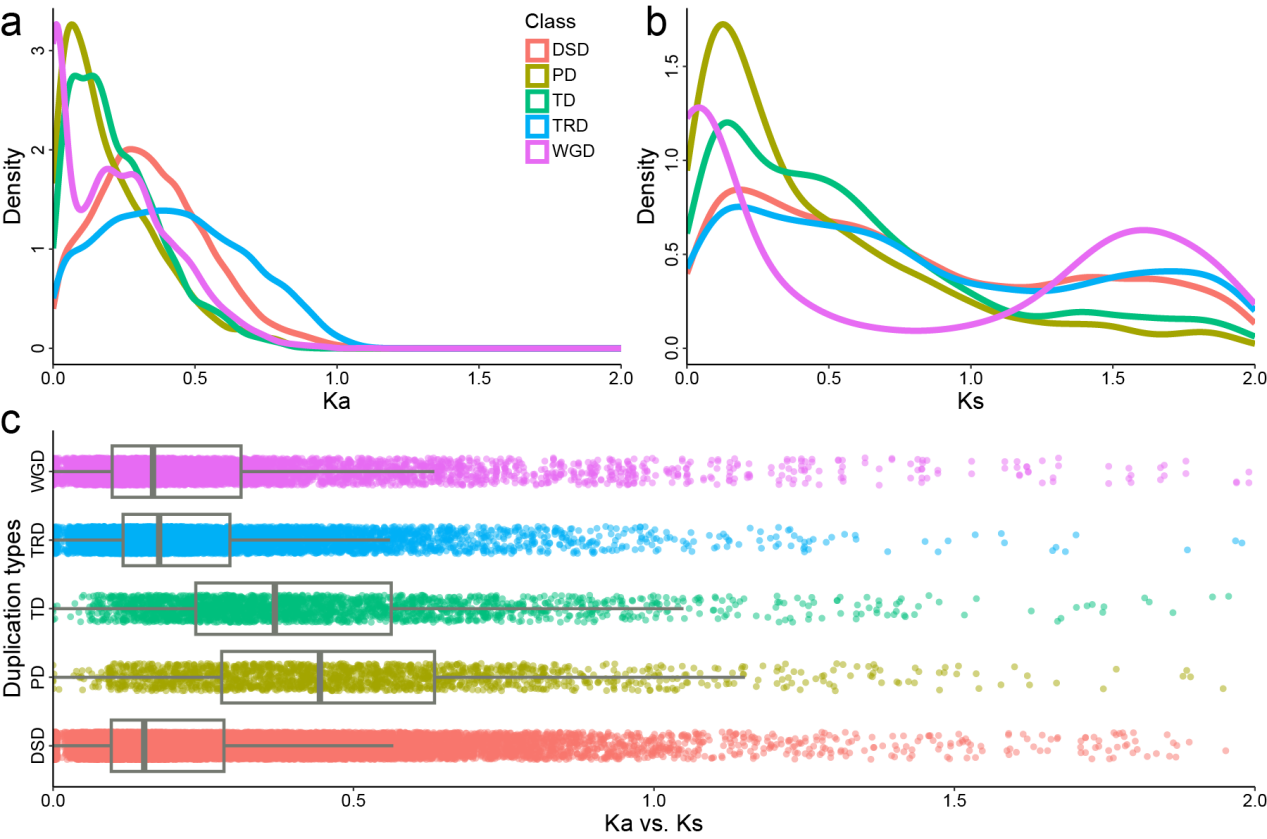
**

**Figure S19. Genome-wide evolutionary rate patterns of duplicated genes in *Rubus idaeus*. (a)** Kernel density distributions of nonsynonymous substitution rates (Ka) and **(b)** synonymous substitution rates (Ks) for duplicated genes across the *S. chinensis* genome, stratified by duplication type, including dispersed duplication (DSD), proximal duplication (PD), tandem duplication (TD), transposed duplication (TRD), and whole-genome duplication (WGD). **(c)** Boxplot comparison of Ka/Ks ratios among different duplication types at the genome-wide scale. Horizontal lines within boxes indicate median values, and boxes represent the interquartile range.


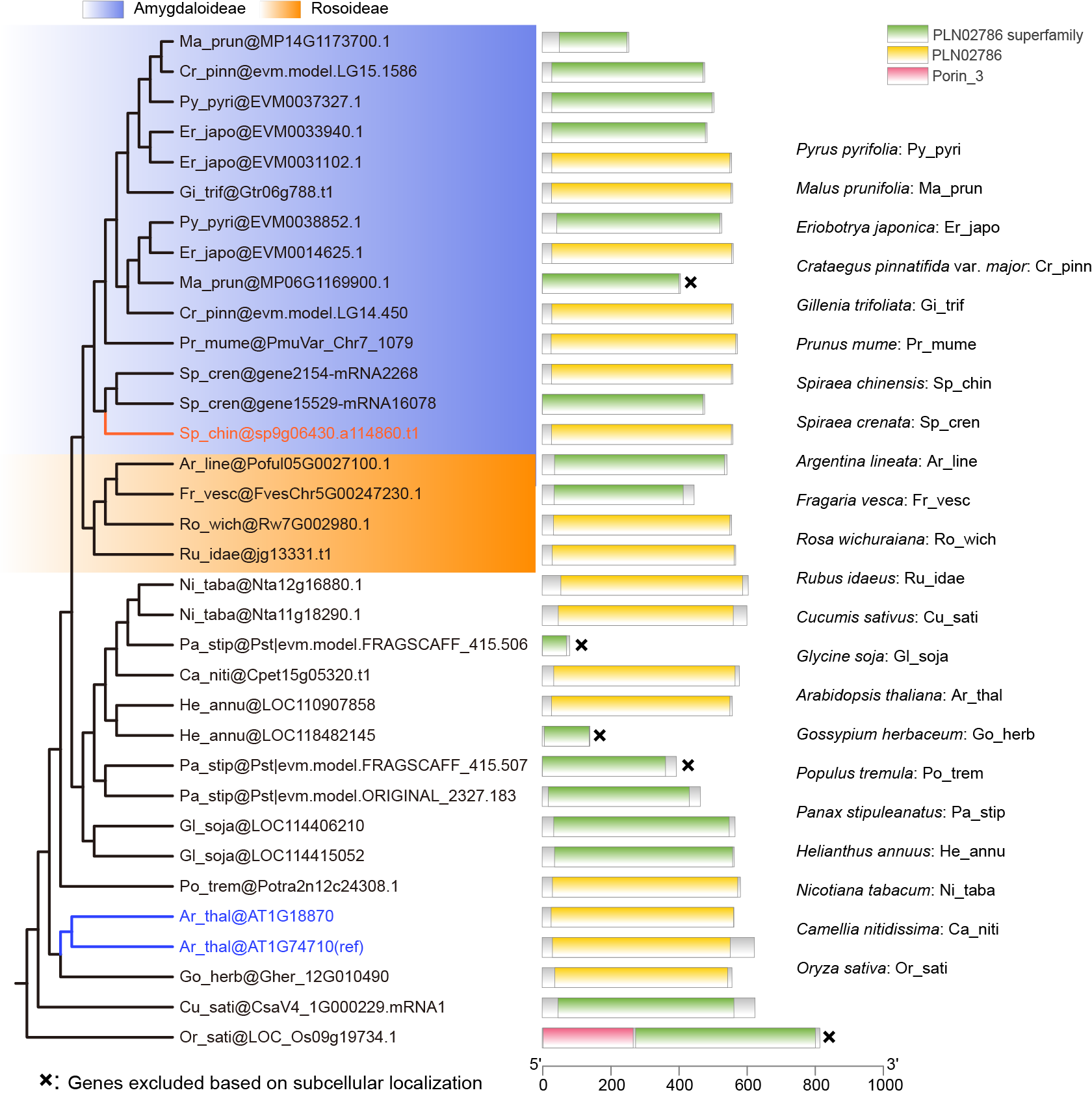


**Figure S20. Maximum likelihood phylogenetic tree of ICS1 gene copies involved in the ICS-mediated SA biosynthesis pathway across Rosaceae species and representative outgroups.** Clades are shaded by subfamily, with blue indicating Amygdaloideae and orange indicating Rosoideae. Gene copies and branches from S. chinensis and A. thaliana are specifically highlighted in orange and blue, respectively. Each gene is labeled with species abbreviation and gene ID (see **Tables S15 and S22** for details). Conserved domain architectures, annotated using the NCBI Conserved Domain Database (CDD; [https://www.ncbi.nlm.nih.gov/Structure/bwrpsb/bwrpsb.cgi](https://www.ncbi.nlm.nih.gov/Structure/bwrpsb/bwrpsb.cgi)), are displayed as bar plots to the right of the tree. Visualization was performed in TBtools-II. Black “×” symbols denote genes excluded on the basis of inconsistent subcellular localization, whereas red “×” symbols denote genes excluded as phylogenetic outliers.


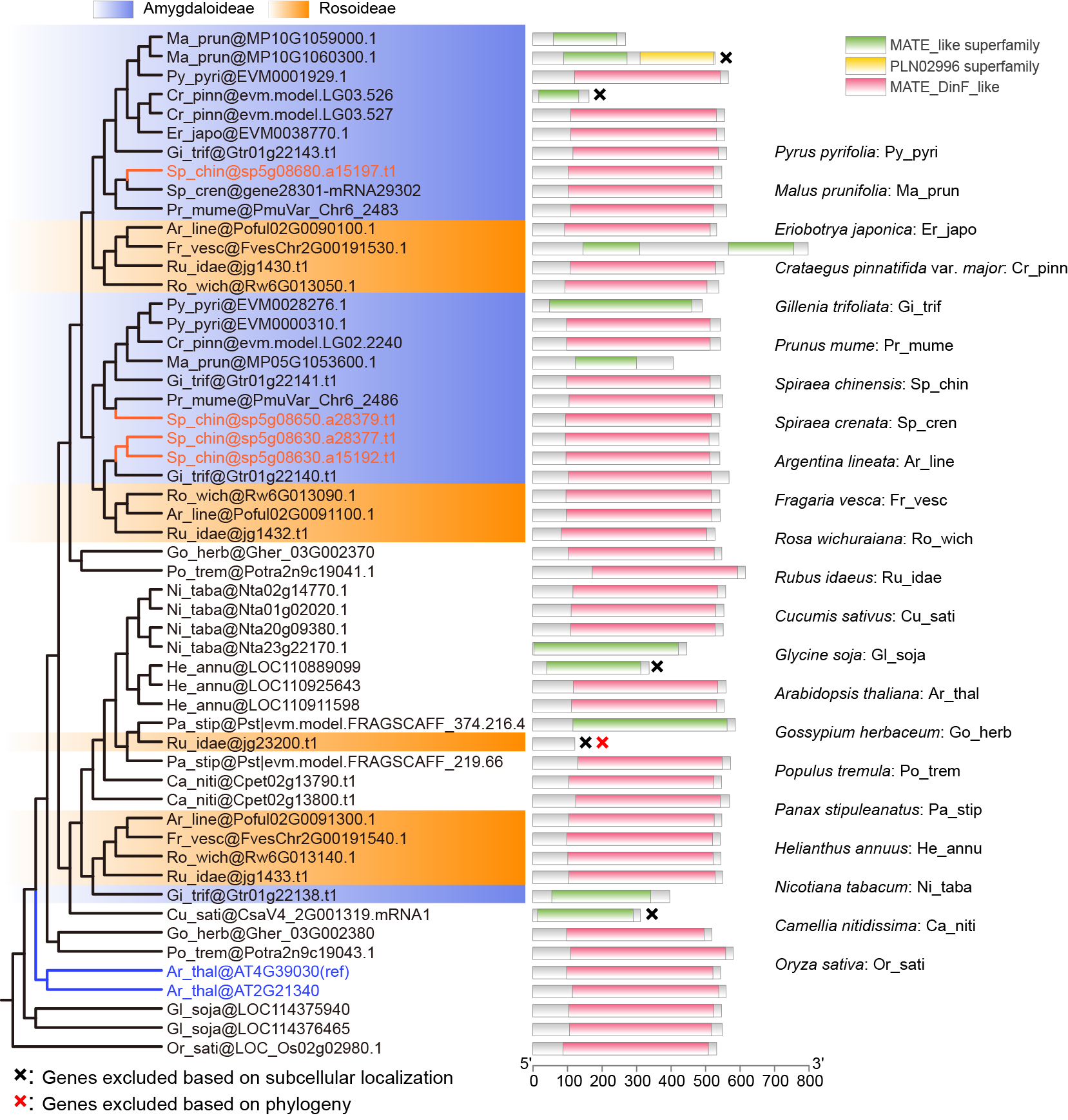


**Figure S21. Maximum likelihood phylogenetic tree of EDS5 gene copies involved in the ICS-mediated SA biosynthesis pathway across Rosaceae species and representative outgroups.** Annotation details are consistent with those shown in Fig. S20.


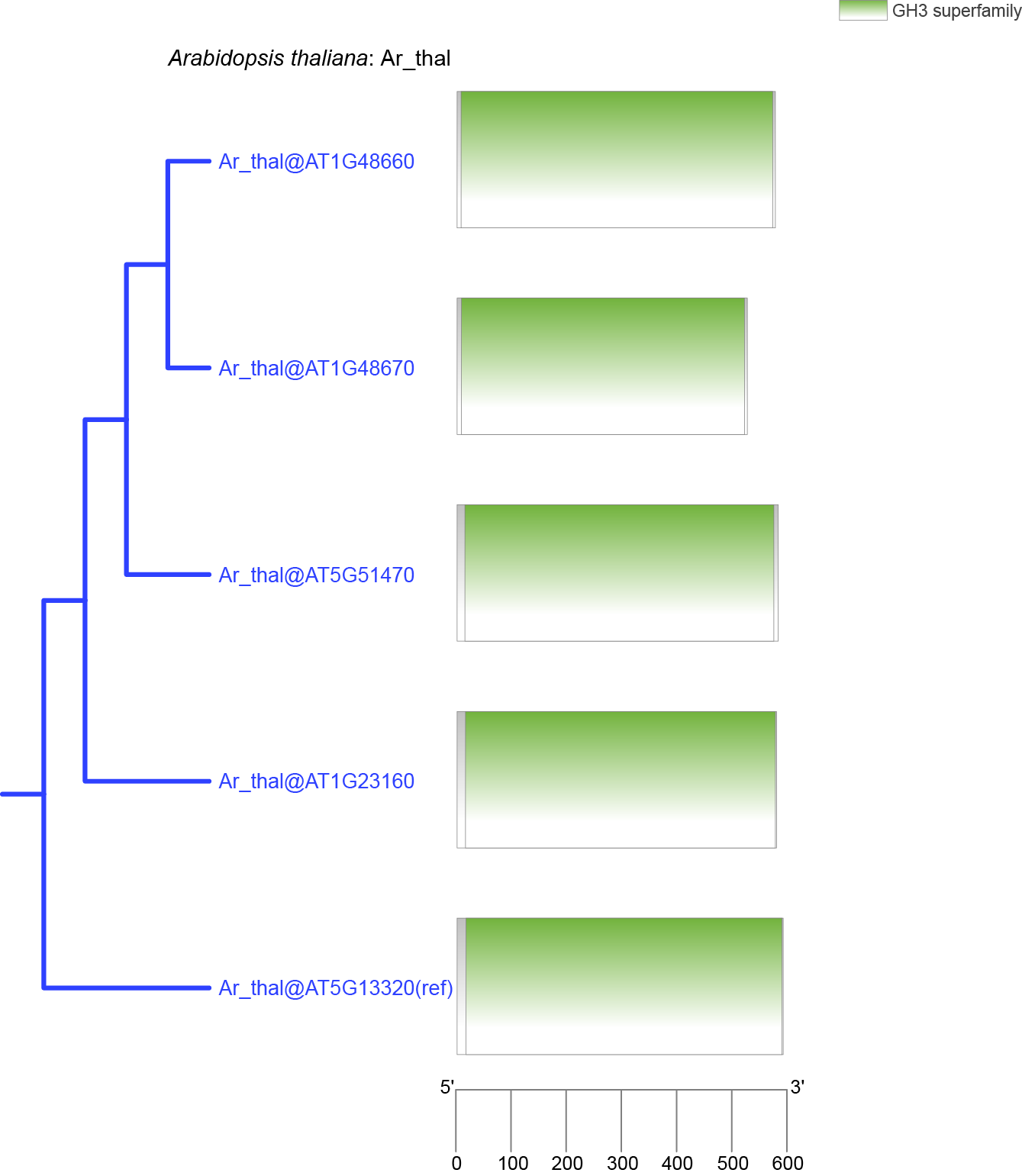


**Figure S22. Maximum likelihood phylogenetic tree of *PBS1* gene copies involved in the ICS-mediated SA biosynthesis pathway across Rosaceae species and representative outgroups.** Annotation details are consistent with those shown in Fig. S20.


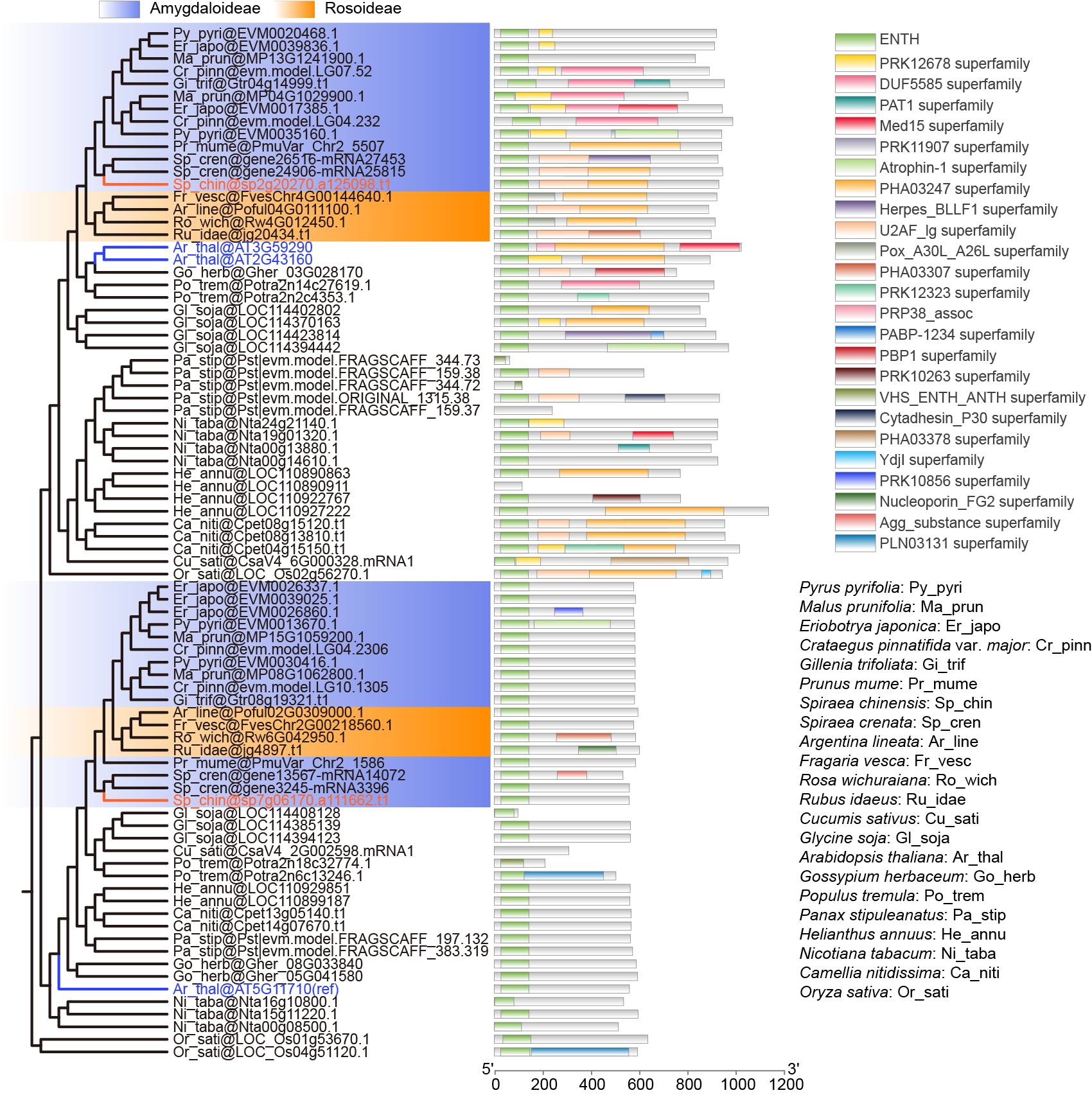


**Figure S23. Maximum likelihood phylogenetic tree of EPS1 gene copies involved in the ICS-mediated SA biosynthesis pathway across Rosaceae species and representative outgroups.** Annotation details are consistent with those shown in Fig. S20.


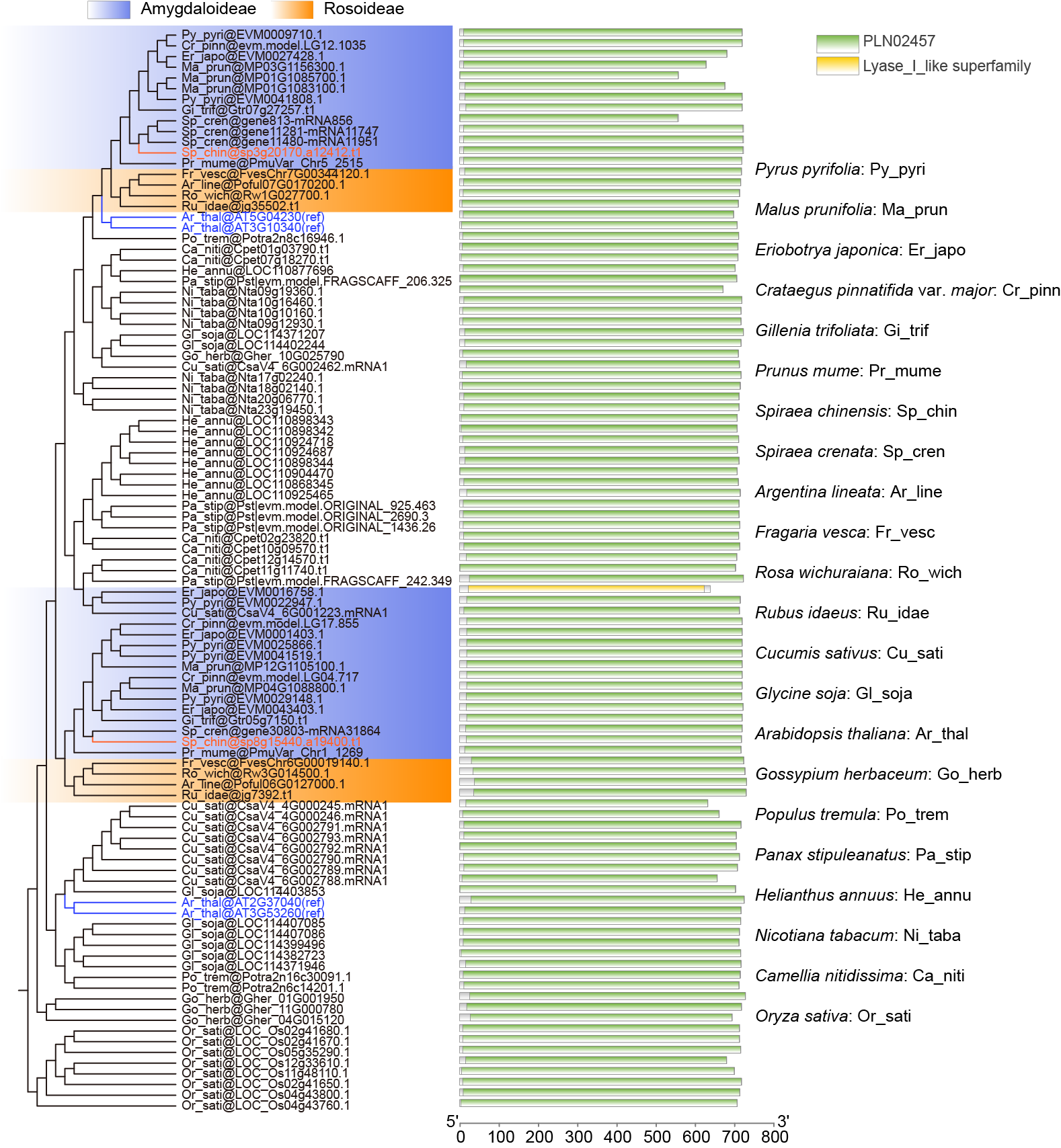


**Figure S24. Maximum likelihood phylogenetic tree of PAL gene copies involved in the PAL-mediated SA biosynthesis pathway across Rosaceae species and representative outgroups.** Annotation details are consistent with those shown in Fig. S20.


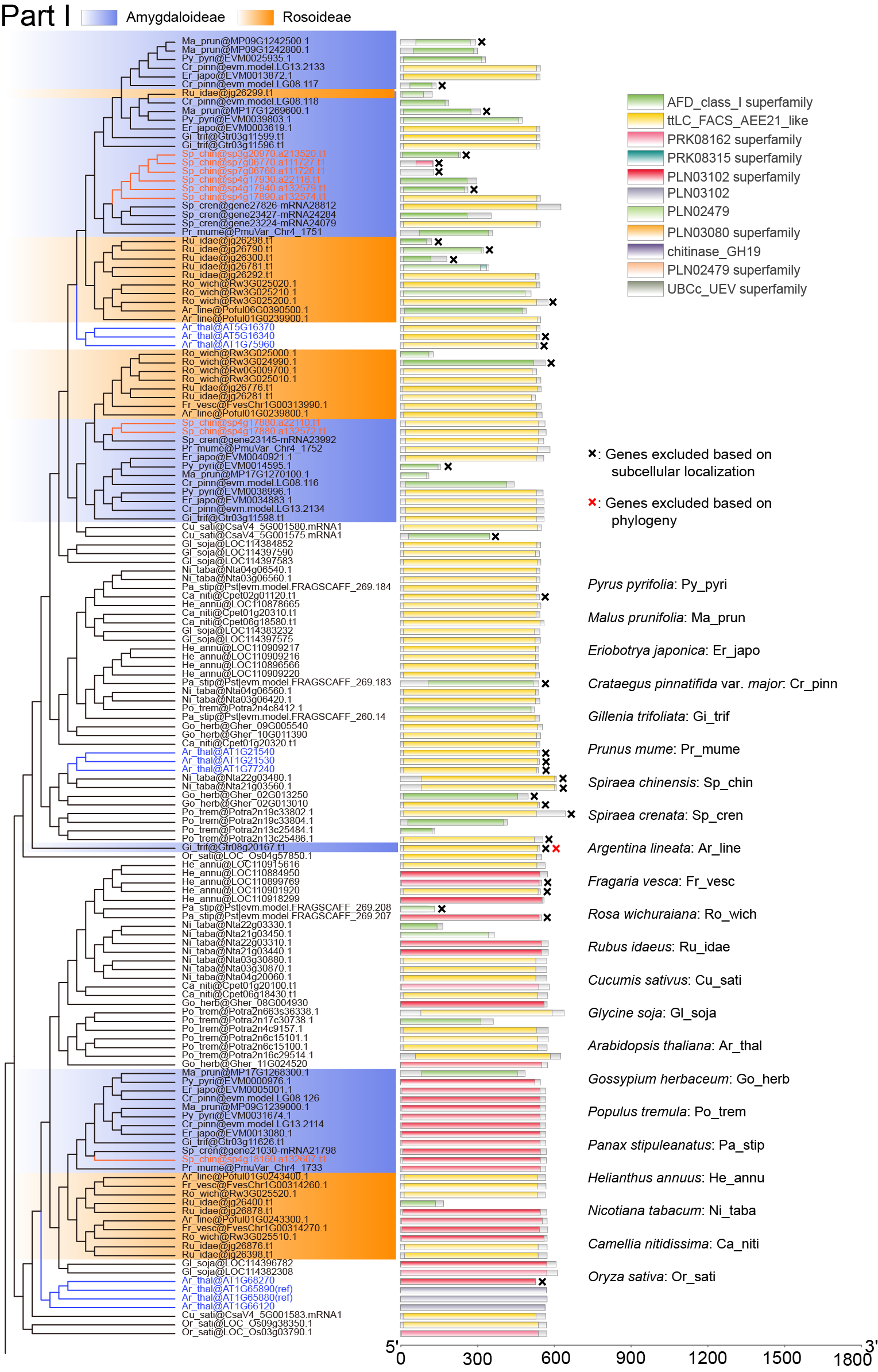


**Figure S25a. Maximum likelihood phylogenetic tree of CNL gene copies involved in the PAL-mediated SA biosynthesis pathway across Rosaceae species and representative outgroups (Part I).** Annotation details are consistent with those shown in Fig. S20.

**
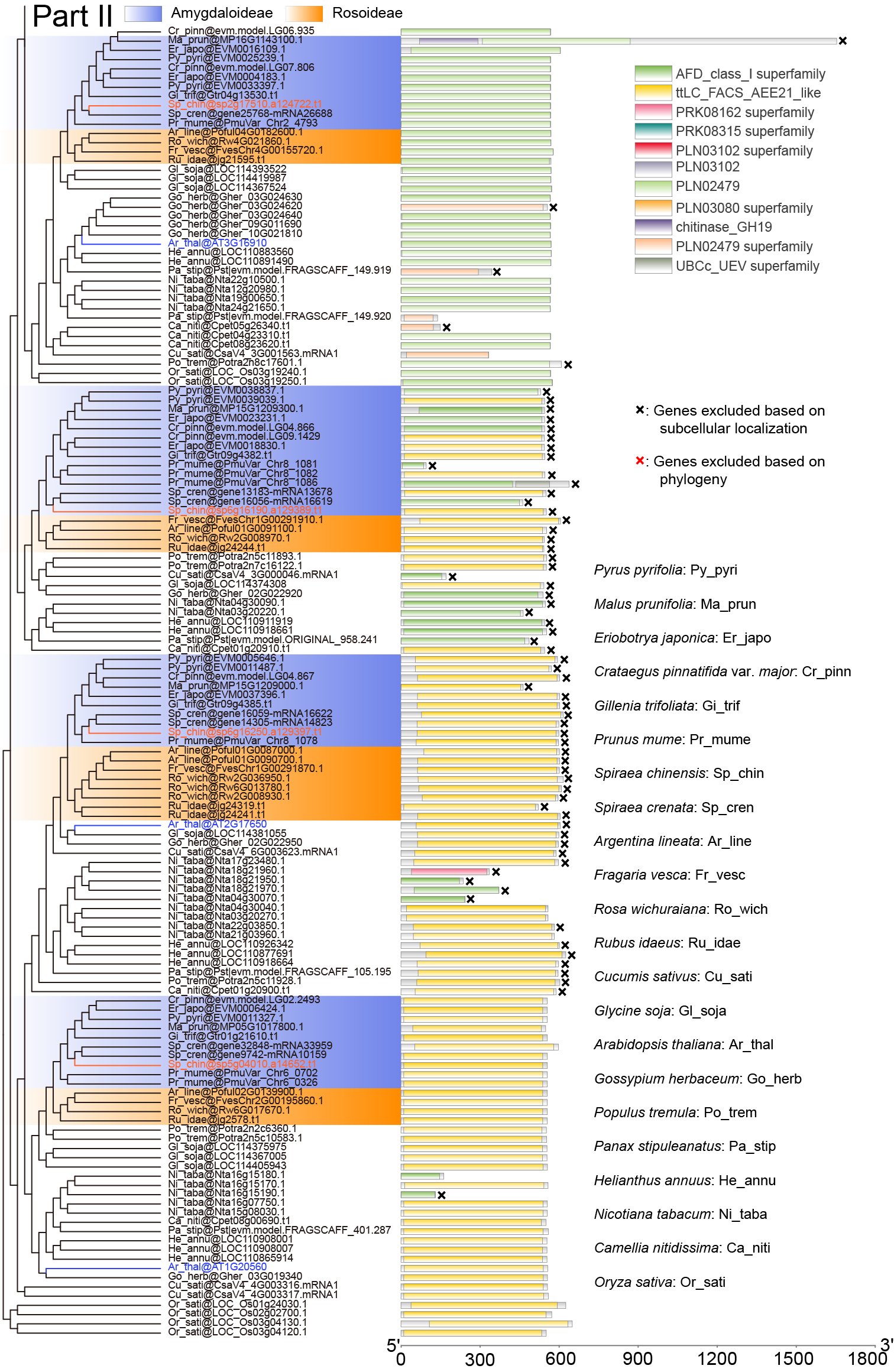
**

**Figure S25b. Maximum likelihood phylogenetic tree of CNL gene copies involved in the PAL-mediated SA biosynthesis pathway across Rosaceae species and representative outgroups (Part II).** Annotation details are consistent with those shown in Fig. S20.


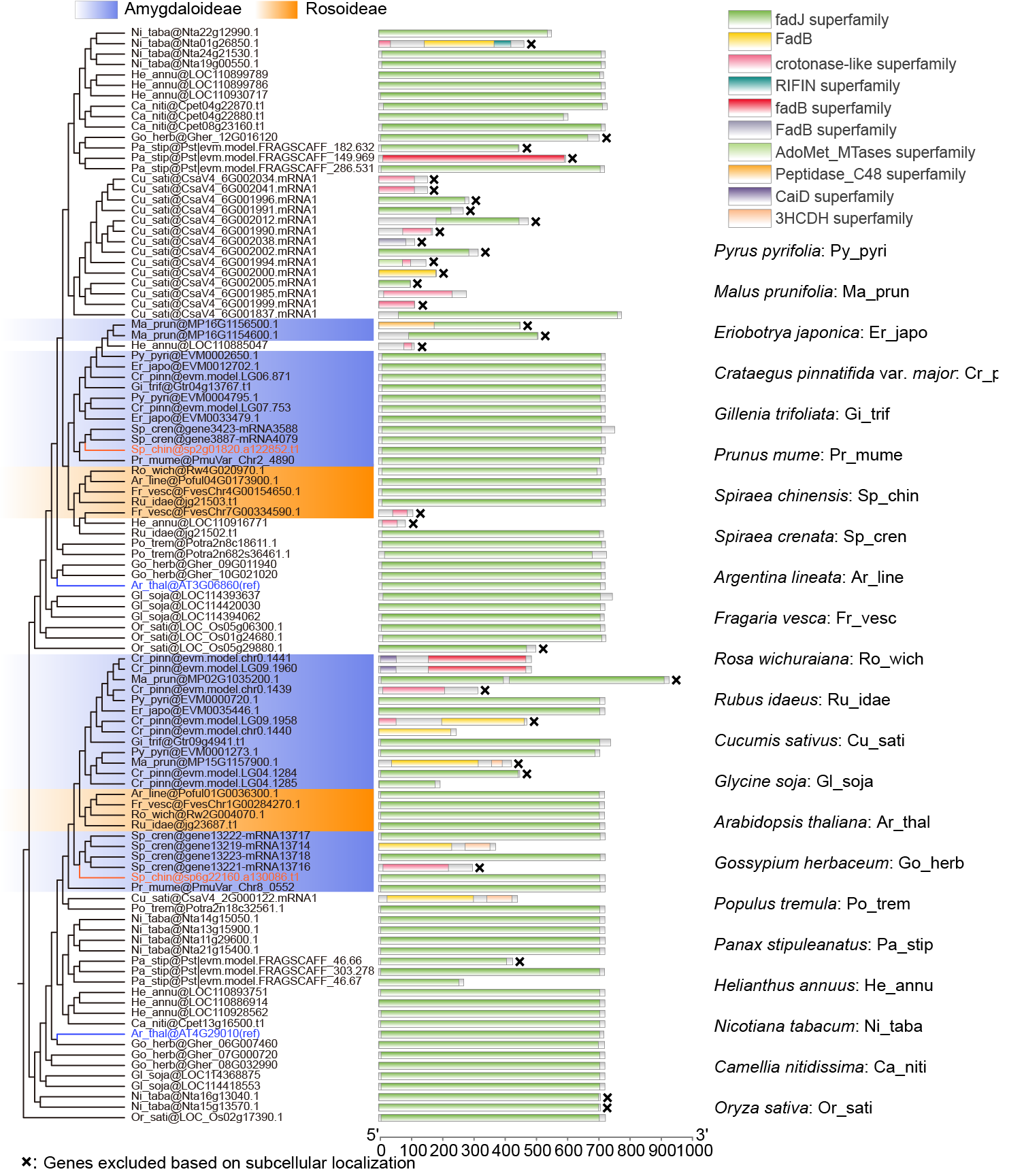


**Figure S26. Maximum likelihood phylogenetic tree of *CHD* gene copies involved in the PAL-mediated SA biosynthesis pathway across Rosaceae species and representative outgroups.** Annotation details are consistent with those shown in Fig. S20.


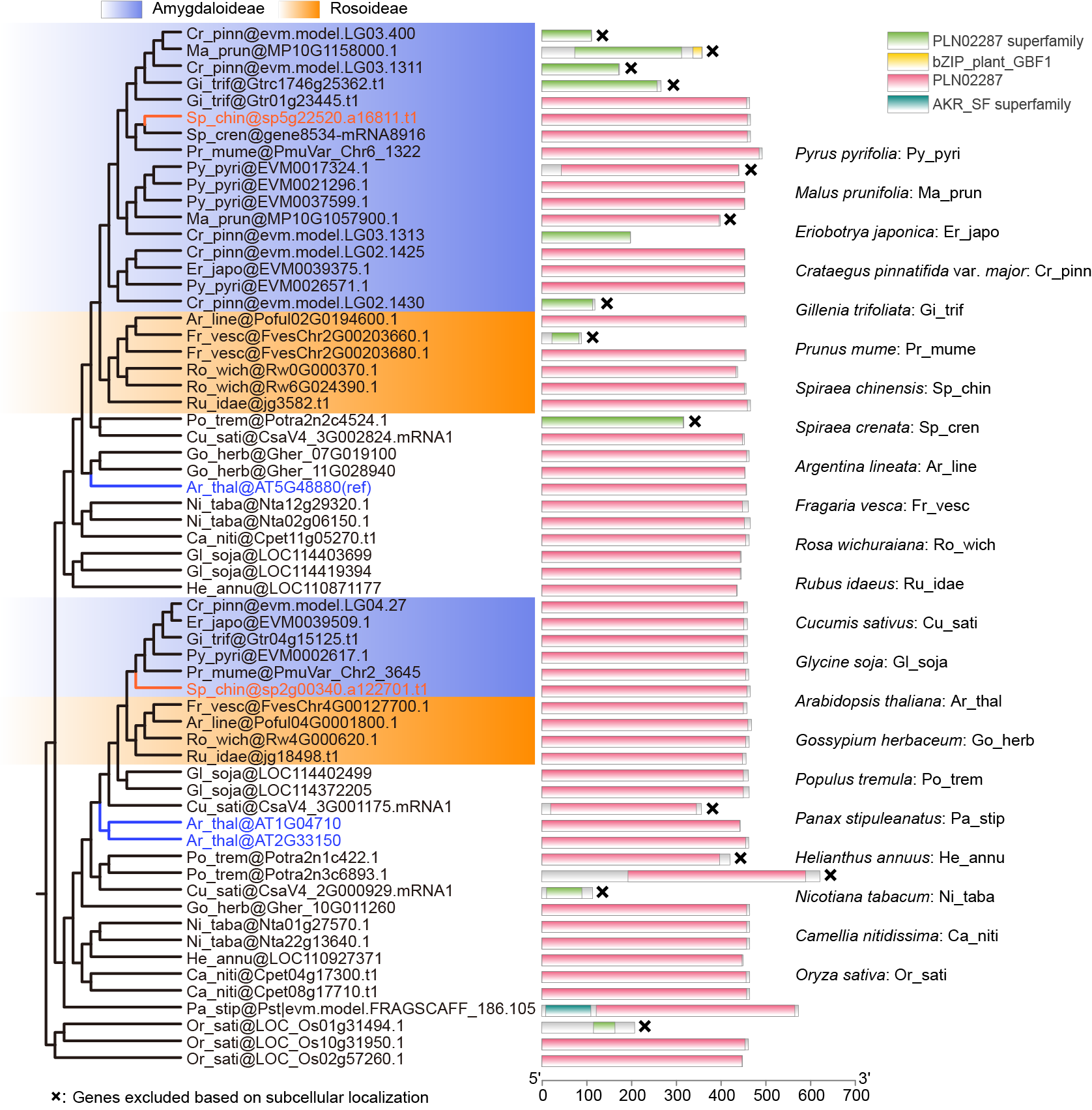


**Figure S27. Maximum likelihood phylogenetic tree of KAT gene copies involved in the PAL-mediated SA biosynthesis pathway across Rosaceae species and representative outgroups.** Annotation details are consistent with those shown in Fig. S20.


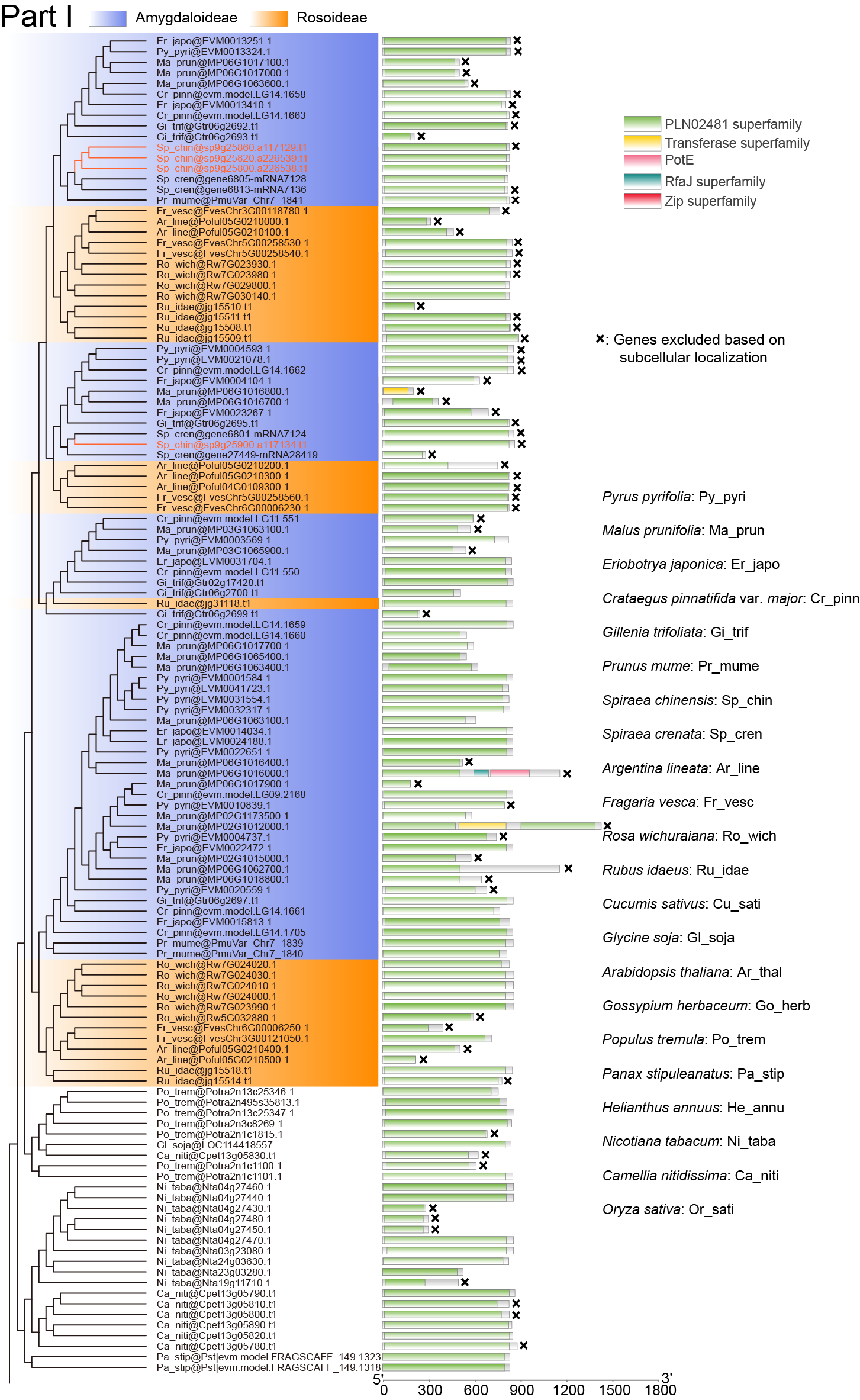


**Figure S28a. Maximum likelihood phylogenetic tree of BEBT gene copies involved in the PAL-mediated SA biosynthesis pathway across Rosaceae species and representative outgroups (Part I).** Annotation details are consistent with those shown in Fig. S20.


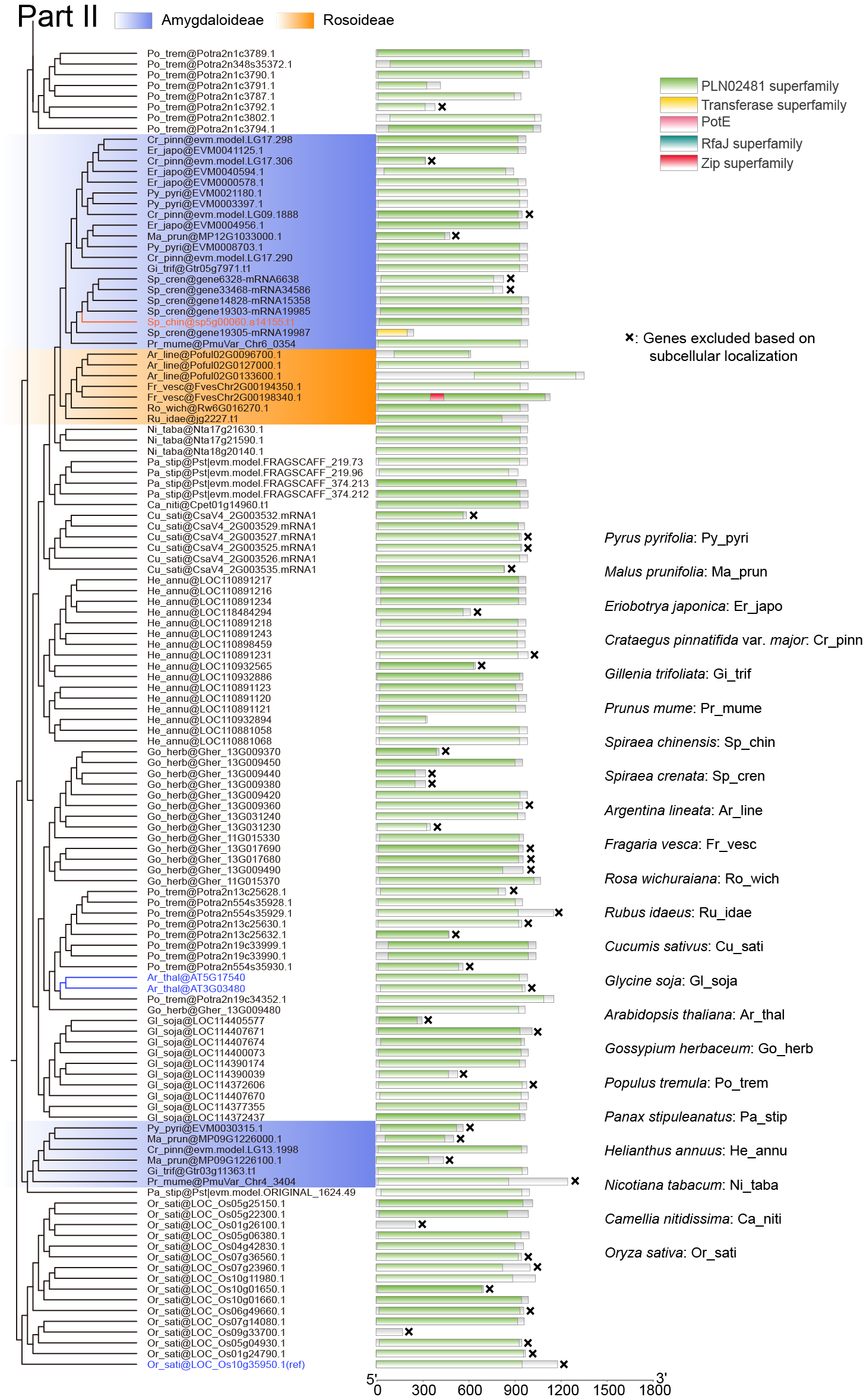


**Figure S28b. Maximum likelihood phylogenetic tree of BEBT gene copies involved in the PAL-mediated SA biosynthesis pathway across Rosaceae species and representative outgroups (Part II).** Annotation details are consistent with those shown in Fig. S20.


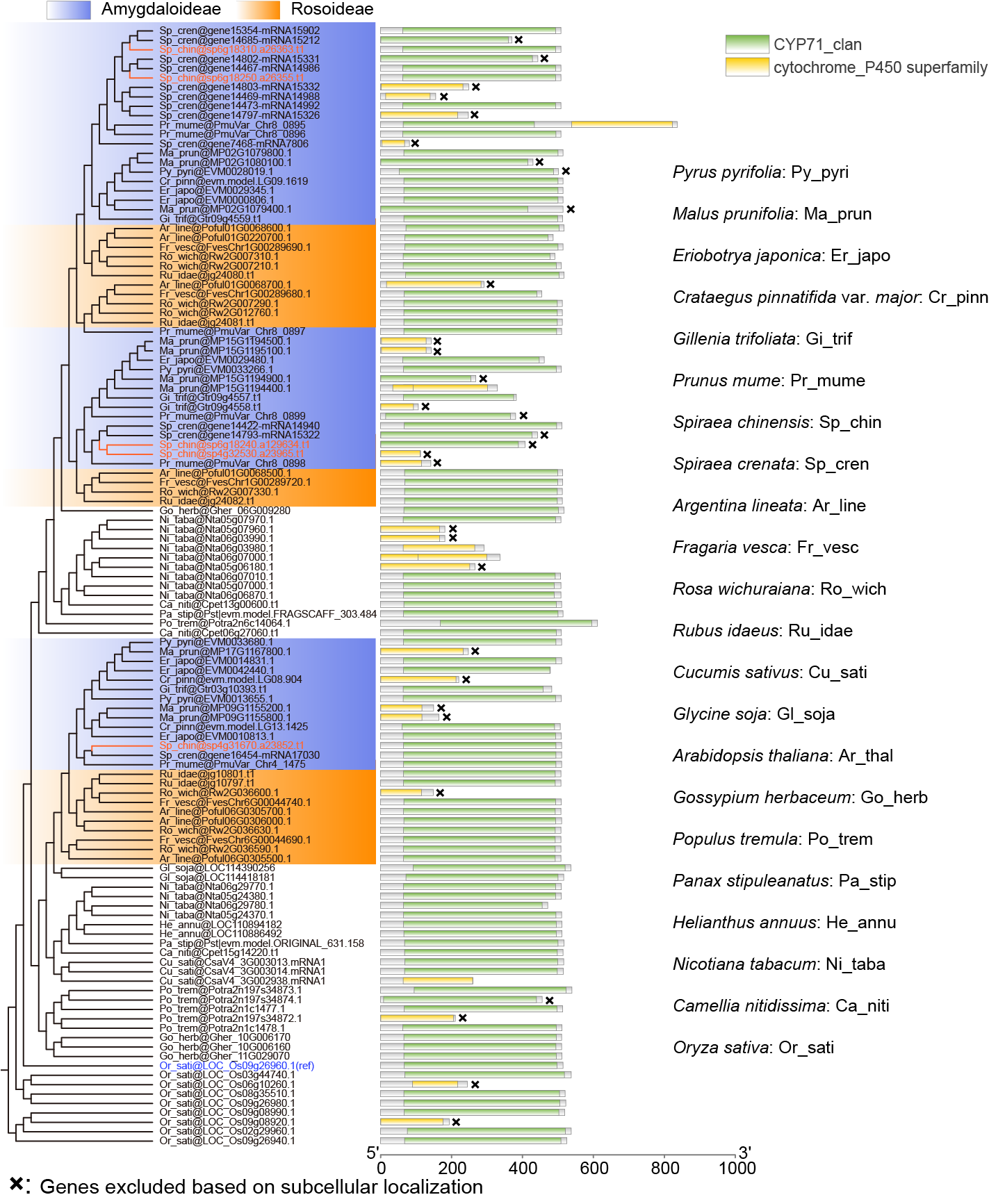


**Figure S29. Maximum likelihood phylogenetic tree of BBO gene copies involved in the PAL-mediated SA biosynthesis pathway across Rosaceae species and representative outgroups.** Annotation details are consistent with those shown in Fig. S20.


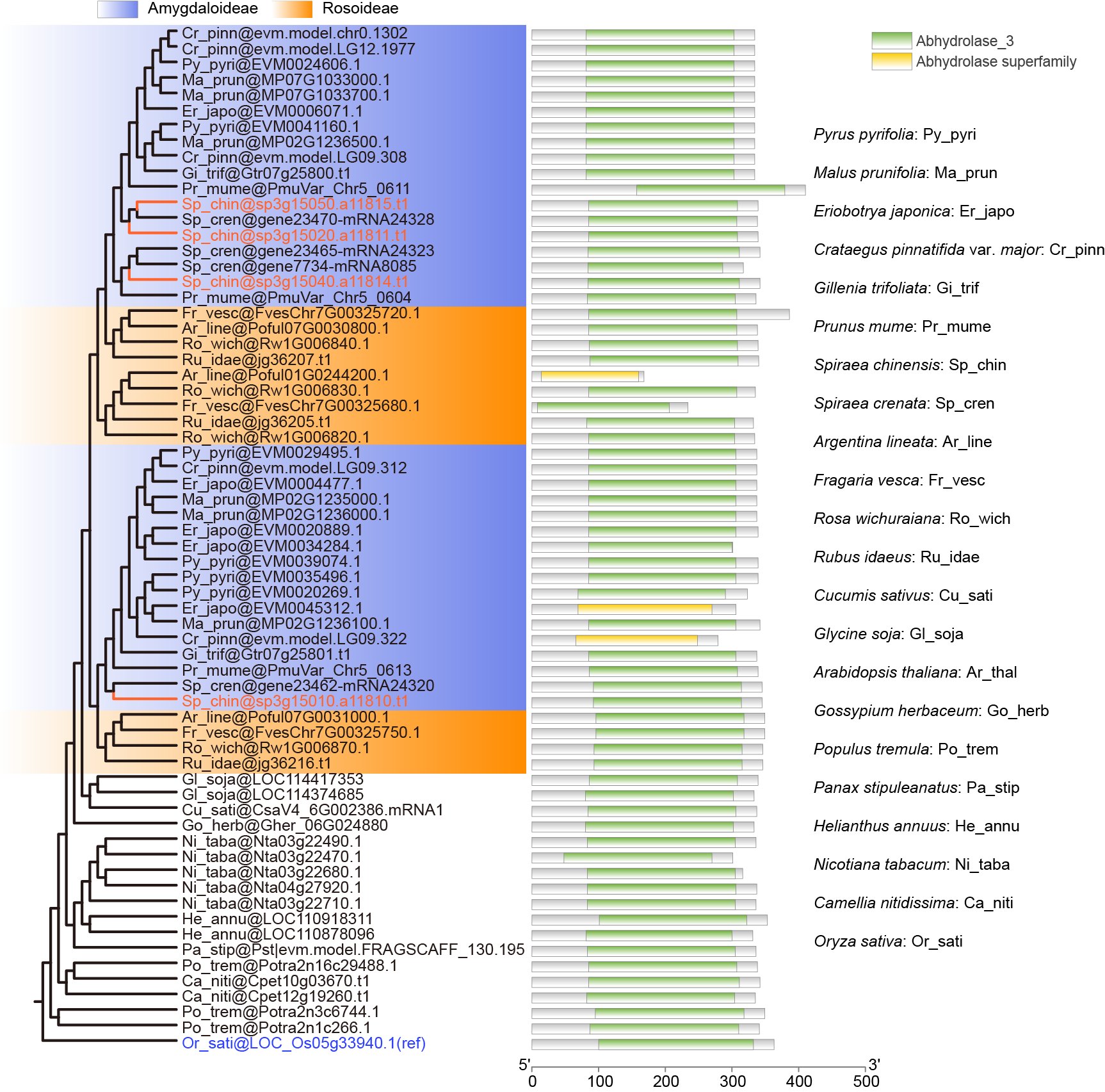


**Figure S30. Maximum likelihood phylogenetic tree of BSH gene copies involved in the PAL-mediated SA biosynthesis pathway across Rosaceae species and representative outgroups.** Annotation details are consistent with those shown in Fig. S20.


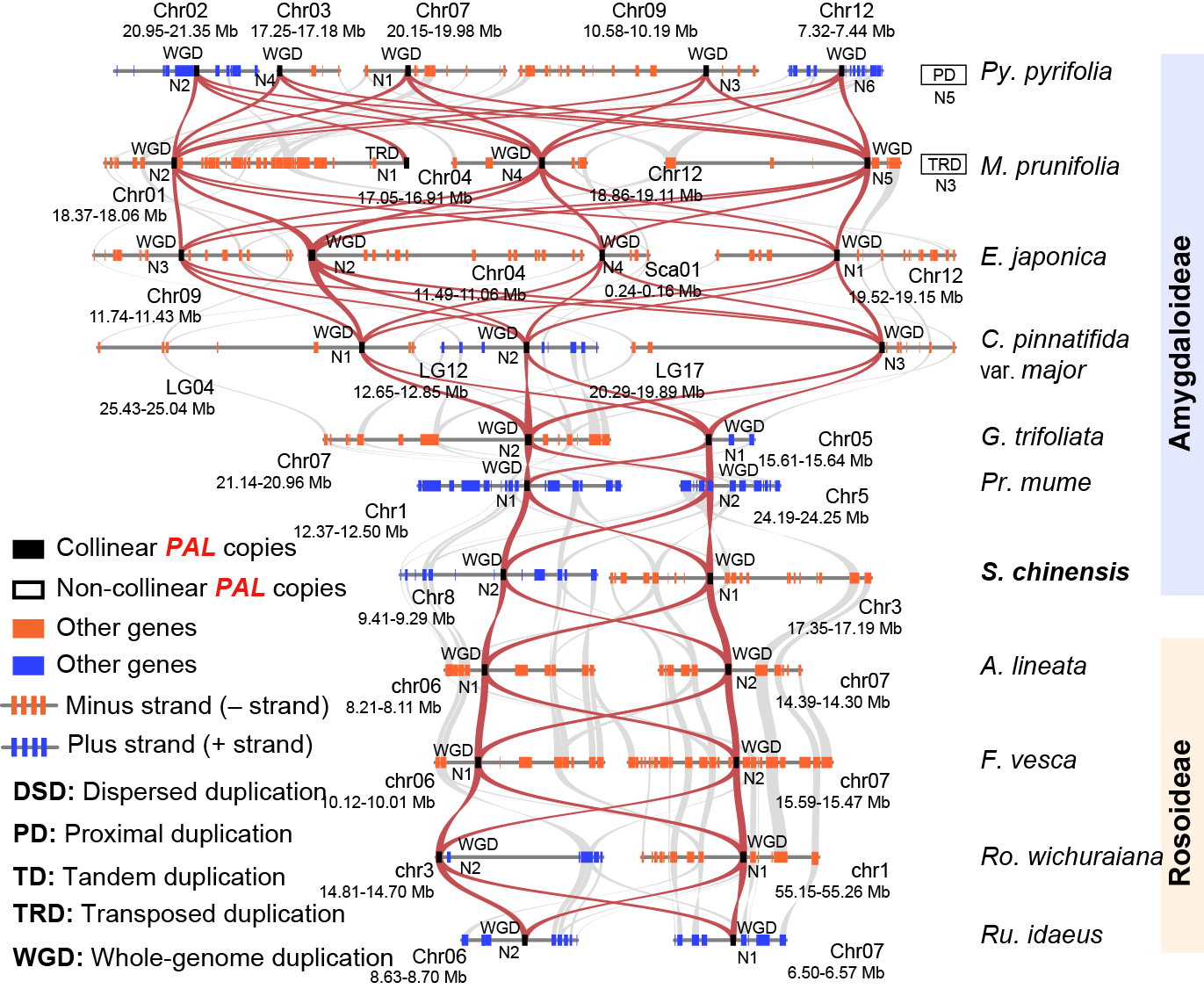


**Figure S31. Local synteny relationships of *PAL* gene copies involved in the PAL route at the single-gene level in Rosaceae, illustrating their evolutionary trajectories.** Each row represents the corresponding genomic regions from one species, with species information shown on the left. Syntenic *PAL* genes are shown as black blocks, non-syntenic *PAL* genes as open black boxes on the right, and other neighboring genes in orange or blue. Red curves connect syntenic *PAL* genes, while gray curves connect other collinear pairs. Gene copy IDs (N1–N6) and duplication modes (WGD, TRD, DSD, TD, PD) are annotated near each gene. Further details are available in **Table S26**.


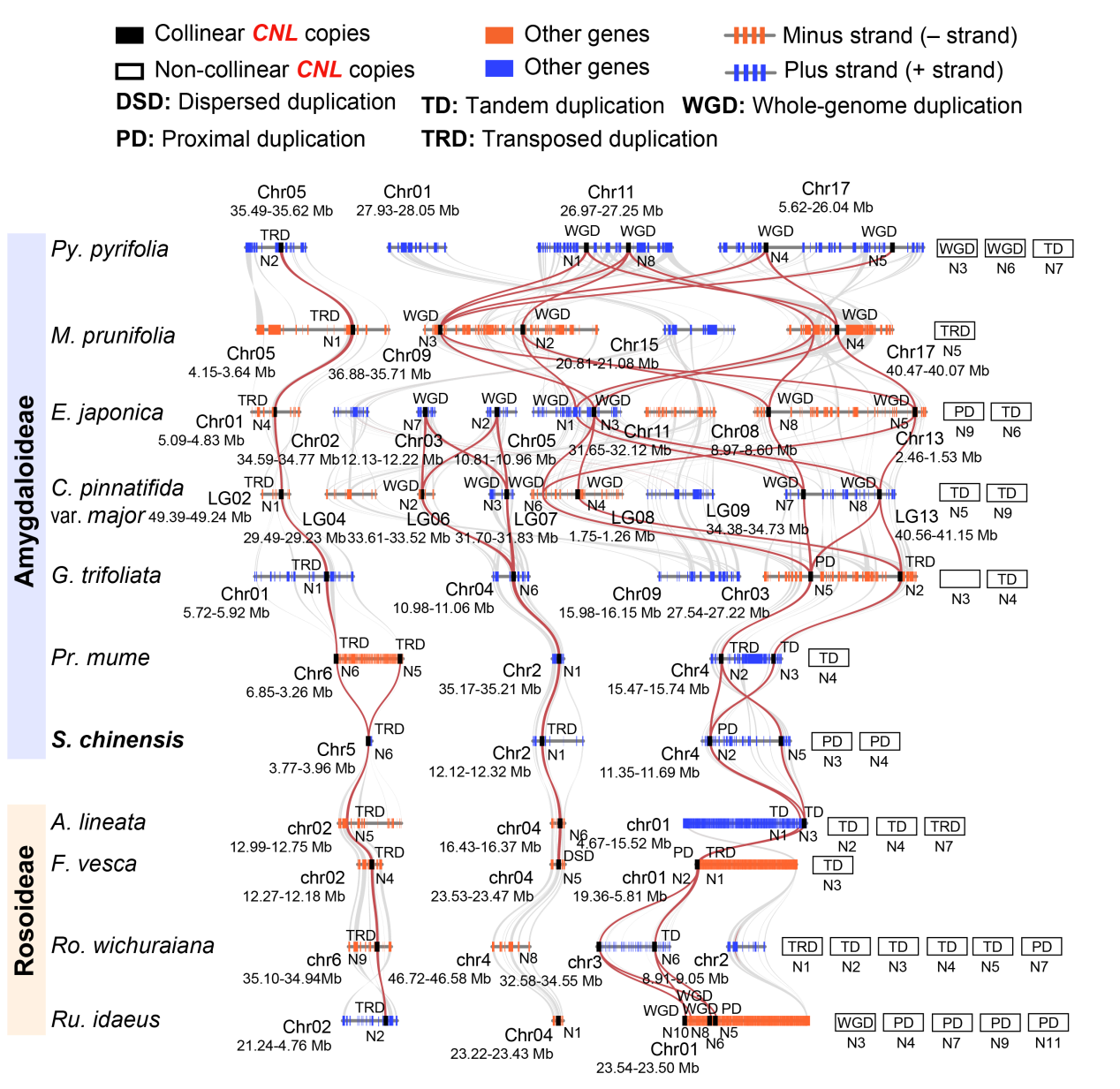


**Figure S32. Local synteny relationships of *CNL* gene copies involved in the PAL route at the single-gene level in Rosaceae, illustrating their evolutionary trajectories.** Each row represents the corresponding genomic regions from one species, with species information shown on the left. Syntenic *CNL* genes are shown as black blocks, non-syntenic *CNL* genes as open black boxes on the right, and other neighboring genes in orange or blue. Red curves connect syntenic *CNL* genes, while gray curves connect other collinear pairs. Gene copy IDs (N1–N9) and duplication modes (WGD, TRD, DSD, TD, PD) are annotated near each gene. Further details are available in **Table S26**.


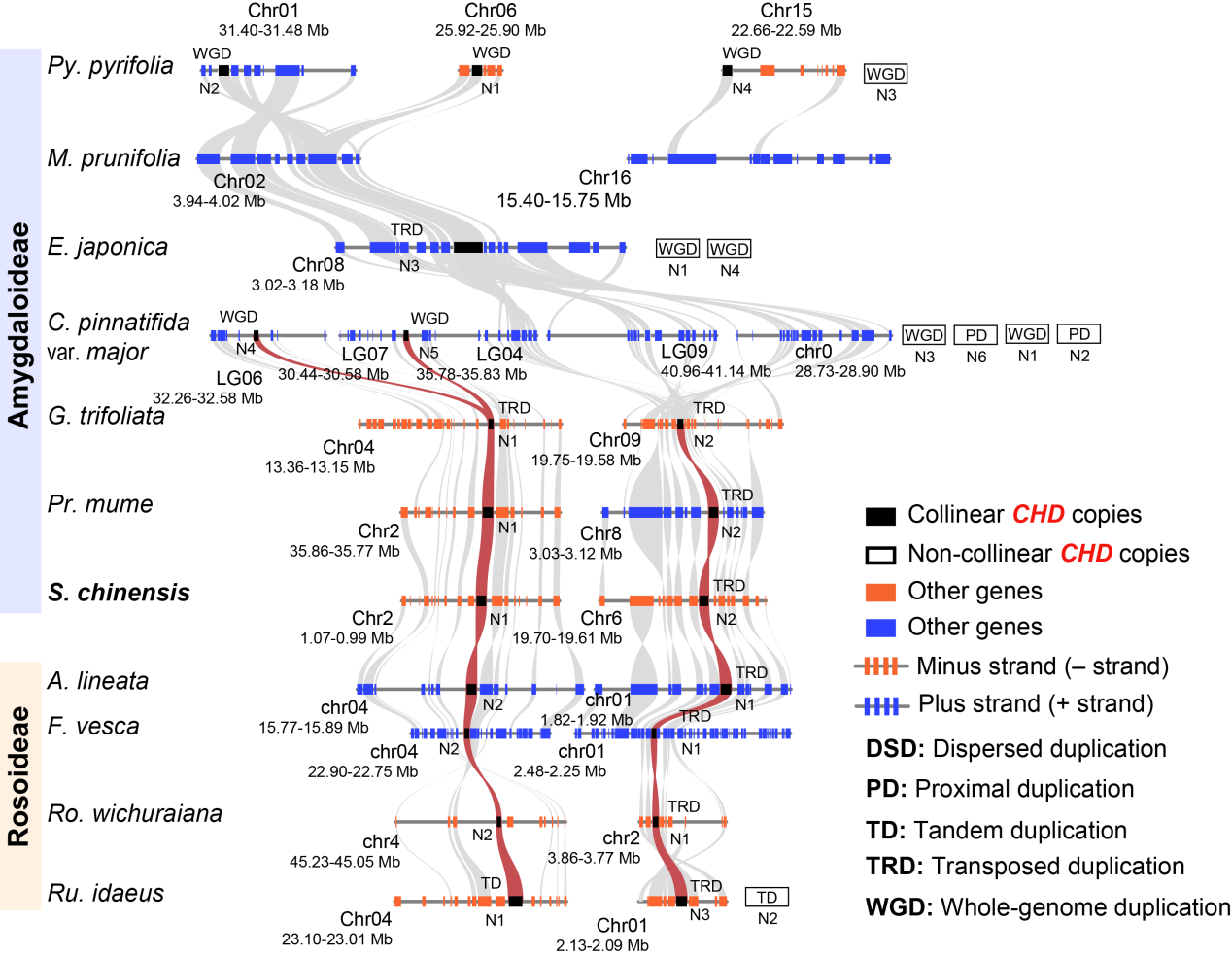


**Figure S33. Local synteny relationships of *CHD* gene copies involved in the PAL route at the single-gene level in Rosaceae, illustrating their evolutionary trajectories.** Each row represents the corresponding genomic regions from one species, with species information shown on the left. Syntenic *CHD* genes are shown as black blocks, non-syntenic *CHD* genes as open black boxes on the right, and other neighboring genes in orange or blue. Red curves connect syntenic *CHD* genes, while gray curves connect other collinear pairs. Gene copy IDs (N1–N6) and duplication modes (WGD, TRD, DSD, TD, PD) are annotated near each gene. Further details are available in **Table S26**.


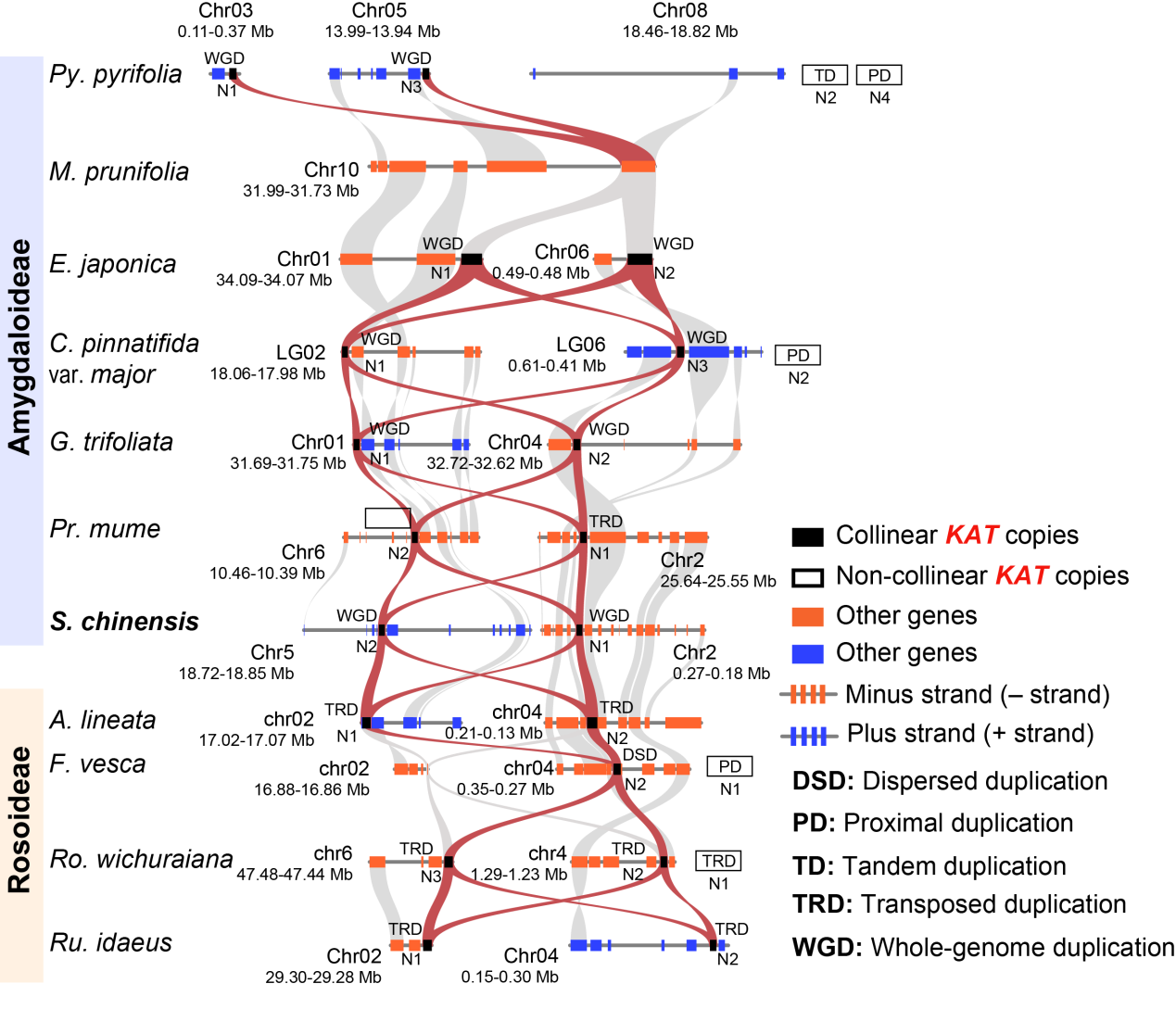


**Figure S34. Local synteny relationships of *KAT* gene copies involved in the PAL route at the single-gene level in Rosaceae, illustrating their evolutionary trajectories.** Each row represents the corresponding genomic regions from one species, with species information shown on the left. Syntenic *KAT* genes are shown as black blocks, non-syntenic *KAT* genes as open black boxes on the right, and other neighboring genes in orange or blue. Red curves connect syntenic *KAT* genes, while gray curves connect other collinear pairs. Gene copy IDs (N1–N4) and duplication modes (WGD, TRD, DSD, TD, PD) are annotated near each gene. Further details are available in **Table S26**.


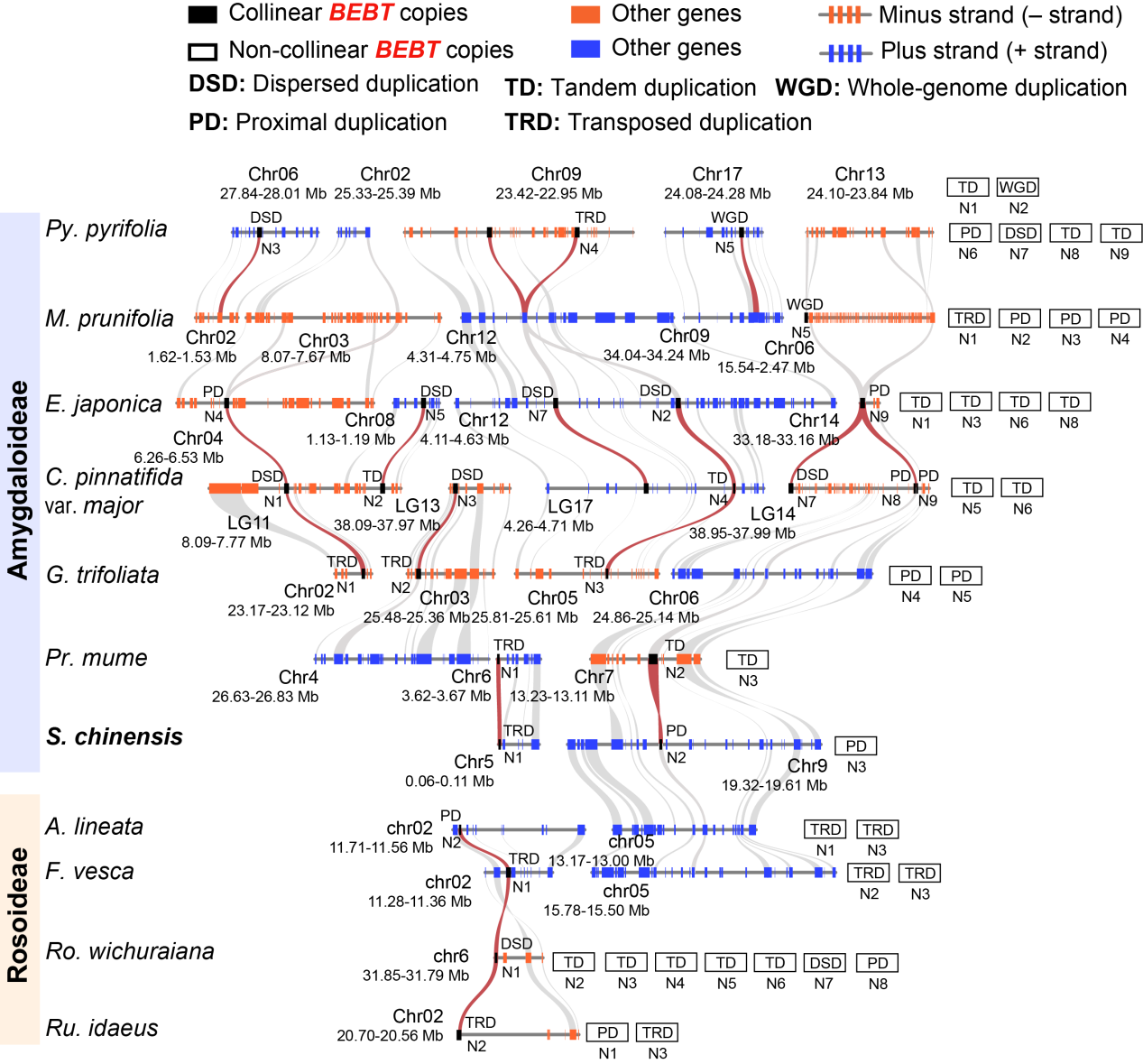


**Figure S35. Local synteny relationships of *BEBT* gene copies involved in the PAL route at the single-gene level in Rosaceae, illustrating their evolutionary trajectories.** Each row represents the corresponding genomic regions from one species, with species information shown on the left. Syntenic *BEBT* genes are shown as black blocks, non-syntenic *BEBT* genes as open black boxes on the right, and other neighboring genes in orange or blue. Red curves connect syntenic *BEBT* genes, while gray curves connect other collinear pairs. Gene copy IDs (N1–N9) and duplication modes (WGD, TRD, DSD, TD, PD) are annotated near each gene. Further details are available in **Table S26**.


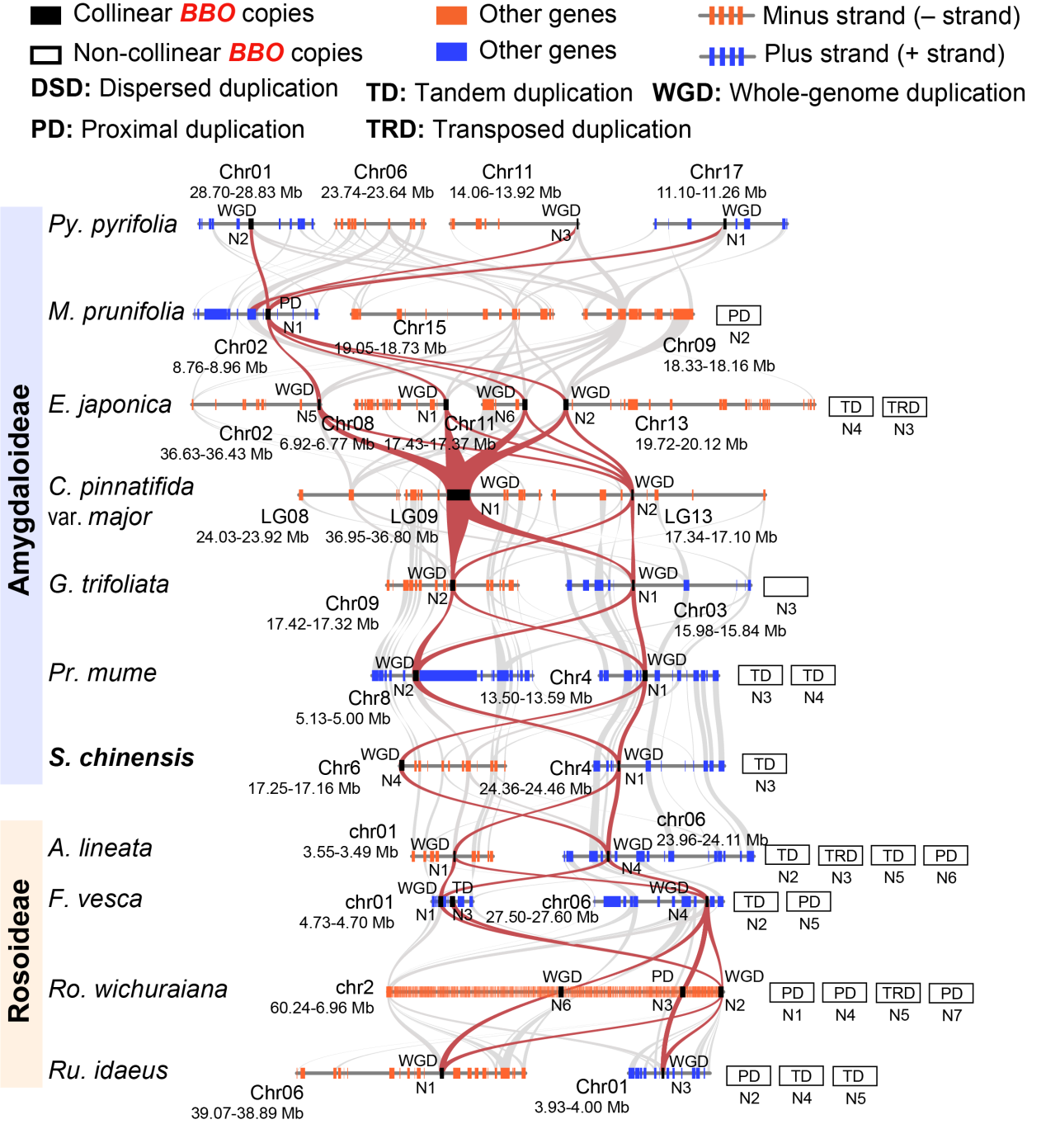


**Figure S36. Local synteny relationships of *BBO* gene copies involved in the PAL route at the single-gene level in Rosaceae, illustrating their evolutionary trajectories.** Each row represents the corresponding genomic regions from one species, with species information shown on the left. Syntenic *BBO* genes are shown as black blocks, non-syntenic *BBO* genes as open black boxes on the right, and other neighboring genes in orange or blue. Red curves connect syntenic *BBO* genes, while gray curves connect other collinear pairs. Gene copy IDs (N1–N7) and duplication modes (WGD, TRD, DSD, TD, PD) are annotated near each gene. Further details are available in **Table S26**.


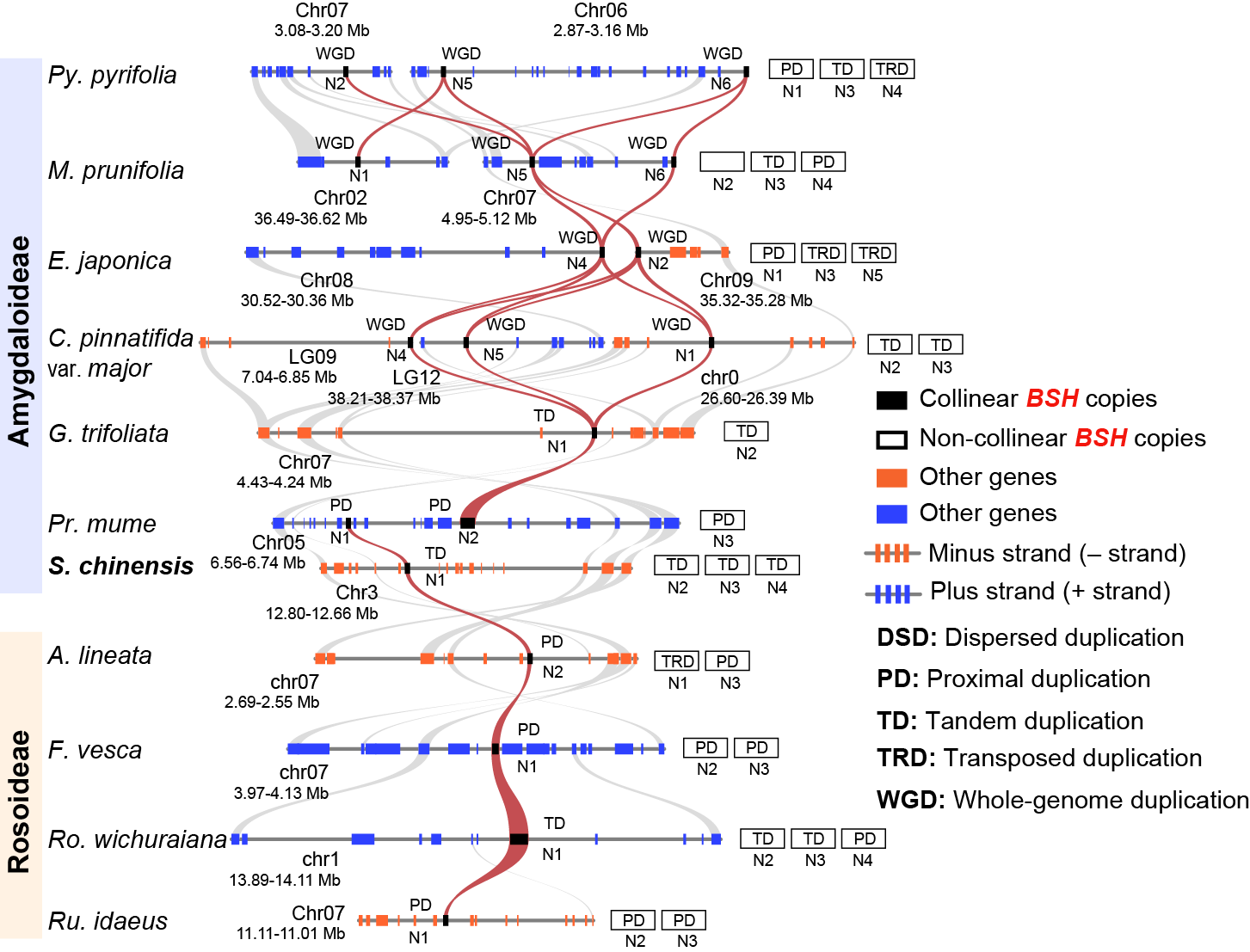


**Figure S37. Local synteny relationships of *BSH* gene copies involved in the PAL route at the single-gene level in Rosaceae, illustrating their evolutionary trajectories.** Each row represents the corresponding genomic regions from one species, with species information shown on the left. Syntenic *BSH* genes are shown as black blocks, non-syntenic *BSH* genes as open black boxes on the right, and other neighboring genes in orange or blue. Red curves connect syntenic *BSH* genes, while gray curves connect other collinear pairs. Gene copy IDs (N1–N6) and duplication modes (WGD, TRD, DSD, TD, PD) are annotated near each gene. Further details are available in **Table S26**.


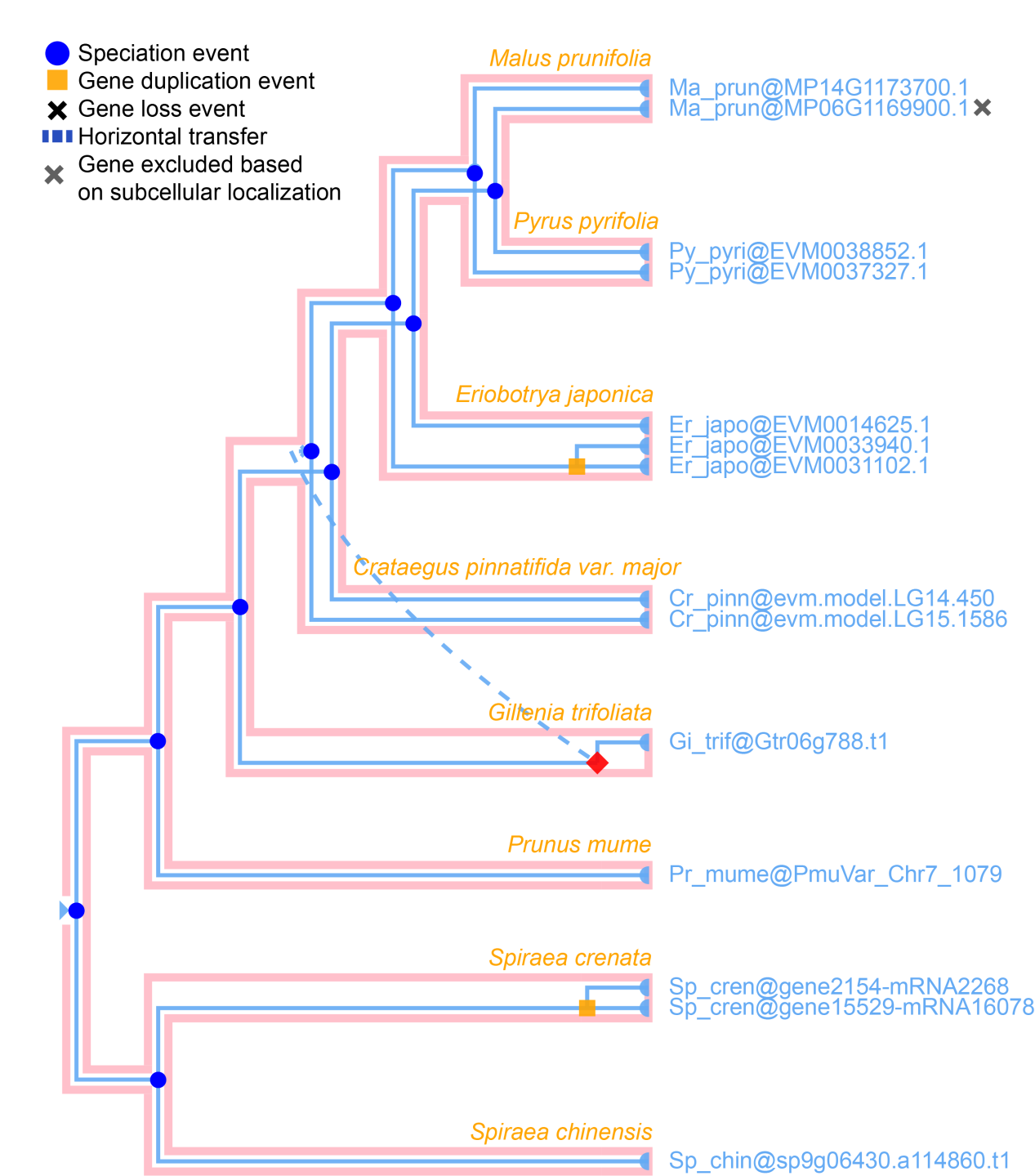


**Figure S38. Gene tree reconciliation and evolutionary history of the *ICS1* gene family involved in the ICS route across eight Amygdaloideae species.** The reconciliation analysis was performed using GeneRax, showing the mapping of the gene tree (blue lines) onto the inferred species tree (thick pink outlines). Circles at the branching points of the gene tree represent speciation events. Squares within the branches indicate gene duplication events. The black "×" symbols denote gene loss events. Dotted lines represent inferred horizontal transfers. Gray "×" symbols denote genes excluded on the basis of inconsistent subcellular localization, with details provided in Table S24.


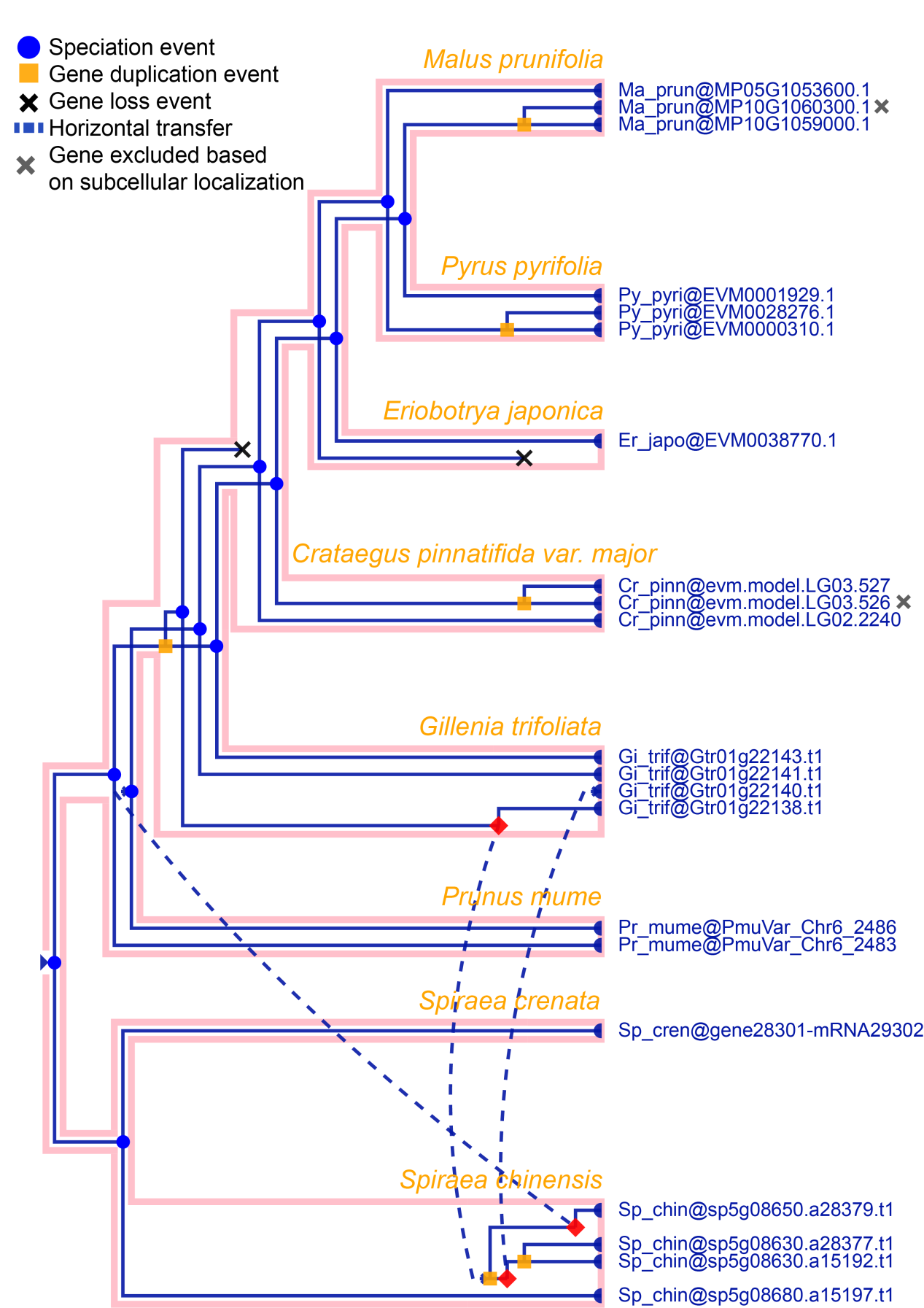


**Figure S39. Gene tree reconciliation and evolutionary history of the *EDS5* gene family involved in the ICS route across eight Amygdaloideae species.** Annotation details are consistent with those shown in Fig. S38.


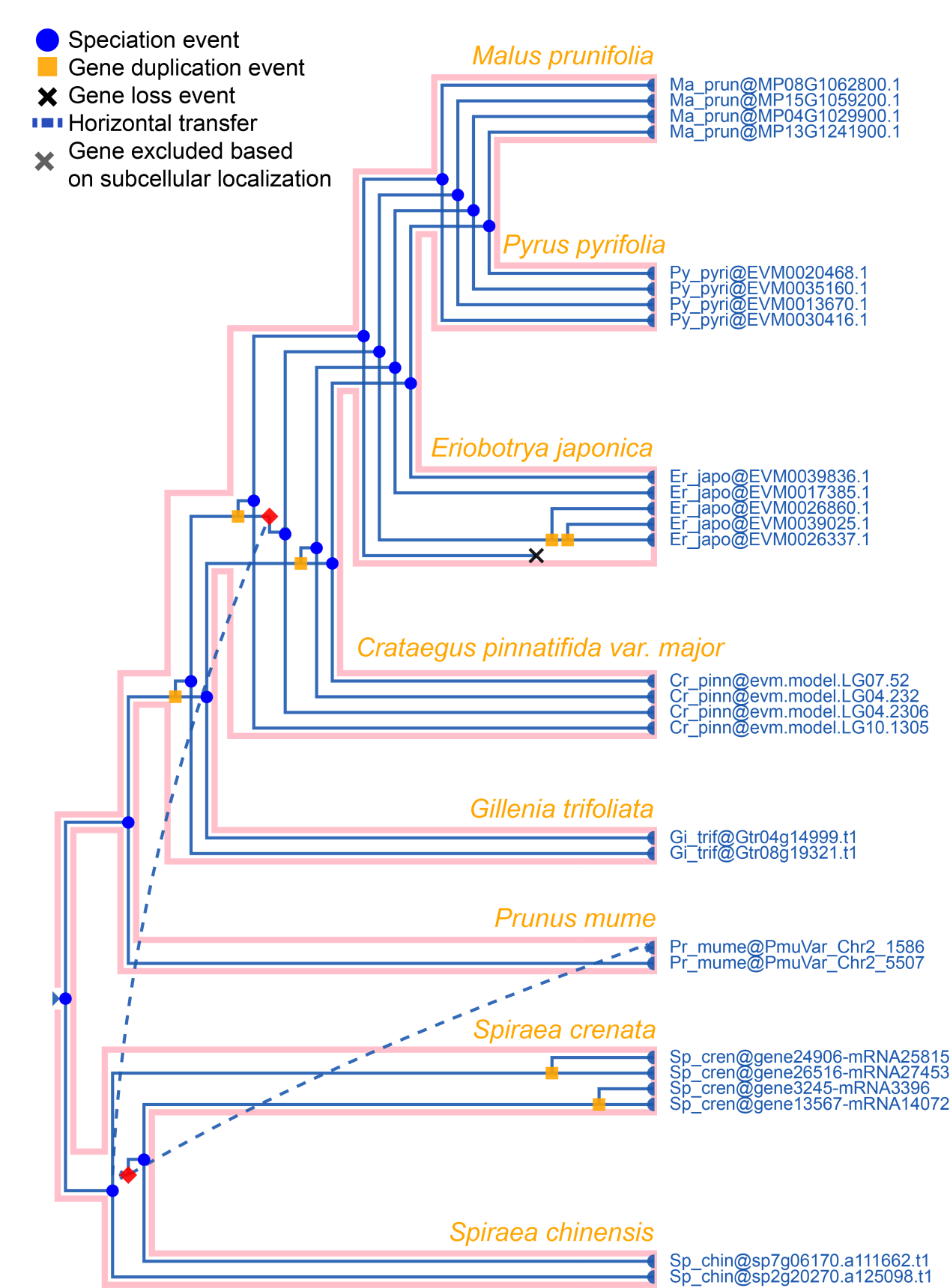


**Figure S40. Gene tree reconciliation and evolutionary history of the *EPS1* gene family involved in the ICS route across eight Amygdaloideae species.** Annotation details are consistent with those shown in Fig. S38.


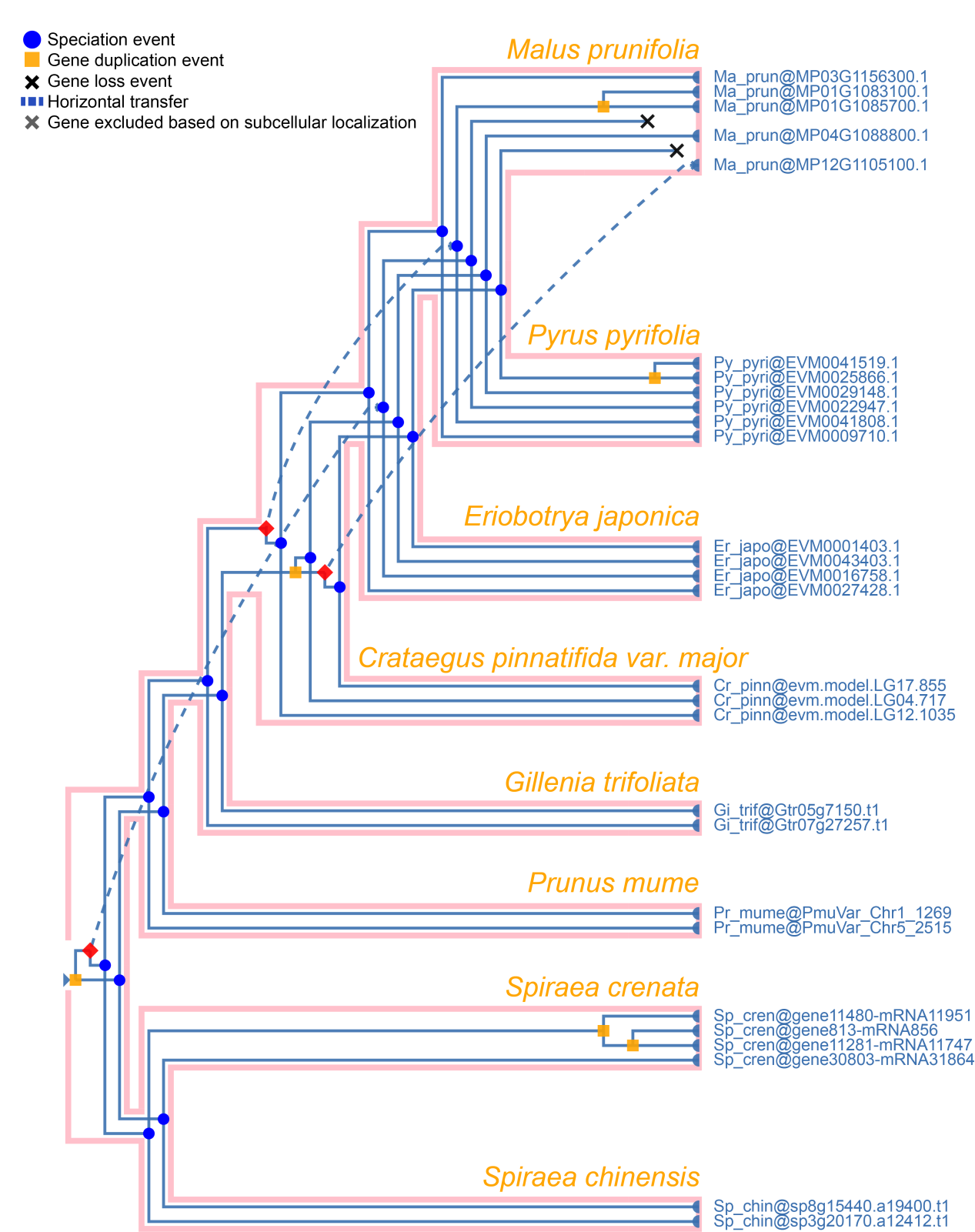


**Figure S41. Gene tree reconciliation and evolutionary history of the *PAL* gene family involved in the PAL route across eight Amygdaloideae species.** Annotation details are consistent with those shown in Fig. S38.


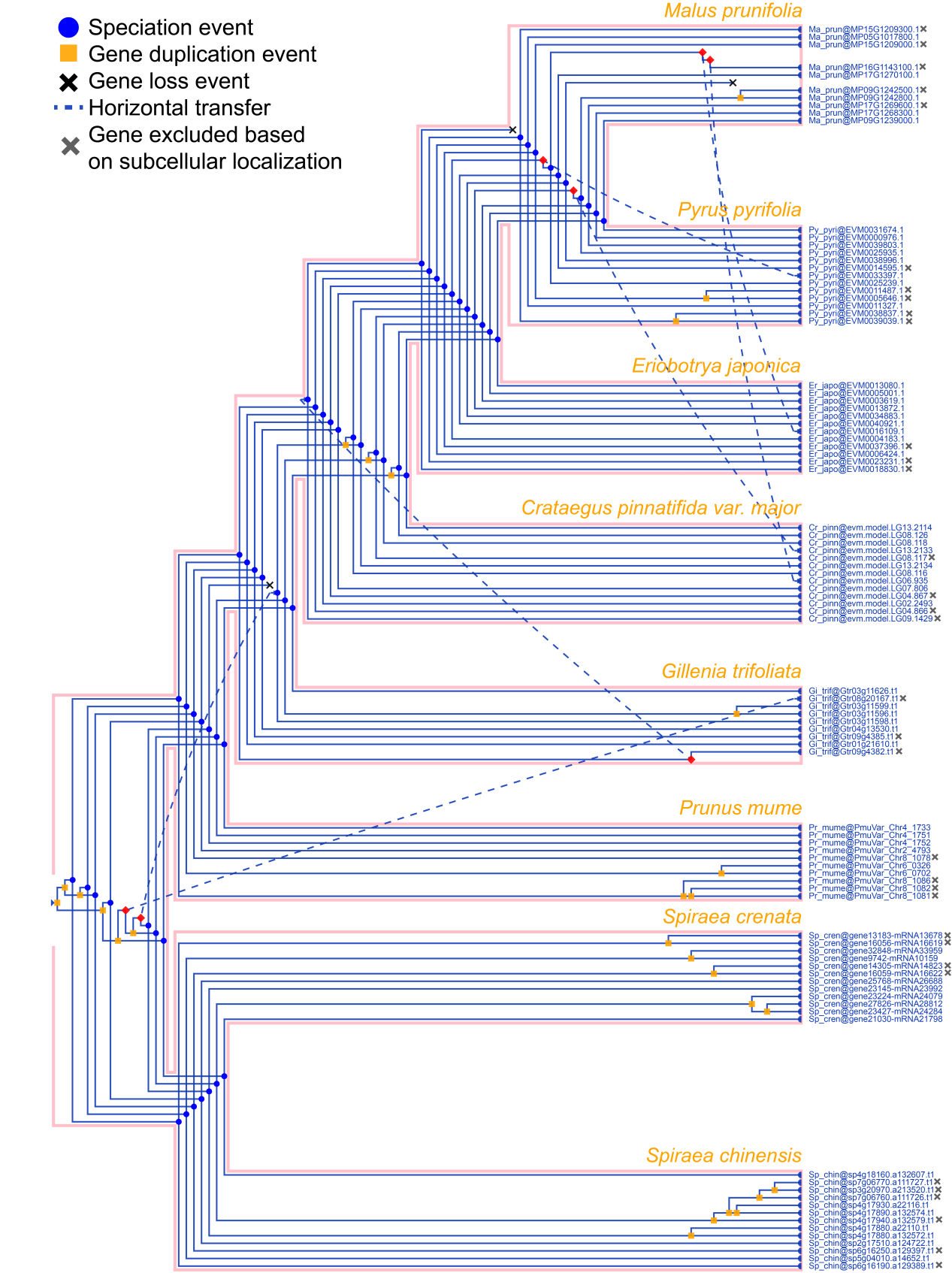


**Figure S42a. Gene tree reconciliation and evolutionary history of the *CNL* gene family involved in the PAL route across eight Amygdaloideae species.** Annotation details are consistent with those shown in Fig. S38.

**
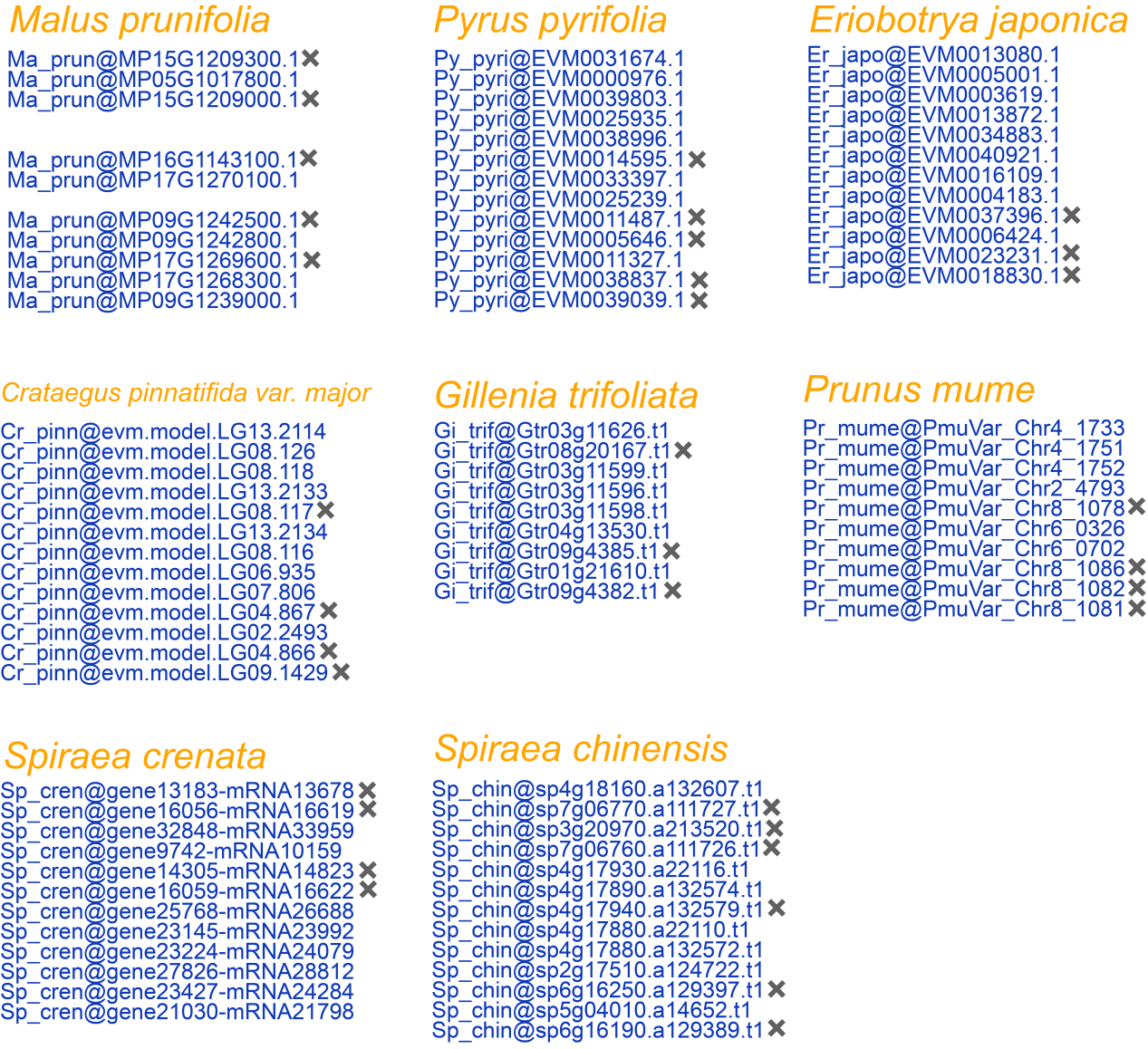
**

**Figure S42b. Ordered list of *CNL* gene names corresponding to the gene tree shown in Figure S30a, provided to enable detailed inspection and accurate cross-referencing of individual genes.**


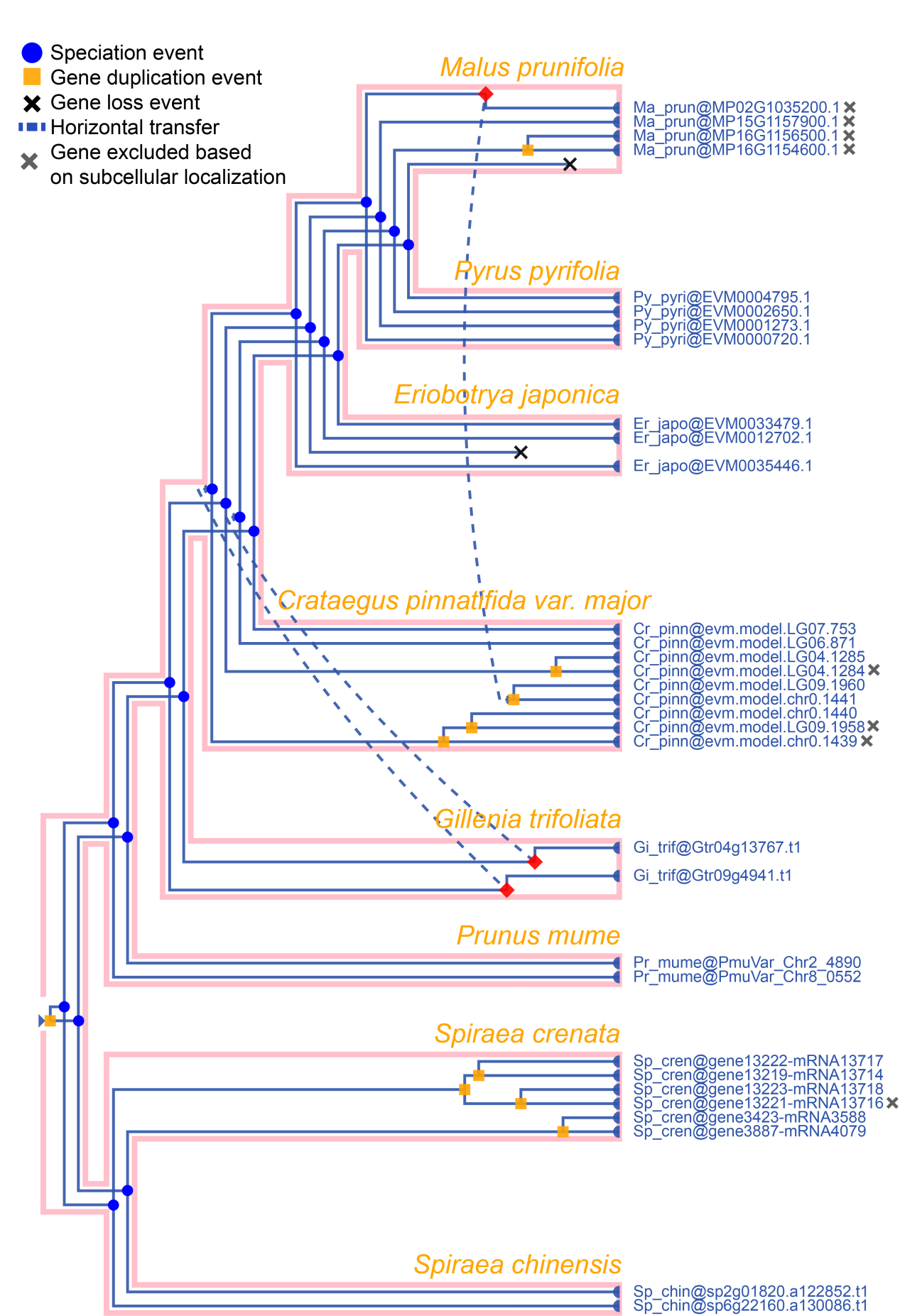


**Figure S43. Gene tree reconciliation and evolutionary history of the *CHD* gene family involved in the PAL route across eight Amygdaloideae species.** Annotation details are consistent with those shown in Fig. S38.


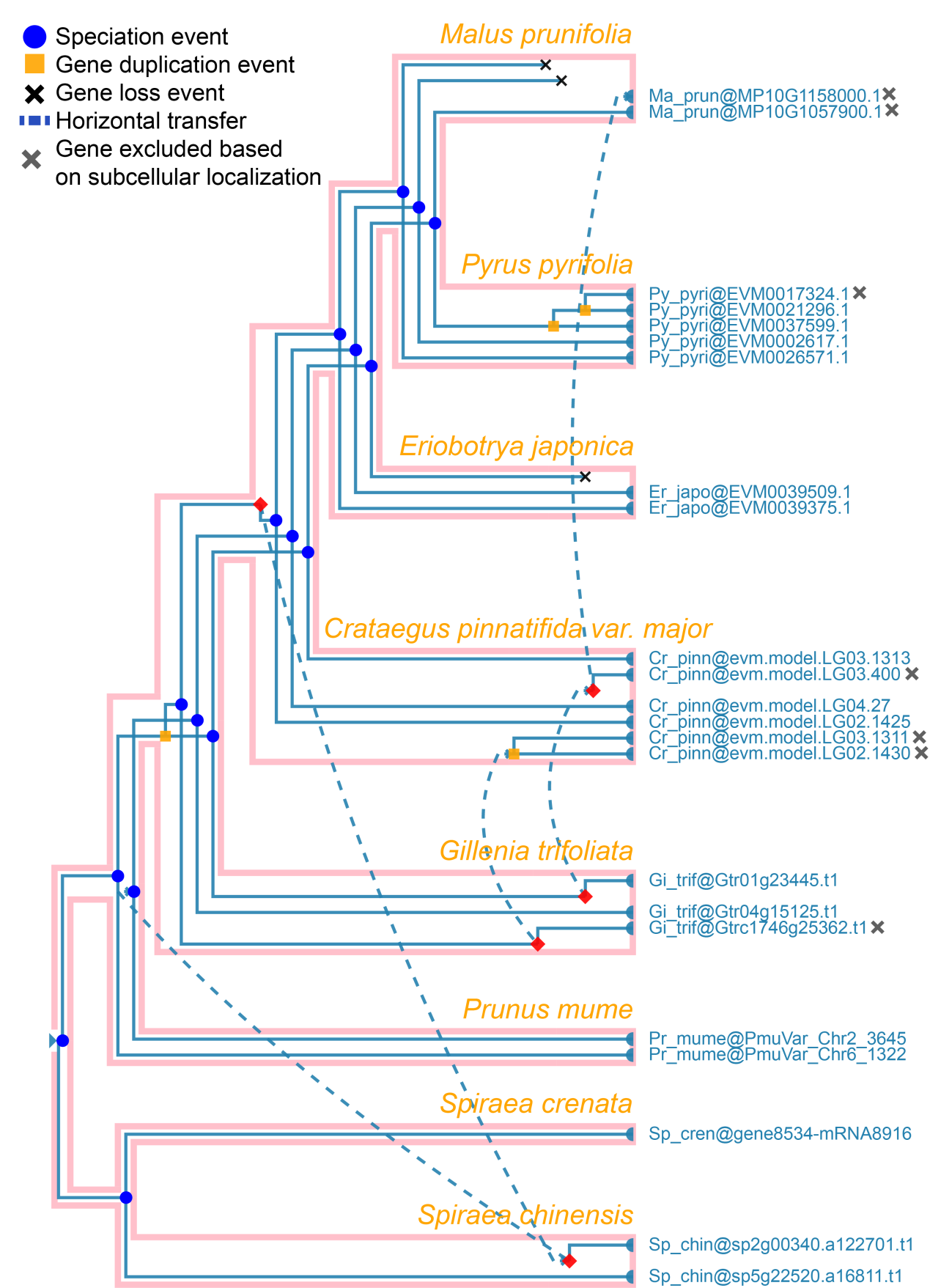


**Figure S44. Gene tree reconciliation and evolutionary history of the *KAT* gene family involved in the PAL route across eight Amygdaloideae species.** Annotation details are consistent with those shown in Fig. S38.


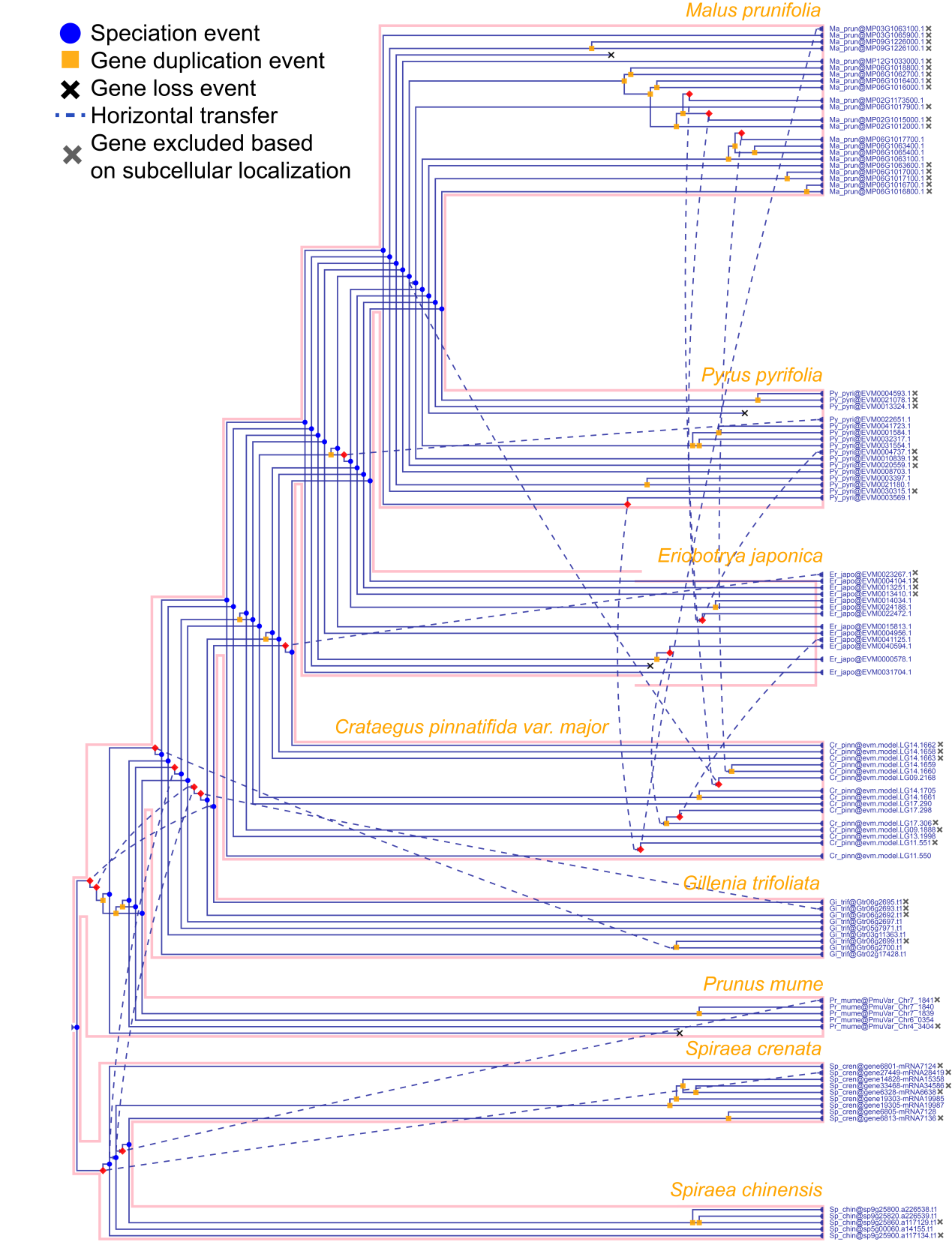


**Figure S45a. Gene tree reconciliation and evolutionary history of the *BEBT* gene family involved in the PAL route across eight Amygdaloideae species.** Annotation details are consistent with those shown in Fig. S38.

**
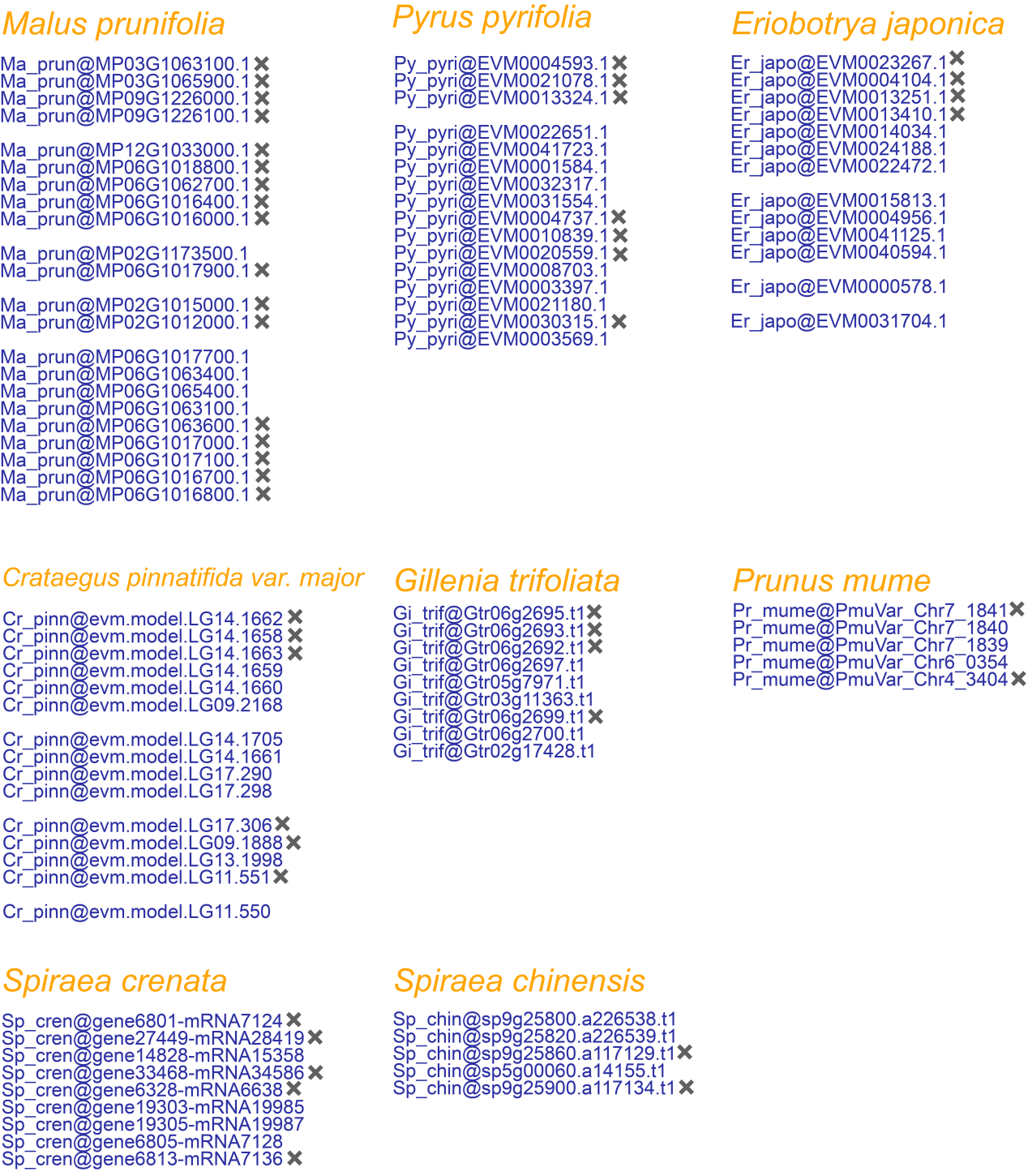
**

**Figure S45b. Ordered list of *BEBT* gene names corresponding to the gene tree shown in Figure S33a, provided to enable detailed inspection and accurate cross-referencing of individual genes.**


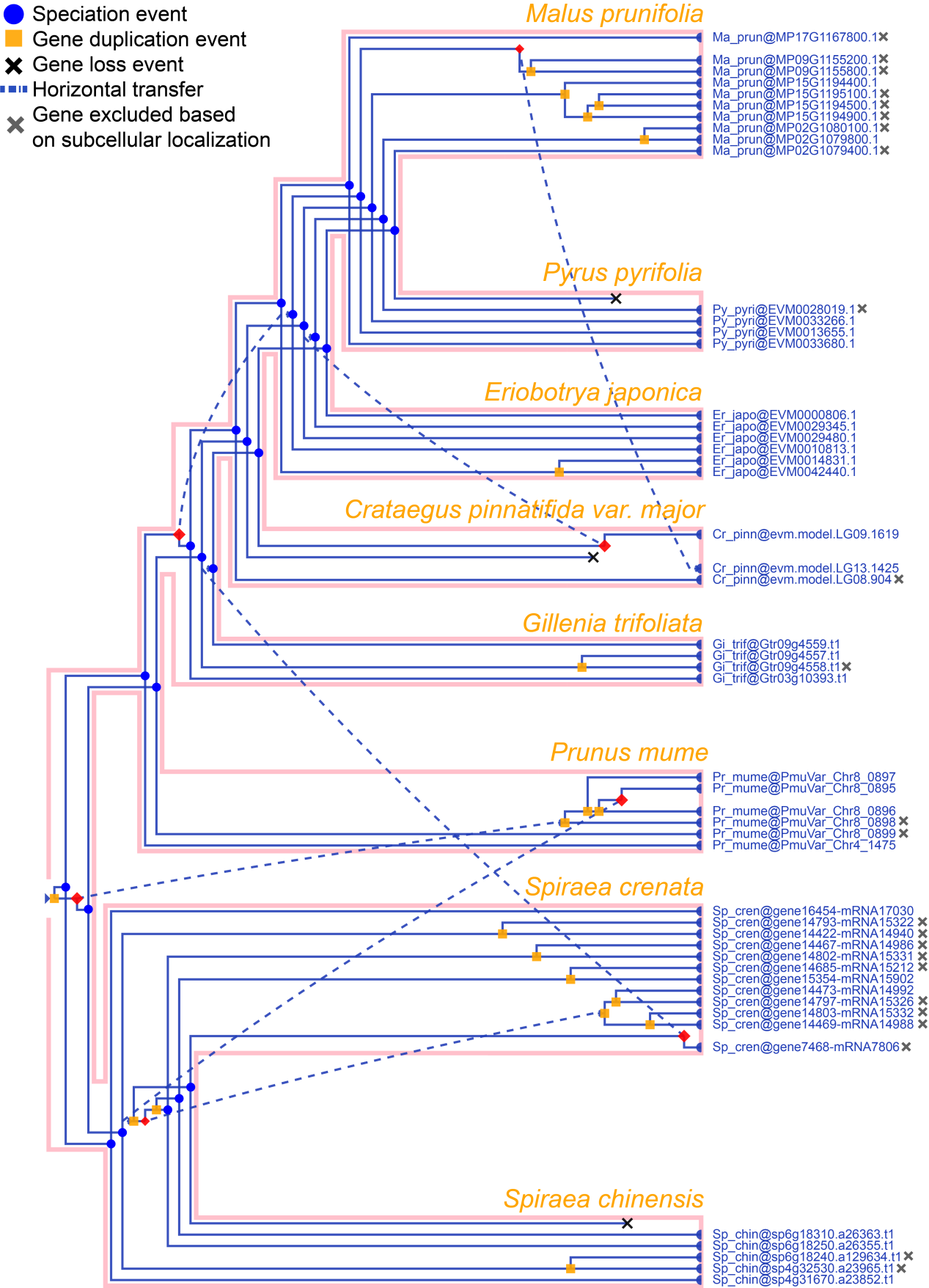


**Figure S46. Gene tree reconciliation and evolutionary history of the *BBO* gene family involved in the PAL route across eight Amygdaloideae species.** Annotation details are consistent with those shown in Fig. S38.


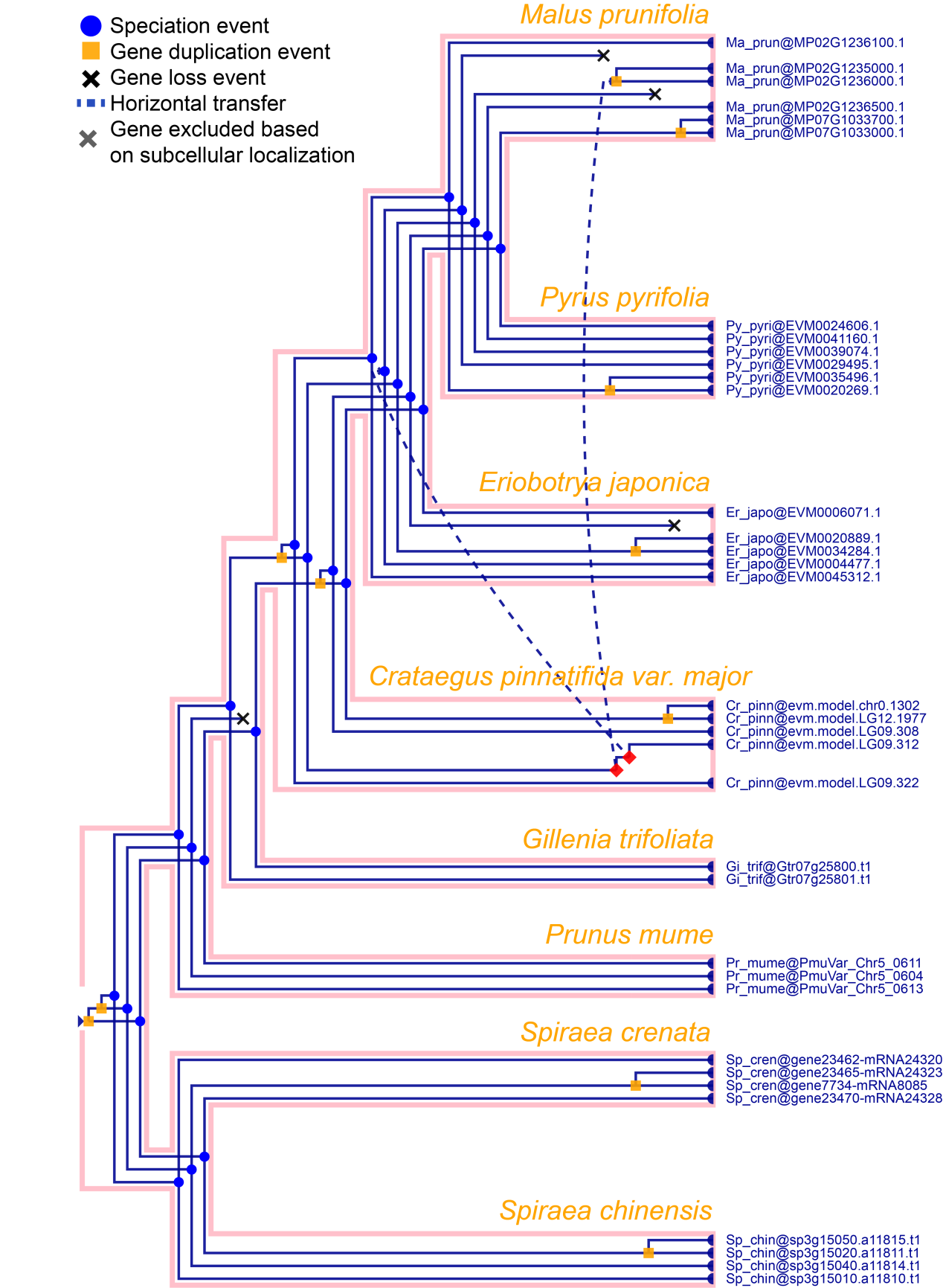


**Figure S47. Gene tree reconciliation and evolutionary history of the *BSH* gene family involved in the PAL route across eight Amygdaloideae species.** Annotation details are consistent with those shown in Fig. S38.


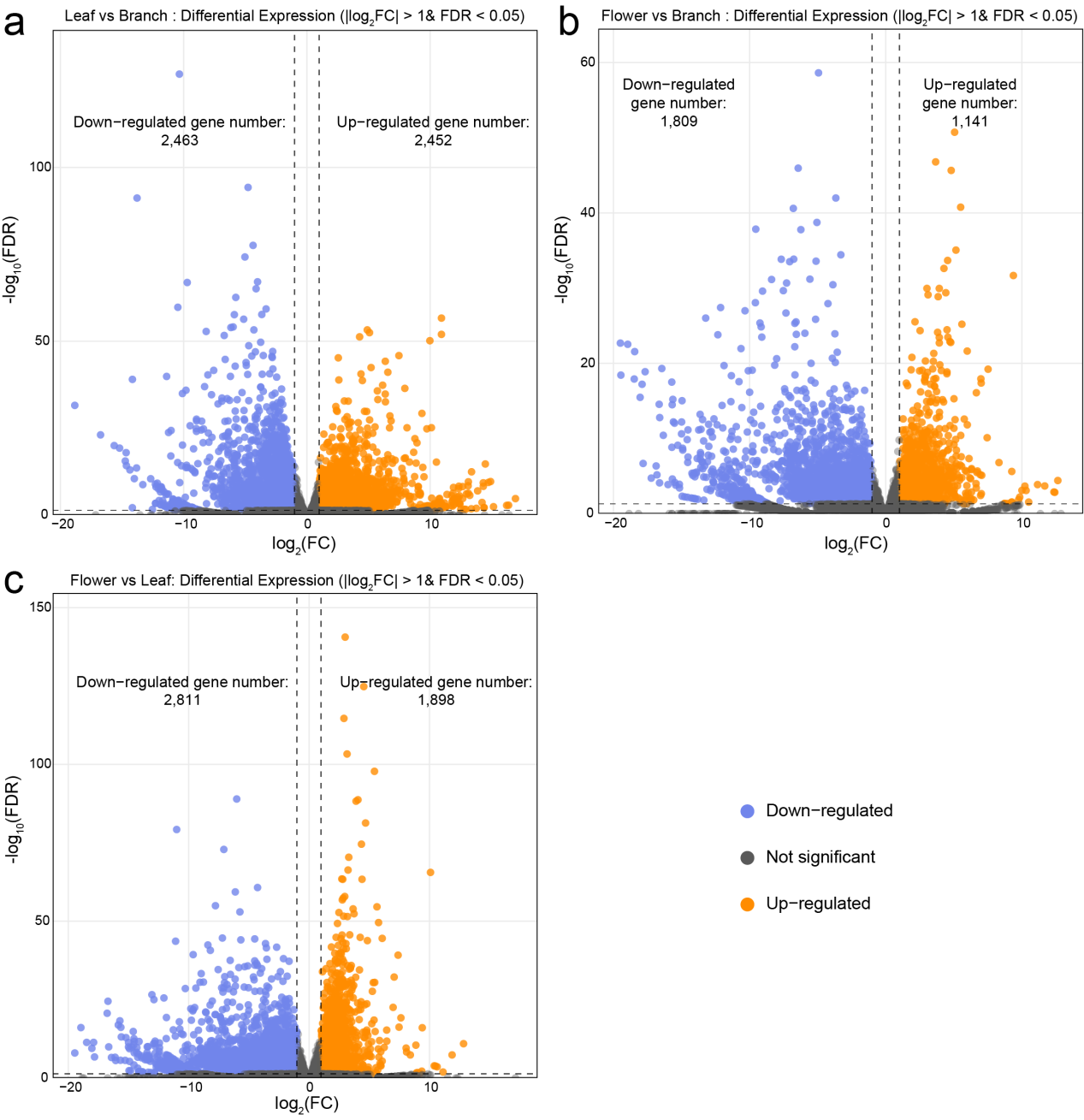


**Figure S48. Volcano plots of differential gene expression for Leaf vs Branch (a), Flower vs Branch (b), and Flower vs Leaf (c).** Each point represents one gene. The y-axis displays −log₁₀ false discovery rate/adjusted *p* value (−log₁₀FDR). Positive log₂ fold change (log₂FC) indicates higher expression in the first tissue relative to the second tissue, whereas negative log₂FC indicates lower expression. Orange points represent significantly up-regulated genes in the first tissue (log₂FC > 1 and FDR < 0.05), blue points represent significantly down-regulated genes (log₂FC < −1 and FDR < 0.05), and grey points indicate non-significant genes.


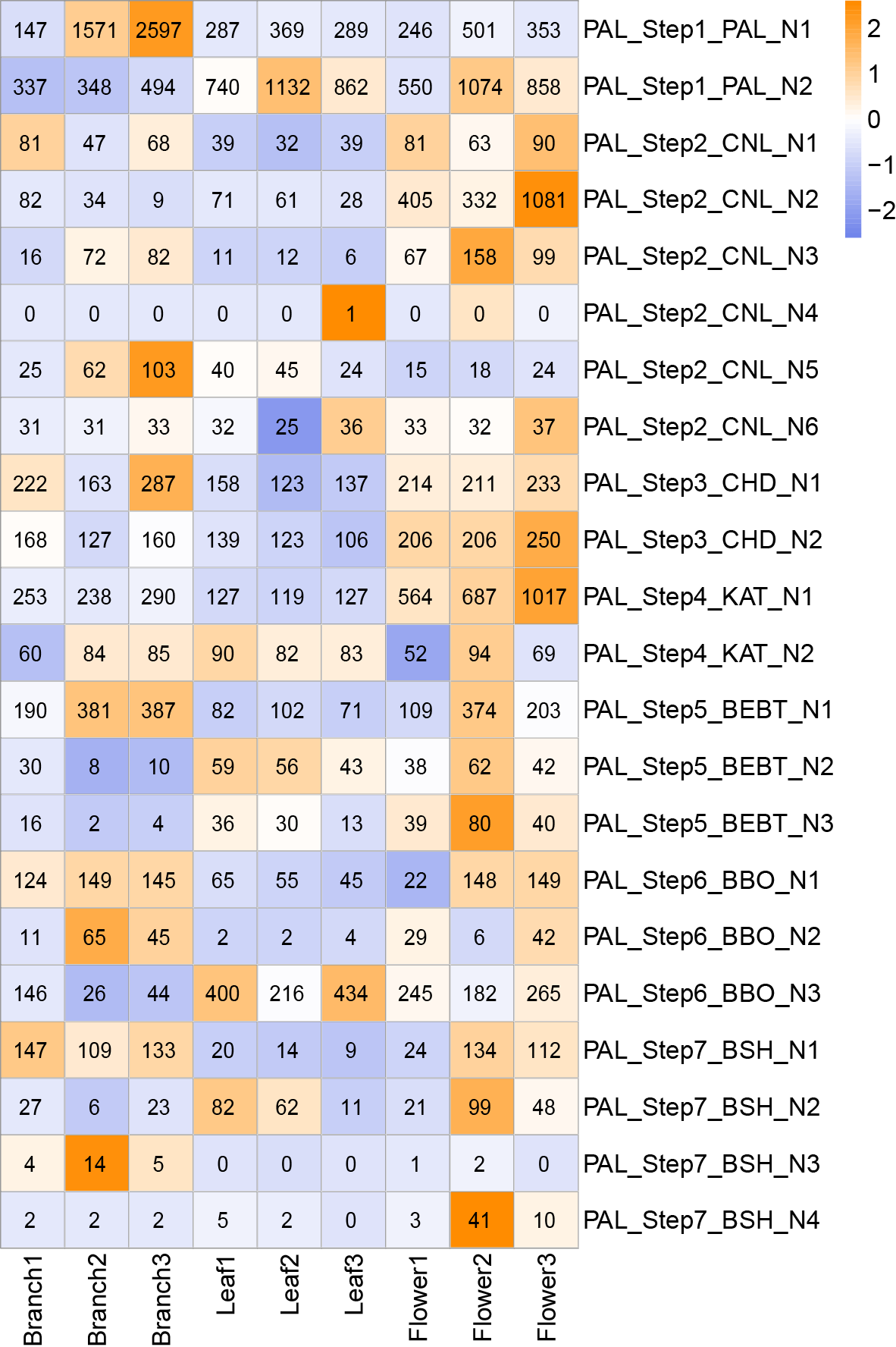


**Figure S49. Expression patterns of key enzymes involved in the PAL route in *S. chinensis*.** Expression values were normalized by row and visualized using Z-score transformation, with orange indicating higher expression and blue indicating lower expression relative to each gene. To facilitate interpretation of actual expression levels, CPM values are labeled directly in each cell of the heatmap.


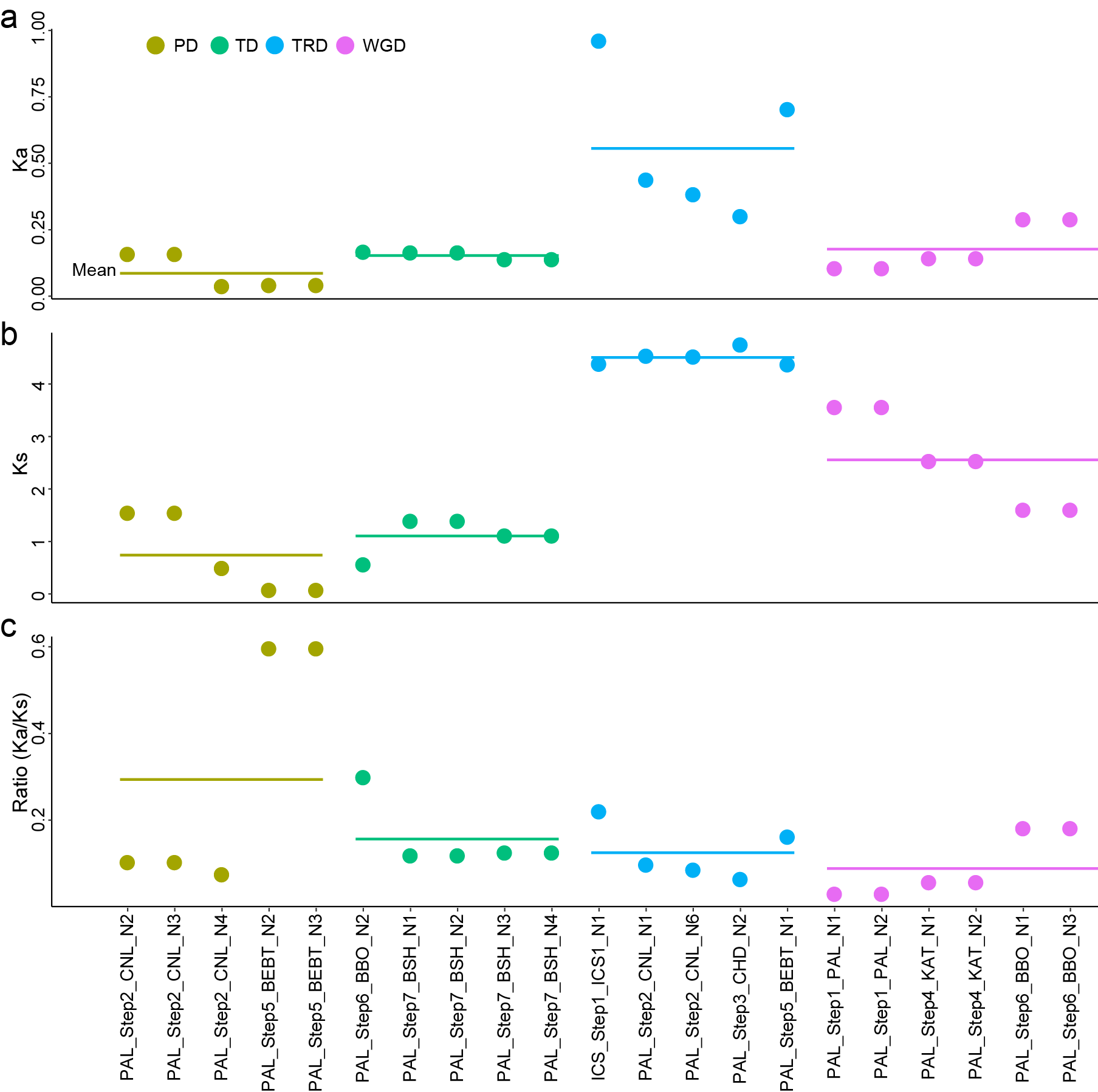


**Figure S50. Duplication-mode–dependent evolutionary rate variation of SA pathway genes in *S. chinensis*. (a)** Nonsynonymous substitution rates (Ka), **(b)** synonymous substitution rates (Ks), and **(c)** Ka/Ks ratios of duplicated genes involved in the SA biosynthetic pathway of *S. chinensis*. Genes are classified according to their duplication origin, including proximal duplication (PD), tandem duplication (TD), transposed duplication (TRD), and whole-genome duplication (WGD). Each dot represents an individual duplicated gene. Horizontal bars indicate the mean value for each duplication type.


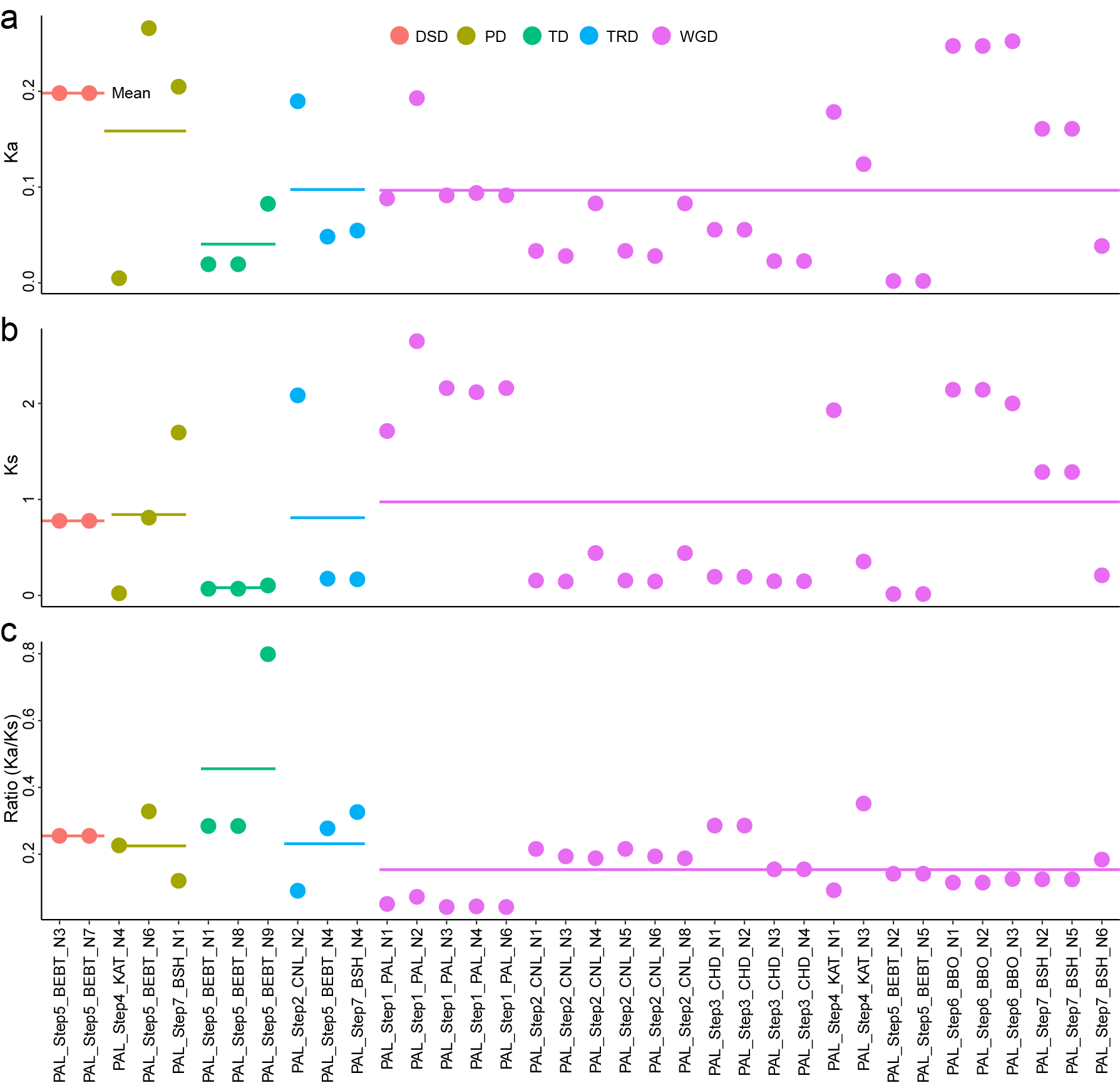


**Figure S51. Duplication-mode–dependent evolutionary rate variation of SA pathway genes in *Pyrus pyrifolia*. (a)** Nonsynonymous substitution rates (Ka), **(b)** synonymous substitution rates (Ks), and **(c)** Ka/Ks ratios of duplicated genes involved in the SA biosynthetic pathway of *S. chinensis*. Genes are classified according to their duplication origin, including dispersed duplication (DSD), proximal duplication (PD), tandem duplication (TD), transposed duplication (TRD), and whole-genome duplication (WGD). Each dot represents an individual duplicated gene. Horizontal bars indicate the mean value for each duplication type.


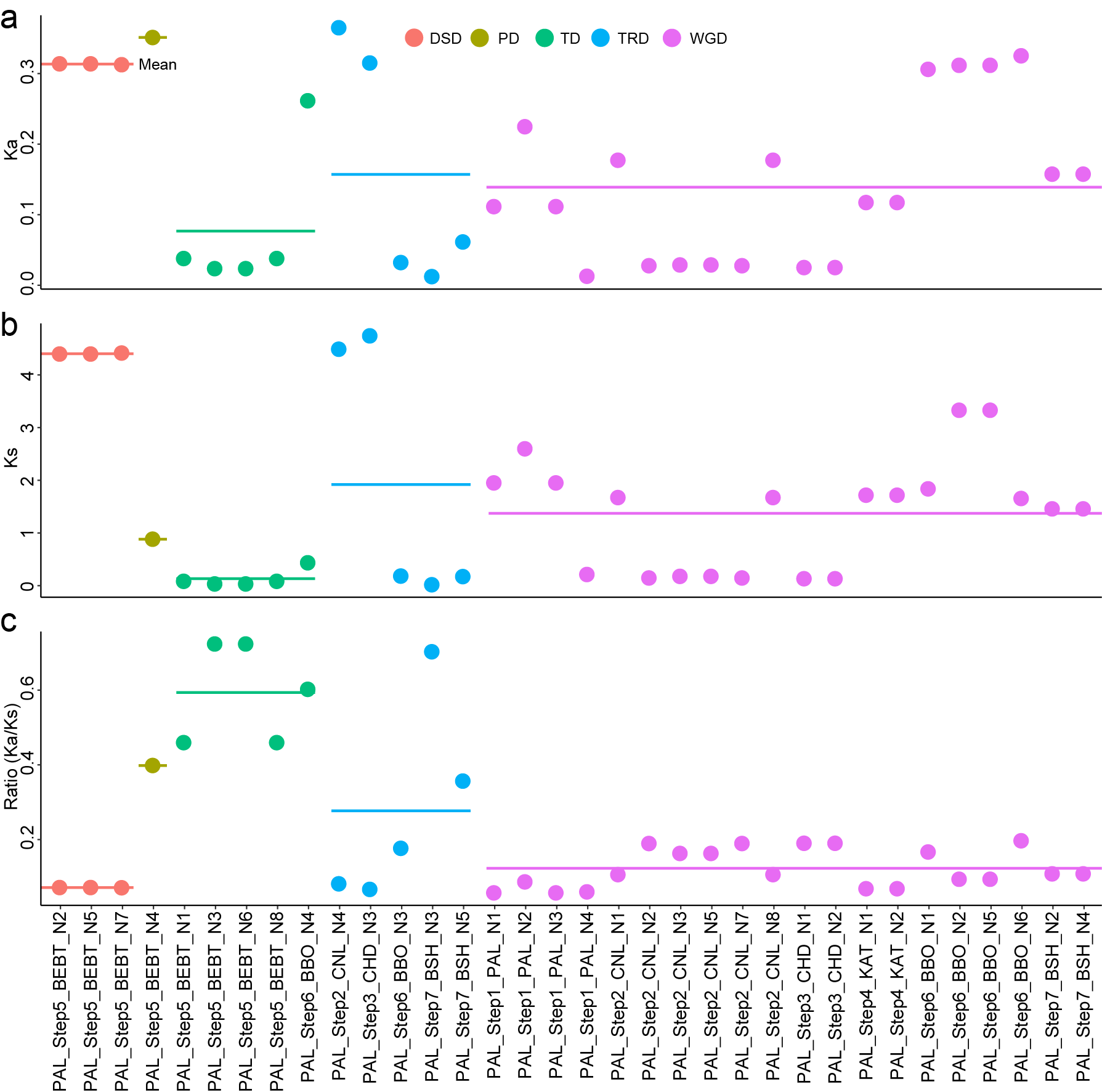


**Figure S52. Duplication-mode–dependent evolutionary rate variation of SA pathway genes in *Eriobotrya japonica*. (a)** Nonsynonymous substitution rates (Ka), **(b)** synonymous substitution rates (Ks), and **(c)** Ka/Ks ratios of duplicated genes involved in the SA biosynthetic pathway of *S. chinensis*. Genes are classified according to their duplication origin, including dispersed duplication (DSD), proximal duplication (PD), tandem duplication (TD), transposed duplication (TRD), and whole-genome duplication (WGD). Each dot represents an individual duplicated gene. Horizontal bars indicate the mean value for each duplication type.


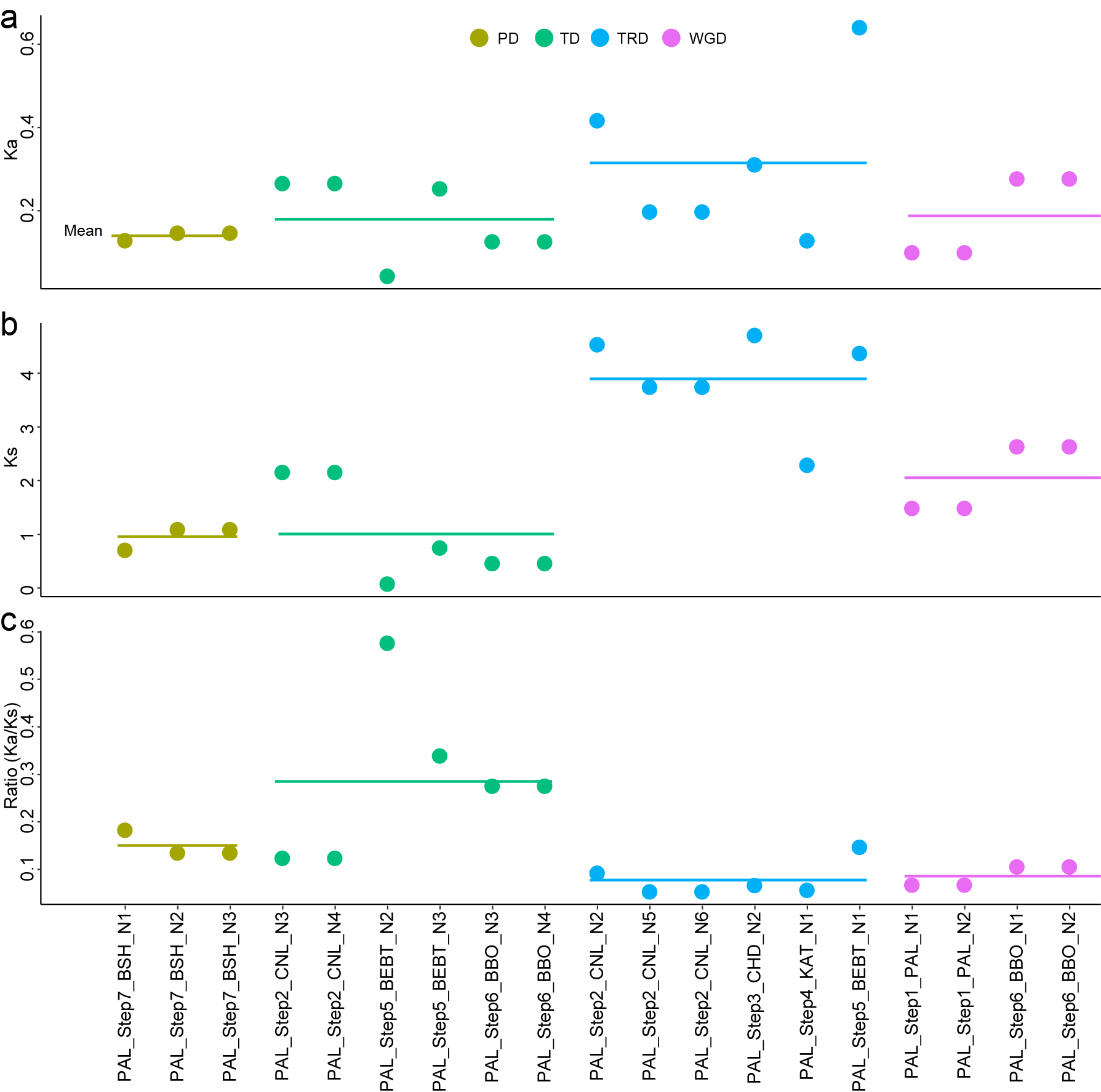


**Figure S53. Duplication-mode–dependent evolutionary rate variation of SA pathway genes in *Prunus mume*. (a)** Nonsynonymous substitution rates (Ka), **(b)** synonymous substitution rates (Ks), and **(c)** Ka/Ks ratios of duplicated genes involved in the SA biosynthetic pathway of *S. chinensis*. Genes are classified according to their duplication origin, including proximal duplication (PD), tandem duplication (TD), transposed duplication (TRD), and whole-genome duplication (WGD). Each dot represents an individual duplicated gene. Horizontal bars indicate the mean value for each duplication type.


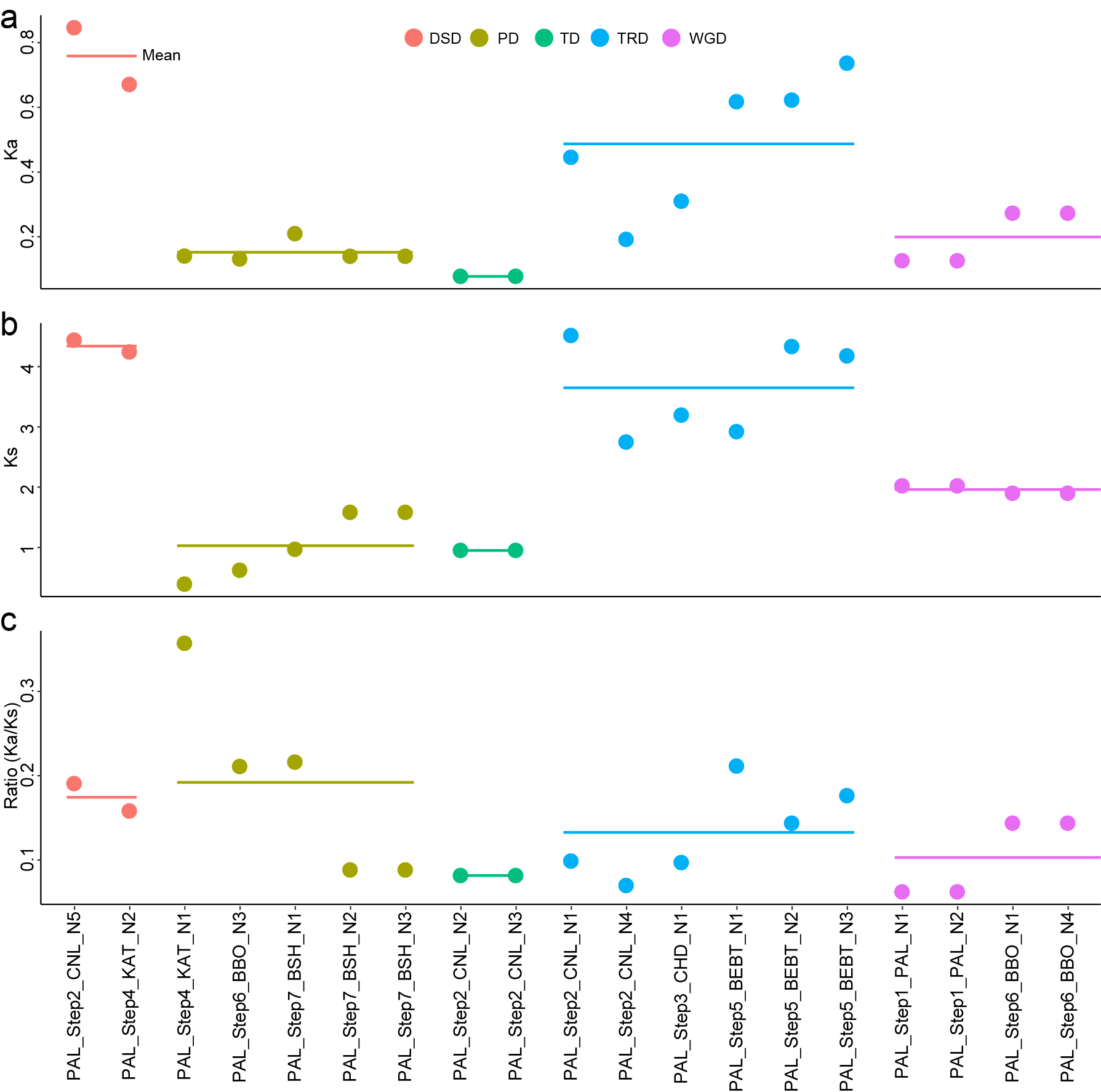


**Figure S54. Duplication-mode–dependent evolutionary rate variation of SA pathway genes in *Fragaria vesca*. (a)** Nonsynonymous substitution rates (Ka), **(b)** synonymous substitution rates (Ks), and **(c)** Ka/Ks ratios of duplicated genes involved in the SA biosynthetic pathway of *S. chinensis*. Genes are classified according to their duplication origin, including dispersed duplication (DSD), proximal duplication (PD), tandem duplication (TD), transposed duplication (TRD), and whole-genome duplication (WGD). Each dot represents an individual duplicated gene. Horizontal bars indicate the mean value for each duplication type.


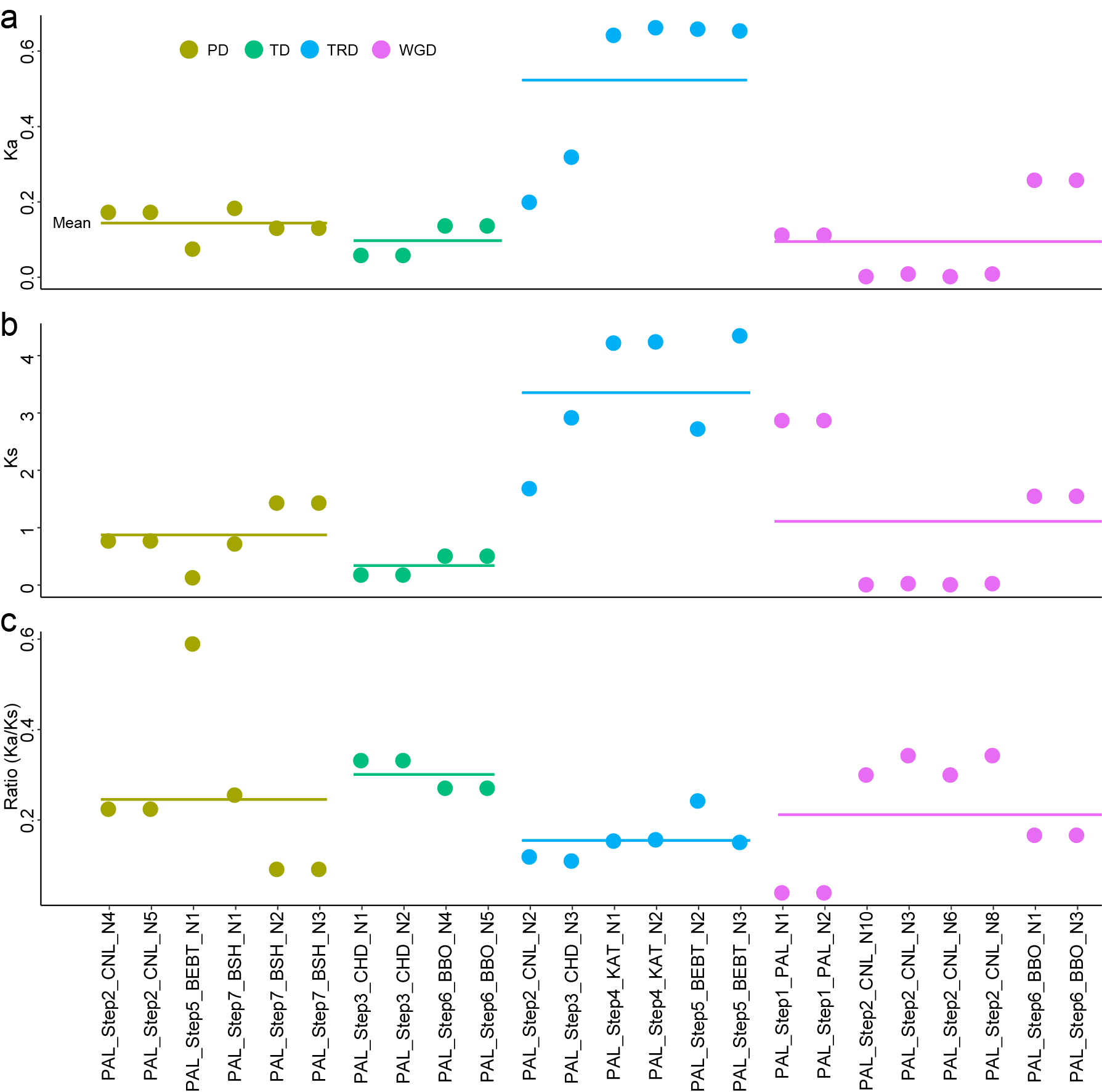


**Figure S55. Duplication-mode–dependent evolutionary rate variation of SA pathway genes in *Rubus idaeus*. (a)** Nonsynonymous substitution rates (Ka), **(b)** synonymous substitution rates (Ks), and **(c)** Ka/Ks ratios of duplicated genes involved in the SA biosynthetic pathway of *S. chinensis*. Genes are classified according to their duplication origin, including proximal duplication (PD), tandem duplication (TD), transposed duplication (TRD), and whole-genome duplication (WGD). Each dot represents an individual duplicated gene. Horizontal bars indicate the mean value for each duplication type.
